# Robust phenotyping strategies for evaluation of stem non-structural carbohydrates (NSC) in rice

**DOI:** 10.1093/jxb/erw375

**Published:** 2016-10-05

**Authors:** Diane R. Wang, Edward J. Wolfrum, Parminder Virk, Abdelbagi Ismail, Anthony J. Greenberg, Susan R. McCouch

**Affiliations:** ^1^Section of Plant Breeding and Genetics, School of Integrated Plant Sciences, Cornell University, Ithaca, NY 14853-1901, USA; ^2^Integrated Biorefinery Research Facility, National Renewable Energy Lab, Golden, CO 80401, USA; ^3^International Center for Tropical Agriculture, Km17 Recta Cali-Palmira, PO Box 6713, Cali, Colombia; ^4^International Rice Research Institute, Los Baños, Laguna, Philippines; ^5^Bayesic Research, Ithaca, NY 14850, USA

**Keywords:** Near-infrared spectroscopy, non-structural carbohydrates, *Oryza sativa*, partial least-squares, replication, yield potential.

## Abstract

Rice plants (*Oryza sativa*) accumulate excess photoassimilates in the form of non-structural carbohydrates (NSCs) in their stems prior to heading that can later be mobilized to supplement photosynthate production during grain-filling. Despite longstanding interest in stem NSC for rice improvement, the dynamics of NSC accumulation, remobilization, and re-accumulation that have genetic potential for optimization have not been systematically investigated. Here we conducted three pilot experiments to lay the groundwork for large-scale diversity studies on rice stem NSC. We assessed the relationship of stem NSC components with 21 agronomic traits in large-scale, tropical yield trials using 33 breeder-nominated lines, established an appropriate experimental design for future genetic studies using a Bayesian framework to sample sub-datasets from highly replicated greenhouse data using 36 genetically diverse genotypes, and used 434 phenotypically divergent rice stem samples to develop two partial least-squares (PLS) models using near-infrared (NIR) spectra for accurate, rapid prediction of rice stem starch, sucrose, and total non-structural carbohydrates. We find evidence that stem reserves are most critical for short-duration varieties and suggest that pre-heading stem NSC is worthy of further experimentation for breeding early maturing rice.

## Highlight

Experimental design and phenotyping methods were explored to enable efficient evaluation of rice stem non-structural carbohydrates for genetic studies. Evidence was found for phenology-dependent yield and stem NSC relationships.

## Introduction

Stem non-structural carbohydrates (NSCs) have long elicited interest from physiologists and breeders across a diversity of economically important grass species. By serving as a temporary sink to store excess photoassimilates during vegetative growth, the grass stem can also fulfill the role of a source organ during grain-filling and maturation, providing carbohydrates for these *in vivo* processes and/or in the form of harvestable end product (Slewinski, 2012). In sucrose-accumulating sugarcane, stems are collected principally for their soluble sugars to support bioenergy production and human consumption (Moore, 1995; Waclawovsky *et al.*, 2010). For perennial forage species such as ryegrass, post-grazing stem NSC reserves enable critical biomass regeneration for the next grazing period (Fulkerson and Donaghy, 2001). NSC reserves stored in the vegetative parts of perennial grasses during the fall are also important to winter survival of temperate species (Slewinski, 2012). For cereals cultivated for grain production, pre-anthesis stem carbohydrate stores can be remobilized to the grain to buffer yields against suboptimum environmental conditions that limit leaf photosynthesis. In subtropical climates where rice is grown as a ratoon crop, NSC reserves that remain in the stem after the first harvest determine the speed of subsequent vegetative re-growth, flowering, and grain-filling of the second crop (Slewinski, 2012) The evident diversity of roles that grass stem NSCs play across species suggests the potential to also optimize their capacities within species through breeding.

For *Oryza sativa* (cultivated Asian rice), knowledge about stem NSC’s direct link to yield performance and the genetic controls that underlie its dynamics are still superficial. Rice stems preferentially store starch and sucrose prior to heading, but lose these reserves rapidly following panicle exertion when grain-filling is prioritized energetically; these data support the idea that the stem experiences a sink-to-source transition at heading (Cock and Yoshida, 1972; Chen and Wang, 2008). There is also evidence of re-accumulation of stem NSCs as the panicle loses sink strength nearing grain-filling completion (Van Dat and Peterson, 1983; Kashiwagi *et al.*, 2006). From their evaluation of two near-isogenic genotypes (cv. Calrose 76 and its spontaneous mutant, ED7) to determine yield and carbohydrate-partitioning dynamics throughout grain-filling, Van Dat and Peterson postulated that pre-anthesis stem reserves may have been critical for yield realization in the short-duration genotype ED7, but not useful for Calrose 76. Despite general acceptance of the temporal and spatial patterns of rice stem NSC, estimates for the overall contribution of these stored carbohydrates to final grain yield vary widely across studies. While some document a significant contribution of stem reserves to final yield (up to 40%) (Van Dat and Peterson, 1983; Samonte *et al.*, 2001; Kanbe *et al.*, 2009), others report no such association, pointing to sink limitation (unpublished data cited in Setter *et al.*, 1994). Several studies show evidence of enhanced contribution to grain-filling during suboptimum conditions (e.g. water deficit or heat stress) coordinated with more rapid grain-filling and leaf senescence (Yang *et al.*, 2000, 2002; Kim *et al.*, 2011; Morita and Nakano, 2011). Management conditions that delay senescence (e.g. heavy nitrogen application) seem to have the opposite effect and suppress translocation of stem reserves, in effect decreasing their grain-filling contribution (Hirano *et al.*, 2005; Fu *et al.*, 2011; Pan *et al.*, 2011). Contrasting with wheat research in which the agronomic role of stem fructans was established early and now informs wheat physiological breeding (Bidinger *et al.*, 1977; Pask *et al.*, 2012; Reynolds *et al.*, 2012), studies on rice have not been able to clearly define aspects of stem reserves that are genetically tractable or capable of optimization and thereby valuable for varietal improvement.

The burgeoning availability of open-access genetic resources for rice (IRGSP, 2005; Jacquemin *et al.*, 2013; Li *et al.*, 2014; Schatz *et al.*, 2014; Duitama *et al.*, 2015; McCouch *et al.*, 2016) supports systematic large-scale studies to dissect the genetic architecture underlying stem NSC dynamics. The MSUv7 genome assembly (http://rice.plantbiology.msu.edu/) has been annotated with gene models across the 12 rice chromosomes, and over 100 predicted enzyme-coding genes have putative catalytic involvement in starch and sucrose biosynthesis, degradation, and transport (Dharmawardhana *et al.*, 2013). Despite these candidates and other genes involved in related developmental pathways (e.g. vascular bundle formation) that likely confer nuances to stem NSC, only very large quantitative trait loci (QTLs) that span many megabases of the rice genome have been discovered so far using bi-parental mapping populations.(Nagata *et al.*, 2002; Kashiwagi and Ishimaru, 2004; Kashiwagi *et al.*, 2006, 2008; Kanbe *et al.*, 2009). No QTLs have been reported in the literature using genome-wide association studies (GWAS) for rice stem NSC.

We consider two practical constraints to successful QTL identification for the suite of transient, dynamic traits associated with stem NSC. The first issue is that the experimental design parameters needed to effectively evaluate NSC for genetic studies have not been systematically explored. Design features such as the number of replicates required to adequately estimate the mean or dispersion have not been studied for rice stem NSC; the solution likely differs from highly heritable traits such as plant height or heading date, but this requires investigation. The second challenge is that the effort involved in traditional analytical chemistry techniques may be prohibitively laborious for larger-scale studies. Carbohydrates are typically assayed using a series of enzyme-mediated reactions to break down polymers into monomers that can then be measured using a UV-VIS spectrophotometer (Trinder, 1969). A previous attempt was made to apply near-infrared (NIR) spectra models to predict stem NSC in rice (Batten *et al.*, 1993), but most advances in the application of indirect methods for NSC determination have taken place in wheat (Ruuska *et al.*, 2006; Wang *et al.*, 2011; Dreccer *et al.*, 2014).

For this study, we began by examining the role of rice stem NSC under optimal growing conditions in irrigated lowland yield trials using a breeder-nominated panel of elite germplasm. We next phenotyped a set of diverse germplasm under greenhouse conditions to generate a highly replicated dataset from which to extract sub-datasets to establish optimal experimental design. Finally, we developed two partial least-squares (PLS) models to accurately predict rice stem constituents using NIR spectral data generated from a semi-automated Thermo-Antaris Autosampler as a high-throughput alternative to wet chemistry analysis. Our overall goal was to streamline a methodology to support evaluation of rice stem NSC for future genetic studies under controlled (greenhouse) conditions.

## Materials and methods

### Plant materials

Two sets of rice germplasm were evaluated in this study. The first was a breeder’s panel (BP), consisting of 33 elite accessions nominated by the International Rice Research Institute (IRRI) and PhilRice, evaluated under field conditions in Los Baños, Philippines. The panel consisted primarily of *indica* genotypes (*n*=25) along with two *tropical japonica* accessions, four *indica/tropical japonica* derivatives, and two entries that harbored introgressions from wild relatives. Entries were divided into maturity groups for analyses: Early (<120 days, 10 entries), Medium (120–129 days, 10 entries), and Late (≥130 days, 13 entries).

The second set of germplasm was a small diversity panel evaluated under greenhouse conditions in Ithaca, NY. This diversity set was comprised of 30 diverse *O. sativa* accessions (20 *indica* and 10 *tropical japonica*) along with six contrasting lines (described below) from the breeder’s panel. Germplasm information is summarized in Supplementary Tables S1 and S2 at *JXB* online.

### Selection of contrasting breeding lines for diversity study

To select multivariate contrasting genotypes out of the 33 accessions evaluated in the field, we used a principal component analysis (PCA) based on the line means for four NSC traits (starch levels at flowering and maturity, and sucrose levels at flowering and maturity) and hierarchical clustering with two linkage methods (complete and average). We selected six genotypes based on membership across clusters in both the complete linkage and average linkage hierarchical clustering trees and wide distribution across the PC1-PC2 plane resulting from the PCA (see Results). The chosen subset was: PS5 (HHZ12-DT10-SAL1-DT1; Green Super Rice), PS6 (IR60; *indica* variety with low chalkiness), PS13 (IR78049-25-2-2-2; *tropical japonica* New Plant Type), PS14 (IR78222-20-7-148-2-B; pyramided QTL for blast-resistance), PS22 (IRRI127; *indica* with tungro resistance), and PS37 (Teqing 1; *indica* from China).

### Genetic information

The six BP lines included in the diversity evaluation were genotyped along with 54 control varieties with known subpopulation identities using the genotyping-by-sequencing (GBS) platform at 96-plex with *ApeK1* enzyme digestion as the basis for assigning subpopulation designations. Using PCA, the six BP genotypes were analyzed along with 54 control varieties using 6125 genome-wide single-nucleotide polymorphism (SNP) markers that had 100% call rate on the sixty total accessions (Supplementary Fig. S5). For studying the effect of experimental replication on heritability estimates, we used publicly available genotype information from 700 000 SNPs generated using the high-density rice array (McCouch *et al.*, 2016). Publicly available genotypes were available for 31 of the 36 individuals in the diversity panel.

### Evaluation and sampling under field conditions

The breeder’s panel was planted in replicated yield trials using three replications during the 2012 dry season (2012DS) at the International Rice Research Institute in Los Baños, Philippines. The beginning of the growing season (January–March) displayed consistent precipitation and temperatures, while April at the IRRI Farm experienced significantly higher solar radiation and warmer maximum temperatures (IRRI, 2012). Each replication plot followed replicated yield trial standards (6 × 2m, 10 rows × 30 plants). Seeds were sown on 21 December 2011 and seedlings were transplanted on 10 January 2012. Irrigated lowland growing conditions were maintained and fertilizer was applied on the following dates: 6 January (basal application, 30-30-30 NPK), 31 January (topdress, urea 45 kg N ha^–1^), 16 February (topdress), and 29 February (topdress). Days to heading (DTH) was scored on the day that 50% of the individuals in a replication plot exerted panicles, and occurred from 14 February to 28 March. Seeds were harvested as plots matured, from 3 April to 30 April.

Four categories of traits were evaluated: phenological traits (days to heading, growth duration, grain-filling duration), resource allocation traits (biomass component dry weights, plant height, tiller and panicle number), physiological traits (flag leaf chlorophyll content at maturity, stem carbohydrates at heading and maturity, and senescence score at maturity), and yield components (yield, harvest index, percent grain-filling, 1000 grain weight, and harvest index). See Suppementary Table S3 for complete list and details on trait measurement methodologies. To sample plants for trait measurements at heading and maturity stages, five hills were chosen randomly from each plot per sampling point and pulled entirely out of the ground, while yield was measured on a 4-m^2^ harvested area per plot (equivalent to 100 hills) and harvest index was measured on a 1-m^2^ harvested area per plot (equivalent to 25 hills). All grain weight values are calibrated to 14% moisture content. For chlorophyll determination, five flag leaves were sampled per replication plot at maturity and pooled to give one value per replication plot. Leaves were freeze-dried and chlorophyll extracted overnight with cold 80% acetone aqueous solution. Absorbance was read at 663, 652, and 645 nm using a spectrophotometer and chlorophyll concentration in ppm (mg l^–1^) was calculated (as per Bruuinsma, 1963).

For carbohydrate sampling, all five randomly selected hills per plot sampled for trait evaluation on the day of flowering were combined. Panicles and leaf blades were immediately removed; stems were chopped manually using scissors into ~1-cm long pieces and subsequently subsampled for 20% of the original mass and flash-frozen in liquid nitrogen. These samples were then freeze-dried and finely ground using a sample mill. Subsamples of 200 mg of the freeze-dried, ground tissue were taken and subjected to carbohydrate analysis as per the Yoshida method (Cock and Yoshida, 1972). The proportion of total non-structural carbohydrate (TNC) remobilized from heading to maturity (*TNC*_*RMB*_) relative to the amount accumulated at heading was calculated as follows:

$$TNC_{RMB}=100\%\times \frac{\left(TNC_{hd}\times STM_{hd}-TNC_{mt}\times STM_{hd}\right)}{\left(TNC_{hd}\times STM_{hd}\right)}$$

where *TNC* is the percentage of total non-structural carbohydrates by dry weight at heading (hd) or maturity (mt), and *STM* is the dry weight of stems from five hills collected at heading or maturity. This is an indirect estimate of the TNC remobilized, as it reflects the net difference between heading and maturity. It therefore does not account for any changes that might occur between the two sampling points (e.g. re-accumulation of TNC at the end of grain-filling).

### Evaluation and sampling under greenhouse conditions

The small diversity panel was grown under greenhouse conditions at the Guterman Bioclimatic Laboratory in Ithaca, NY, during spring 2013 in a randomized complete block design with 20 replicates per accession (36 accessions total, see Plant Materials). Seeds were sown on 5 March and seedlings were transplanted into eight-inch (20-cm) pots set into flooded tanks 2 weeks after. Greenhouse conditions were managed using 11 hours light (29 °C)/13 hours dark (24 °C) at 55% constant humidity.

Days to heading was scored when the first panicle per plant emerged at least 50% out of its sheath and the following additional traits were evaluated: tiller number (counted at maturity), senescence score (according to IRRI’s Standard Evaluation System for rice), and stem dry weight measured on a tiller basis after removal of the panicle and leaf blades. Sampling for stem carbohydrates was done for a single sampled tiller per replicate per accession at two time points (heading and maturity), resulting in 20 biological replicates per accession.

For carbohydrate analysis, at each stem sampling point, tillers were excised from individual plants, and the leaf blades and panicle were removed. The remaining stems (culm and leaf sheath collectively) were microwaved immediately post-sampling to destroy respiration enzymes and then dried slowly in an oven at 65 °C over the course of several days until constant weight was achieved. Stem samples were initially coarse-ground using a conventional coffee grinder and then milled finely to a 0.2 mesh with an Udy Cyclone Sample Mill (Udy Corporation, Fort Collins, CO, USA). Subsamples of ~25 mg (exact weights recorded) were measured into 1.2-ml tubes in a 96-tube format and assayed for carbohydrates: sugars were extracted using 80% EtOH (and sucroses broken down via invertase) and the remaining starches were gelatinized and digested into glucose using amyloglucosidase/α-amylase. All carbohydrates were assayed utilizing peroxidase and glucose oxidase via the Trinder reaction and compared against known standards of glucose and sucrose at 490 nm using a 96-well plate-reading UV-VIS spectrophotometer (Trinder, 1969).

### Near-infrared spectral measurements

A total of 434 samples were used in the NIRS calibration study. These samples were selected in two batches. The first batch (*n*=277) was chosen to span the range of measured wet chemistry values in the diversity panel experiment with representation across the 36 different genotypes and two sampling points. The second batch (*n*=157, also with wet chemistry data) were selected randomly across genotypes and sampling points in two experiments: 357 samples from the current diversity study (germplasm described above) and 77 samples from a separate unpublished evaluation on a set of chromosome segment substitution lines (germplasm described in Arbelaez *et al.*, 2015). Choosing from the diversity study allowed us to sample across genetically divergent individuals that may display different background spectral signatures, while selecting across experiments conducted in two years allowed us to sample across environmental differences that may impact background spectral variation. Samples were packed in 2-dram borosilicate scintillation vials and scanned using a Thermo Antaris II FT-NIR Spectrometer with a 40-position autosampler carousel (Thermo Fisher Scientific, Waltham, MA). Each sample was scanned 128 times (wave number range: 3300–12 000 cm^−1^).

### Statistical analysis

All statistical analyses were coded and carried out in R (https://www.R-project.org/). Bayesian approaches were used for experimental design optimization and subsampling. These methods are fully described in Suppementary Data S1 and S2. These analyses involved nine traits (DTH, days to heading; STCH_HD, starch, heading; SUC_HD, sucrose, heading; SEN, senescence score; TIL, tiller number; WT, stem weight; GLC_HV, glucose, maturity; SUC_HV, sucrose, maturity; STCH_HV, starch, maturity). Model fitting for NIR data was undertaken using functions from the following R packages: signal (http://r-forge.r-project.org/projects/signal/, accessed 30 September 2016), prospectr (https://cran.r-project.org/web/packages/prospectr/, accessed 30 September 2016), and pls (https://cran.r-project.org/web/packages/pls/, accessed 30 September 2016). For calibration, resultant spectral data were first limited to the 4000–9000 wave-number range and subjected to mathematical pre-treatment using the standard-normal-variate (SNV) scatter correction and a first-derivative Savitzky–Golay smoothing (*n*=25 points). Using these pre-treated spectra, the 434 samples were divided into a calibration set (*n*=300) and an external validation set (*n*=134) using the Kennard–Stone algorithm. Two partial least-square (PLS) models were developed: a PLS-1 model and a PLS-2 model. The PLS-1 model predicted a single response variable, total non-structural carbohydrate (TNC = starch + sucrose). The more generalized PLS-2 model predicted two variables, starch and sucrose, as a single multivariate model. Small subsets of the 300 calibration samples were excluded as outliers in the final models (18 for the PLS-1 model and 17 for the PLS-2 model). These outliers were identified based on the difference between initial prediction of the fully cross-validated model and actual wet chemistry, and excluded if they were more than two times the RMSECV value for the initial model. Root mean squared errors for calibration (RMSEC), for full cross-validation (RMSECV), and for prediction of an independent test set (RMSEP) were calculated using the generalized equation:

$$RMSE=\sqrt{\frac{1}{N}\Sigma {\left(\hat{Y_{i}}-Y_{i,\;ref}\right)}^{2}}$$

where *N* is the number of samples in each population, $\hat{Y_{i}}$ is the predicted value, and $Y_{i,\;ref}$ is the measured value. Reference method (i.e. wet chemistry) uncertainties were estimated as twice the average standard deviation (as per Wolfrum *et al.*, 2013) on an external set of material (*n*=150) using triplicated technical replicates.

## Results

### Correlation blocks of agronomic characteristics in breeder-nominated lines

**Fig. 1.**
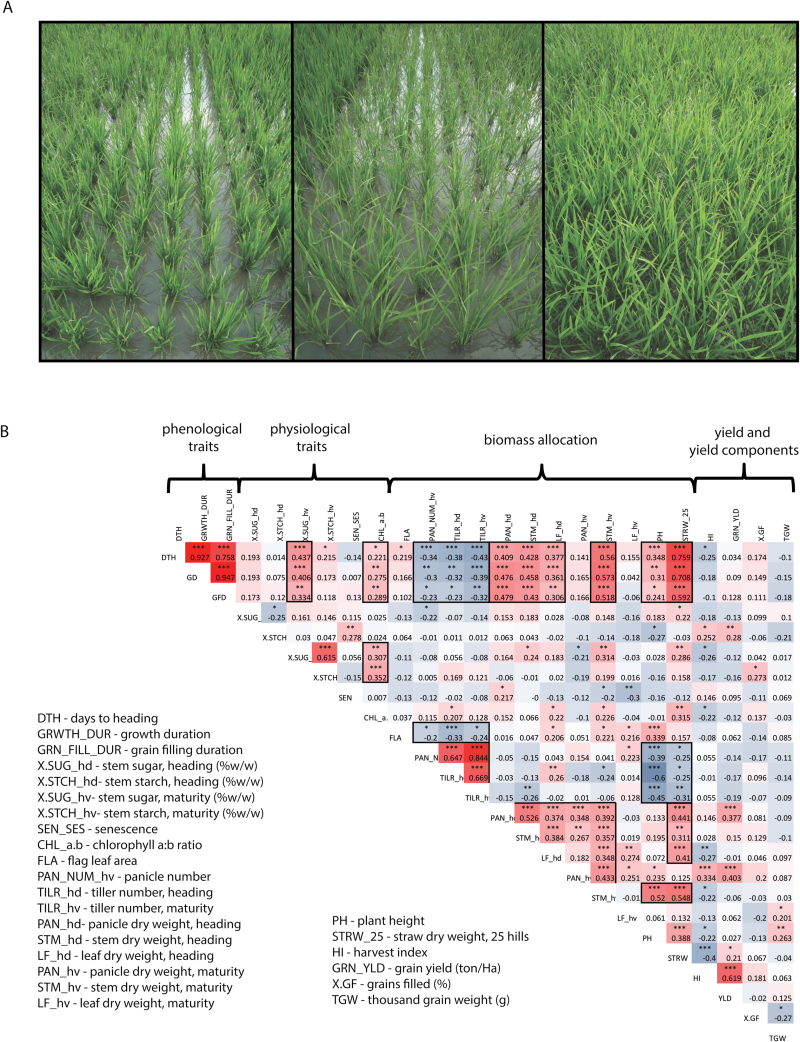
Phenotyping of the breeder’s panel in a replicated yield trial. (A) Breeder’s panel (BP) entries exhibited a wide range of phenotypic variation for many traits including developmental rate, as depicted by these images of the field trial taken at the same time point in the same replicate field for three entries. From left to right: BP 30, BP 6, and BP 31. (B) Correlation matrix of all directly measured traits (*P<0.05; **P<0.01, ***P<0.001). Red and blue squares indicate positive and negative correlations, respectively.

During the 2012 dry season the breeder’s panel exhibited wide variation in growth duration, which spanned from 104 to 141 d after sowing, and in other growth-related phenotypes (e.g. canopy growth rate Fig. 1A). Evidence of strong phenological linkage was observed for biomass traits. Days to heading, growth duration, and grain-filling duration were positively correlated with dry weight measurements (panicle weight, stem weight, leaf weight at heading, stem weight at maturity, and 25-hill straw weight at maturity), while these same phenological measures were negatively correlated with ‘count’ data (panicle number, tiller number at heading, and maturity) (see Fig. 1B for correlation blocks). Flag leaf area, plant height, and straw dry weight at maturity were also negatively associated with these count traits. There was no relationship between phenology and yield or yield components except for a weak negative correlation of days to heading and harvest index, an indirect outcome of the positive relationship between phenology and straw biomass, which contributes to the denominator of harvest index. Of the stem NSC traits, we observed a highly significant association between phenological measurements and stem sucrose at maturity, indicating that, in this panel, observations with a longer growing season either retained or re-accumulated more stem sucrose.

Out of the panel, BP30 (cv. IR60) had the lowest yield with an average of 3.72 ton ha^–1^ while BP 26 (cv. Teqing) had the highest yield with an average of 6.9 ton ha^–1^. Not including seed weight-related traits that are simply alternative expressions of yield (e.g. harvest index and panicle weight at harvest) we found two traits significantly correlated with grain yield: stem starch at heading (potential source for grain-filling) and panicle weight at heading (potential sink size for grain-filling). Interestingly, we also observed a significant negative relationship between thousand grain weight and percentage of grains filled, meaning entries with heavier, completely filled grains tended to have a smaller proportion of filled grains overall.

### Linkage between stem NSC and yield performance is strongest for short-duration rice

At heading, the BP accumulated an average of 16.7% total non-structural carbohydrates (TNCs) and retained 7.7% at physiological maturity (Suppementary Table S1). Observed differences in stem NSC at heading and at maturity provided evidence that patterns of starch accumulation and remobilization were synchronized with development and differed significantly across the two sampling points (paired *t*-test: *P*<2.2 × 10^–16^). Stem soluble sugar levels, on the other hand, were not different between heading and maturity.

Despite the fact that all entries were adapted to tropical conditions and selected for this study because they play key roles in irrigated lowland rice breeding, there was a surprisingly wide range of phenotypic variation with respect to stem NSC across the BP. At heading, BP 11 (cv. BR29, a Bangladeshi *indica* mega-variety) accumulated the least amount of TNC (8.62%) while BP 25 (cv. Teqing Acc. IRGC78727, a Chinese *indica*) accumulated the most at 24.62%. While most entries had lost most of their stem reserves by the end of the season, BP 18 (cv. NSIC212, an *indica* high-yielding variety released in the Philippines) harbored 13.11% TNC at maturity, due to high levels of soluble sugars.

**Fig. 2.**
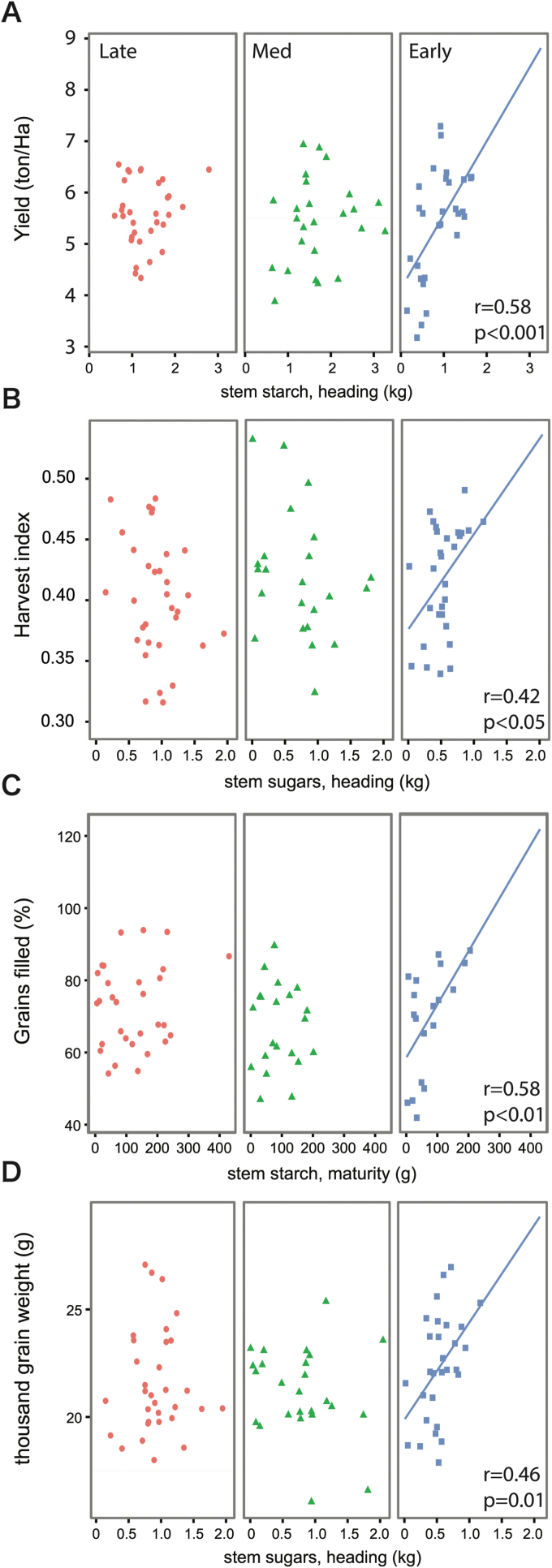
Phenology-dependent trait relationships. Early (squares), Medium (triangles), and Late (circles) entries displayed contrasting relationships of NSC with yield traits for four yield components: (A) yield, (B) harvest index, (C) grain-filling percentage, and (D) thousand-grain weight. (This figure is available in color at *JXB* online.)

Although *per se* effects of growth duration or flowering time were not observed for NSC traits or yield traits, we wondered if relationships between NSC and yield might vary due to phenology, as previously suggested by Van Dat and Peterson (1983). We addressed this question by grouping entries into three maturity groups, Early, Medium, and Late (see Methods for definitions). To further investigate the positive relationship observed between grain yield and relative starch at heading, we re-expressed relative stem NSC traits (% w/w) in absolute terms (dry weight per five hills sampled) as these are more appropriate representations of the total carbon incorporated into non-structural components of biomass. Relationships between NSC and the four yield components studied (yield, harvest index, percent grain filling, and thousand-grain weight) differed significantly across maturity groups. Analyzing the Early maturity group on its own revealed significant associations of at least one NSC component for every yield trait, while isolating the Medium and Late groups uncovered no further relationships between stem NSC and yield components (Fig. 2). One surprising outcome, given that yield, harvest index, and thousand-grain weight were all associated with NSC traits at heading, was that grain-filling percentage was correlated with NSC at maturity.

To explore the hypothesis that there might be a physiological trade-off between carbohydrate remobilization and the ‘stay-green’ phenotype at maturity, we examined the distribution of starch remobilization across senescence classes (see Supplementary Table S3 for scoring method). The distribution of TNC remobilized shifted upwards with increasing senescence (Supplementary Fig. S2), and a significant difference in the means of Group 3 (the most ‘stay-green’) and Group 9 (the most senescent) was observed (one-tailed Welch’s *t*-test, *P*=0.02). This result suggests that there is a negative physiological linkage between carbohydrate remobilization and the stay-green condition in these lines, a relevant finding given that both traits are targets of interest for IRRI’s irrigated lowland breeding program.

### Multivariate classification of the breeder’s panel by stem NSC components

**Fig. 3.**
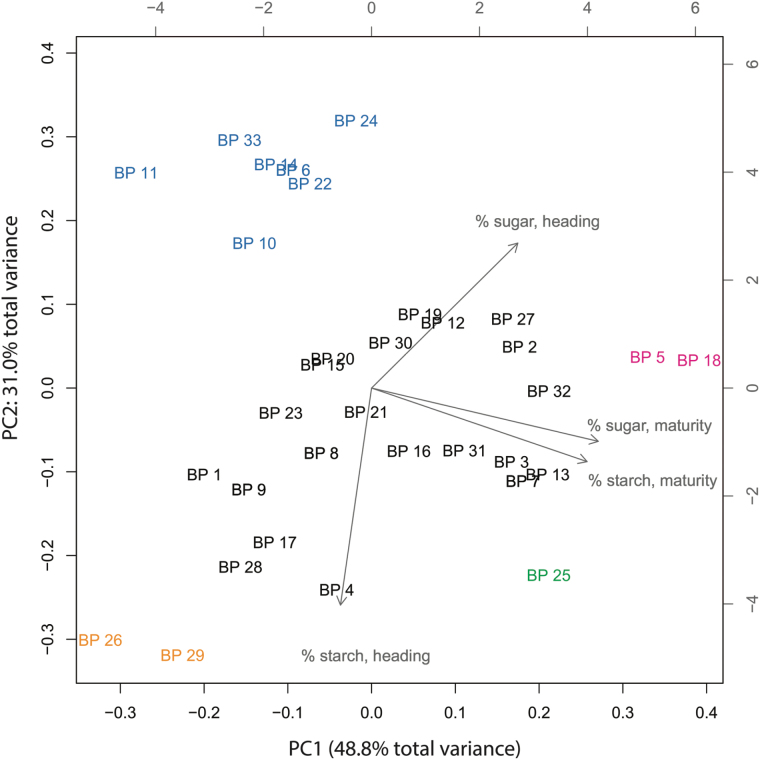
Principal component analysis (PCA) of the breeder’s panel. PCA was performed using mean NSC trait data, and trait loadings are indicated by gray arrows. Individuals (breeding lines) are colored according to groupings, defined by visual inspection of the PCA biplot: black text indicates the main cluster of individuals while blue, yellow, pink, and green text define minor clusters.

Principal component analysis (PCA) using stem NSC components as variables revealed that PC1 and PC2 collectively explained ~80% of the total phenotypic variance observed in the 33 entries of the breeder’s panel. Mapping the trait vectors onto the PC1–PC2 plane showed that PC1 was most aligned with stem NSC levels at maturity while PC2 was nearly collinear with the percentage of stem NSC at heading. Most entries clustered together (Fig. 3, black individuals), but 12 fell beyond the main group in the PC1–PC2 plane. Of these, seven formed a second group (blue individuals, Fig. 3) that was characterized by low starch levels at heading and low starch and sugar at maturity. At the other end of the PC2 axis were two individuals, BP 26 and BP 29 (yellow individuals, Fig. 3), which also retained little NSC at maturity but accumulated a large amount of starch at heading. These two entries represent the best ‘remobilizers’ of stem carbohydrates in the panel due to the large net loss of NSC between heading and maturity. In contrast, BP 5 and BP 18 (pink, Fig. 3) retained nearly the same amount of TNC at maturity as they had accumulated at heading, indicating either lack of remobilization or a re-accumulation at maturity. Interestingly, BP 25 (green, Fig. 3) and BP 26, which are different accessions of the same Teqing variety, did not cluster with each other as we had expected; both accumulated the same amount of starch at heading but BP 25 had much more sucrose at heading and maturity. Upon closer inspection we discovered that in our yield trial BP 25 took an average of 4 d longer to flower than BP 26 but matured in the same time, indicating a faster grain-filling period. It was also less senescent at maturity than BP 26. Hierarchical clustering using the average linkage method supported the PCA findings overall (Supplementary Fig. S1). There was no significant association between multivariate clustering by stem NSC components and other metadata on the rice accessions, such as subpopulation identity (*indica* vs. *tropical japonica*) or line classification (breeding line vs. released variety).

### NSC variation across diverse rice germplasm under greenhouse conditions

In contrast with the results of the field evaluation of the IRRI BP, no coordinated net starch loss was observed in the greenhouse trial of diverse germplasm (see Supplementary Table S3 for germplasm details); distributions of starch levels at heading did not differ significantly from overall starch distribution at maturity for any of the six BP lines selected for greenhouse evaluation (Supplementary Fig. S3A, D; see Methods for selection process). This may be due to sustained stem starch deposition throughout grain-filling due to continuous fertilization in our greenhouse management conditions in conjunction with lower competition for radiation associated with spaced planting. Sugar level, however, was significantly lower at maturity than at heading in the greenhouse (Supplementary Fig. S3B, E). Relative to 20 diverse rice accessions that were concurrently evaluated in the greenhouse, the IRRI BP lines accumulated more sucrose at heading but retained more starch at maturity, and the IRRI lines tended to cluster together at one tail of the phenotypic spectrum (Supplementary Fig. S4B, C).

Because rice has a deeply stratified subpopulation structure, we next wanted to compare trait distributions of the six BP entries directly against diverse germplasm that shared their genetic grouping. To determine the subpopulation identity of the six BP entries, we genotyped them using genotyping-by-sequencing (GBS) and analyzed the accessions along with 54 controls with known subpopulation identity (Supplementary Fig. S5). Out of the six BP lines, four (BP 14, BP 25, BP 29, BP 30) belonged to the *indica* subpopulation, reflecting this subpopulation’s importance to irrigated lowland rice breeding. Of the remaining two, BP 6 was classified as an admixed *indica* + *tropical japonica* and BP 5 was an admixed *temperate japonica* + *tropical japonica*. These results are consistent with pedigree information of BP 6 (*indica* × *tropical japonica* derived for blast resistance) and BP 5 (*temperate* × *tropical japonica*-derived New Plant Type). We found a significant difference between the mean performance of BP *indica*s and the more diverse *indica*s for levels of starch at heading, sucrose at heading, and starch at maturity (*P*<0.01 for all, Welch’s *t*-test). A significant difference was also observed in the mean of the BP *tropical japonica* and the more diverse *tropical japonica*s for levels of both starch and sucrose at heading and maturity (*P*<0.01 for all). This supports previous observations that, under greenhouse conditions, these breeding lines sample only a portion of the phenotypic variation found in an expanded panel of diverse rice germplasm, despite the wide range of NSC variation observed in the BP.

### Effect of experimental replication on genetic parameters of rice stem NSC

We next carried out a greenhouse evaluation of stem NSC in diverse rice accessions using 20 replicates per accession. The highly replicated nature of this study allowed us to explore questions related to experimental design to understand the effect of replicate size (RS) on estimates of distribution and genetic parameters for stem NSC traits. Using these data in conjunction with genetic information on 700 000 genome-wide SNPs from the high-density rice array (McCouch *et al.*, 2016), we took a Bayesian approach to estimate heritability (broad and narrow), genetic and environmental correlations, line mean, genomic estimated breeding value (GEBV), line dispersion parameters, and variance components (additive, non-additive, and error). Narrow-sense heritability estimates for starch and sucrose at heading were 0.56 and 0.61, respectively, while at maturity they were slightly lower at 0.49 (starch) and 0.58 (sucrose) (Table 1). Genetic and environmental correlations of trait pairs are summarized in Table 2.

**Table 1.** Heritability of rice stem NSC traits. Point estimates for narrow-sense heritability of stem NSC traits are shown here. Values are calculated based on genotypic data on 700 000 SNPs on 31 *indica* and *tropical japonica* individuals and phenotype data from 20 biological replicates per accession under greenhouse conditions. The labels ‘hd’ and ‘mt’ indicate heading and maturity sampling points, respectively.

Trait	*h* ^ 2 ^
Starch, hd	0.56
Starch, mt	0.49
Sucrose, hd	0.61
Sucrose, mt	0.58

**Table 2.** Genetic and environmental correlations for full replicate dataset. In each trait–trait pairwise sector of the matrix, the middle number indicates the point estimate while the top and bottom values indicate, respectively, the upper and lower credible interval bounds resulting from Bayesian analyses with Gaussian error distributions. Estimation using Bayesian methods with Student’s *t* error distribution yielded similar results. DTH, days to heading; hd, heading; mt, maturity.

	DTH	Starch, hd	Sucrose, hd	Tiller no.	Stem wt	Sucrose, mt	Starch, mt
**DTH**	1	0.792	0.745	0.706	0.631	0.718	0.849
	1	0.468	0.333	0.415	0.337	0.291	0.617
	1	–0.197	–0.33	–0.0241	–0.0314	–0.381	0.0719
**Starch, hd**	0.134	1	0.709	0.689	0.799	0.68	0.805
	0.0878	1	0.191	0.222	0.469	0.0777	0.464
	0.0418	1	–0.533	–0.42	–0.196	–0.586	–0.283
**Sucrose, hd**	0.15	0.234	1	0.752	0.695	0.732	0.684
	0.0604	0.199	1	0.341	0.218	0.247	0.123
	–0.0262	0.168	1	–0.37	–0.442	–0.554	–0.553
**Tiller no.**	0.141	0.186	0.13	1	0.516	0.665	0.567
	0.0541	0.105	0.0446	1	0.0606	0.179	0.0417
	–0.033	0.0173	–0.0406	1	–0.369	–0.484	–0.498
**Stem wt**	0.112	0.131	0.192	–0.0569	1	0.741	0.807
	0.0282	0.0466	0.108	–0.111	1	0.326	0.509
	–0.0567	–0.039	0.0232	–0.162	1	–0.36	–0.0605
**Sucrose, mt**	0.0476	0.072	0.193	0.104	0.239	1	0.667
	–0.0348	–0.0131	0.115	0.0167	0.168	1	0.111
	–0.124	–0.0978	0.0246	–0.0646	0.0969	1	–0.551
**Starch, mt**	0.203	0.116	0.217	0.00827	0.617	0.31	1
	0.119	0.0317	0.136	–0.0774	0.562	0.278	1
	0.0328	–0.0544	0.0527	–0.161	0.503	0.251	1

**Fig. 4.**
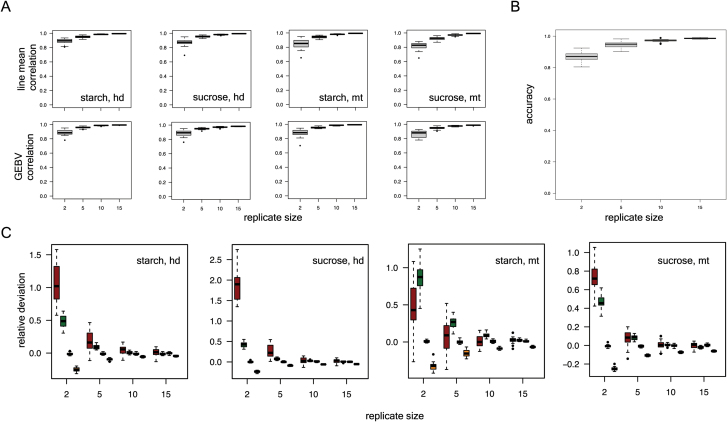
Effect of experimental replication on trait parameters. Effect of replicate size on (A) line mean and GEBV, (B) genetic correlation, and (C) variance component and narrow-sense heritability estimates (boxes from left to right within each replicate size indicate: error variance, non-additive genetic variance, additive genetic variance, heritability). (This figure is available in color at *JXB* online.)

To evaluate the effect of experimental replication size on trait parameters, we simulated at four different replicate sizes (RS = 2, 5, 10, 15) by subsampling from the full dataset (see Methods). With respect to line mean, improvements in accuracy (correlation between subsampled data and full dataset) were observed between RS=2 and RS=5 for starch and sucrose phenotypes, while there was not much gain in accuracy beyond five replicates (Fig. 4A). A similar trend was observed for GEBV estimates, except for starch at maturity, which benefitted from an RS increase up to 10. In addition to point estimates, we were interested in the effect of RS on uncertainty estimates. We chose the coefficient of variation (CV) to describe trait dispersion and found that an RS of at least 10 was necessary to come within 1.5 times the full set CV estimate (Supplementary Data S2). For genetic correlation estimates, an increase from two to five replicates improved the median accuracy but an RS of 10 yielded a much tighter distribution of accuracies compared with an RS of five (Fig. 4B). Finally, we used our highly replicated data to inquire about the effect of replicate size on variance component estimates and narrow-sense heritability (Fig. 4C). Analysis of relative deviation of subsampled data versus full data value revealed that error variances were overestimated at small replicate sizes while narrow-sense heritabilities were underestimated when compared to corresponding values from the full dataset for starch and sucrose traits.

### Utility of NIR spectral data to predict NSC in rice stem samples

**Fig. 5.**
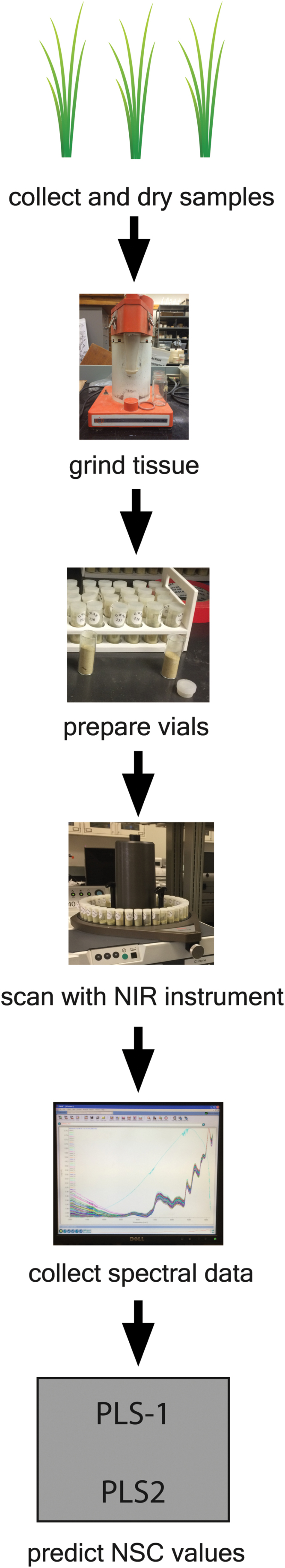
NIR protocol for assaying rice stem NSC. Steps taken for using near-infrared spectroscopy for rapid determination of rice stem NSC components. (This figure is available in color at *JXB* online.)

**Fig. 6.**
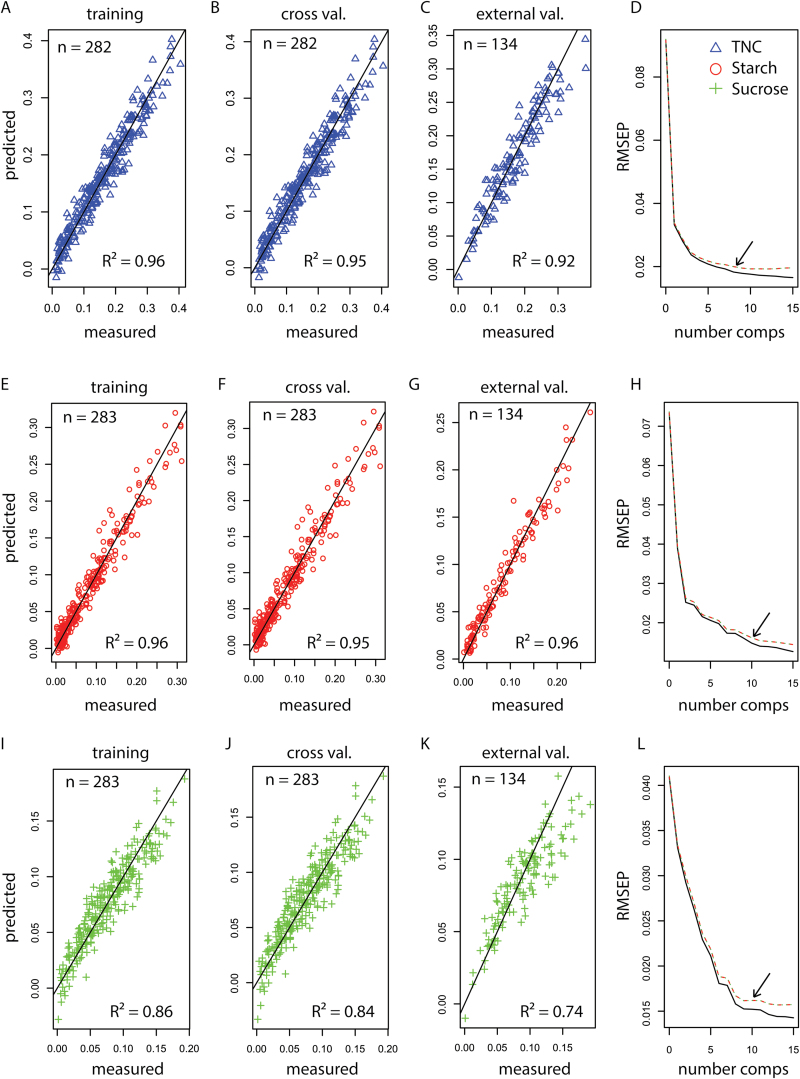
Results from NIR partial least-squares (PLS) calibration models. Predicted vs. measured values for training, cross validation, and external validation sets of the PLS-1 TNC model (A–C), PLS-2 model for starch (E–G), and PLS-2 model for sucrose (I–K). Root mean square error prediction (RMSEP) values as a function of model component number for PLS-1 TNC (D), PLS-2 starch (H), and PLS-2 sucrose (L). Black arrows in the RMSEP panels indicate the number of components included in the final calibration models. (This figure is available in color at *JXB* online.)

As NIR models have proven useful for assaying wheat stem fructans (Wang *et al.*, 2011), we assessed the potential of using NIR predictions to determine rice stem NSC (Fig. 5). From 434 rice stem samples selected to represent across the range of variation detected in the primary analytical data, we divided samples into either the calibration set or the external validation set using the Kennard–Stone algorithm. This resulted in 300 and 134 samples in the calibration and validation sets, respectively, which spanned the multi-dimensional spectral space (Supplementary Fig. S6). Two models were fitted for three primary NSC variables of interest: a PLS-1 model for TNC, and a PLS-2 model for both starch and sucrose. Overall, the models predicted TNC and starch with high accuracy, resulting in validation *R* ^2^ values of 0.92 and 0.96, respectively. The PLS-2 model yielded a validation *R* ^2^ of 0.76 for sucrose, with a satisfactory RMSEP of 0.02 that is comparable to the reference method uncertainty of 0.018 (see Methods for definitions). Final model results are summarized in Table 3 and Fig. 6. The average throughput for scanning on the Thermo-Antaris FT-NIR spectrometer using an autosampler carousel was approximately 40 samples/hour, with each sample being scanned 128 times. This estimate includes the time necessary for packing and cleaning vials and loading/unloading the scanning carousel. Our results support the use of NIR prediction models to determine NSC levels in rice stems, conditional on a reliable primary analytical method for calibration.

**Table 3.** Summary statistics for NIR calibration partial least-squares (PLS) models.

	PLS-1 model	PLS-2 model	
	TNC	Sucrose	Starch
Calibration samples	300	300	300
Independent validation samples	134	134	134
Outliers removed*	18	17	17
Principal components in model	8	10	10
*R* ^2^ calibration	0.96	0.86	0.96
*R* ^2^ cross validation	0.95	0.84	0.95
*R* ^2^ independent validation	0.92	0.74	0.96
RMSEC	0.02	0.015	0.015
RMSECV	0.02	0.016	0.016
RMSEP validation	0.025	0.02	0.013
Reference method uncertainty	0.024	0.018	0.022

* From calibration set.

RMSE: root mean square errors for calibration (C), cross-validation (CV), and prediction (P).

## Discussion

### Heading stem NSC as a potential breeding target for rice

The results of our field evaluation on the breeder’s panel suggest that the amount of stem carbohydrates accumulated by heading may be an appropriate target for rice varietal improvement. Post-heading stem reserve mobilization appears to be primarily a function of sink strength and environment/management (Yang *et al.*, 2000; Chen and Wang, 2008; Kim *et al.*, 2011; Morita and Nakano, 2011), while levels measured at maturity can be confounded by additional photoassimilate re-accumulation at the end of grain-filling. The extent to which rice accumulates, remobilizes, and re-accumulates stem NSC are each affected by genotype, environment, and genotype × environment interaction; however, earlier traits (e.g. accumulation measured at heading stage) are probably the most genetically tractable, as evidenced here by higher narrow-sense heritabilities with heading NSC traits. Additional field evaluation is necessary to validate the finding that performance of early-maturing rice varieties benefits from increased pre-heading stem carbohydrate accumulation.

In our single field study, grain yield was significantly associated with relative starch at heading (% w/w) across the panel. When evaluating Early, Medium, and Late maturity groups separately, we found that the linkages between stem NSC and yield traits (yield, harvest index, grain-filling percentage, and thousand-grain weight) were strongest for the Early maturity group while generally insignificant for the Medium and Late groups. These associations all involved NSC heading traits, except for grain-filling percentage that was positively correlated with stem starch at maturity. Since the level of stem NSC at maturity is a combination of NSC retained from heading and any end-of-season re-accumulation, this last outcome may be due to one or both of the following: (1) an indirect consequence of sink limitation (e.g. low number of total spikelets) that prematurely resulted in a high percentage of grains filled and a decrease in panicle sink strength that led to re-accumulation of stem NSCs; and (2) adequate photoassimilate production from flag leaves during grain-filling that reduced the need for pre-anthesis stem NSC. In this evaluation, there was no overall association of grain-filling percentage with grain yield, a possible demonstration of the diverse strategies (and therefore constraints) for yield formation taken by this collection of elite germplasm; some entries may be sink-limited in the panicle, while others may have experienced flag leaf source limitations. Additional trials over seasons and environments are necessary to determine the consistency of these results.

In addition to finding maturity group-level differences, we documented a curious contrast in NSC characteristics between BP 25 and BP 26, two accessions of cv. Teqing within the IRRI gene bank. Documentation suggests these accessions were contributed into the gene bank 17 years apart (IRRI International Rice Information System, http://irri.org/resources, accessed 30 September 2016) and represent similar but not genetically identical versions of the named variety, cv. Teqing, from China. In our yield trial BP 25 and BP 26 displayed similar yield (6.3 and 6.29 ton ha^–1^, respectively), harvest index (0.46 for both), and growth duration (130 d for both) but differed in phenology (flowering time and grain-filling duration) and stem NSC patterns. These two nearly-isogenic accessions are a valuable resource to help disentangle the physiological and genetic linkages between NSC and phenology.

Observed dependency of NSC–yield relationships on phenology in our work here on tropical rice is consistent with prior studies on temperate-adapted near-isogenic lines and with the hypothesis that carbohydrates accumulated prior to heading may be more critical for yield formation in plants with a shorter lifespan (Van Dat and Peterson, 1983). We rationalize that short-duration varieties do not have a long vegetative growth period to produce lavish amounts of leaf biomass that may later serve as source organs to generate photoassimilates concurrently with grain-filling. This may underlie the tight relationships between pre-heading stem reserves and yield attributes in the BP’s Early maturity group and raises the possibility of tailoring phenologically dependent source–sink relationships in rice breeding.

Previous studies have demonstrated physiological linkages within the triad of non-structural carbohydrate remobilization, monocarpic senescence, and the stay-green condition, with the former two acting oppositely to the latter. Environmental factors that hasten senescence and enhance NSC remobilization (e.g. water limitation and temperature increase) are also common climate change variables of concern, which highlights future potential of breeding for high accumulation of NSC at heading to buffer against increased within-season environmental variability. However, long-term expectations should be tempered until effects of other climatic parameters (e.g. rising atmospheric CO_2_) on pre-heading stem NSC are better understood, as they may have unexpected interaction effects (Moya *et al.*, 1998; Baker, 2004; Ziska *et al.*, 2014; Wang *et al.*, 2016).

### Evaluating the genetic control of rice stem NSC

From the results of our greenhouse evaluation, over half of the phenotypic variation found in rice stem NSC due to genetic factors was contributed by additive genetic variance. This suggests that direct selection for rice stem NSC is possible. Other strategies to optimize stem carbohydrate dynamics may include indirect selection via correlated traits or indices. These approaches require that target traits are not merely phenotypically correlated with stem NSC, but strongly genetically correlated. Genetic correlation arises due to pleiotropy or gametic phase disequilibrium, and this metric is often overlooked in crop physiology studies that focus on phenotypic correlation. Here, we found that stem starch and sucrose have positive genetic correlation, suggesting that it would be possible to select simultaneously for increased starch and sucrose. This may reflect the fact that starch synthesis in the rice stem depends on glucoses that result from sucrose breakdown, so a greater sucrose influx represents greater potential for starch accumulation. Using genetic correlations, we also found evidence that genotypes with few but heavy tillers tend to accumulate greater proportions of stem starch by the time they flower, suggesting that tiller number and weight have potential as indirect indicators of stem starch levels at heading.

Our simulation study indicates that five replicates are adequate for estimating line mean, GEBV, and variance components for rice stem NSC traits under greenhouse conditions. The mobile nature of stem NSC reserves, expressed as seasonal fluctuations in response to internal or external cues, gives rise to the idea that uncertainty measures of these traits are biologically relevant and may have a tractable genetic basis. Here, we show that twice as many experimental replicates are required to estimate uncertainty measures (e.g. line CV) as are sufficient to estimate line mean and GEBV. Despite this greater replication requirement (10 replicates), studying the genetic basis of line CV is still possible under conditions similar to those in which this experiment was carried out. Here, we assessed 36 genotypes replicated 20 times. Assuming the same planting constraints (pot size, spacing, etc), evaluating the 10 replicates necessary for line CV estimation would allow us to consider 72 genotypes, equivalent to a small or medium-sized bi-parental mapping population in rice.

While we were able to demonstrate here that stem NSC traits in rice are genetically tractable under controlled conditions, these results must be validated under multiple field environments if NSC-related traits are to prove applicable for selection purposes. Previous work on wheat has documented discrepancies between evaluation conditions, especially between field and controlled environments (Rebetzke *et al.*, 2013), and the importance of replication and validation cannot be stressed enough for breeding purposes. While direct selection cannot be performed under greenhouse environments, undertaking genetic studies under controlled conditions is a logical precursor to assess whether similar evaluations should be replicated under field conditions. In other words, if heritability is zero under controlled conditions, one can hardly hope for better results under much more heterogeneous field conditions.

### Applicability of NIRS for large-scale prediction of stem NSC

Given the time and labor-saving attributes of NIRS prediction for grass biomass constituents relative to traditional analytical chemistry, the reason underlying the apparent lag of NIRS application for predicting rice stem NSC is unclear. One explanation may be that multidisciplinary expertise is needed for optimal model development. Lack of domain knowledge in either NIR spectroscopy or in the application area may pose limitations on the long-term success of using NIRS models for prediction. Here, we show that NIRS calibration models can accurately predict rice stem NSC constituents across genetically and experimentally diverse samples. To our knowledge, only one study has been published that has assessed the utility of NIRS for rice stem NSC prediction, using 61 rice stem samples for starch only (Batten *et al.*, 1993). An *R* ^2^ of 0.98 and a standard error of performance of 1.5 (% starch) were reported; however, it appears that these values are likely results from cross-validation. Prediction of an unknown set of samples is critically dependent upon adequate representation of the calibration set across the spectral space of the prediction samples. To that end, for our calibration we made sure to select samples as diverse as possible, choosing across wet chemistry values, sampling points (heading versus maturity), genetic identity, and experimental conditions (two experiments across two years). With that, we expect we can predict rice stem NSC composition in future experiments under similar greenhouse conditions for large-scale genetic studies. Research avenues to further increase efficiency of rice stem NSC phenotyping may include improving NIR prediction by tailoring calibration sets to specific prediction samples using local models (as in Godin *et al.*, 2015) or implementing non-destructive, in-field methods of NSC determination such as hyperspectral reflectance, as demonstrated in wheat (Dreccer *et al.*, 2014).

The possibility of optimizing stem NSC dynamics for rice varietal improvement, an idea whose roots go back several decades, grows more relevant with rising concerns about the impacts of climate variability. As has been demonstrated previously (Yang *et al.*, 2002; Kim *et al.*, 2011; Morita and Nakano, 2011), post-heading temperature and water availability stresses shift rice dependencies heavily towards pre-heading carbohydrate reserves. Understanding the genetic architecture underlying stem NSC accumulation, remobilization, and re-accumulation may be key to its utility, not only for rice but also for other economically important grass species.

## Supplementary data

Supplementary data are available at *JXB* online.

Table S1. Phenotype and germplasm information for the breeder’s panel.

Table S2. Germplasm information for the diversity panel.

Table S3. Trait measurement methodology in the breeder’s panel.

Figure S1. Hierarchichal clustering of the breeder’s panel using NSC traits.

Figure S2. NSC distribution by senescence class.

Figure S3. Distribution of NSC traits by sampling point for six selected BP entries in the IRRI field trial and the greenhouse diversity screen.

Figure S4. Accession-specific distributions of NSC traits ordered by decreasing line mean.

Figure S5. Genotyping results of six selected breeding lines, including genome distribution of 6125 SNPs and results from principal components analysis.

Figure S6. Comparison of calibration and validation sets for NIR calibration.

Dataset S1. Full description of Bayesian analyses for the experimental design study.

Dataset S2. Zip file containing scripts and related files for the Bayesian analyses.

Supplementary Data
