# Supplementary material for: Robust phenotyping strategies for evaluation of stem non-structural carbohydrates (NSC) in rice
Source: J Exp Bot. 2016 Oct 5;67(21):6125–38. doi: 10.1093/jxb/erw375 (PMC5100024; doi:10.1093/jxb/erw375)
Supplement: Supplementary Data [file supp_67_21_6125__index.html]

Robust phenotyping strategies for evaluation of stem non-structural carbohydrates (NSC) in rice — Robust phenotyping strategies for evaluation of stem non-structural carbohydrates (NSC) in rice — Supplementary Data 

# Robust phenotyping strategies for evaluation of stem non-structural carbohydrates (NSC) in rice

## Supplementary Data

Data files

- supplementary\_figures\_S1\_S6.pdf - Supplementary Data
- Supplementary\_tables\_S1\_S3.xlsx - Supplementary Data
- supplementary\_dataset\_S1.pdf - Supplementary Data
- supplementary\_dataset\_S2.zip - Supplementary Data
