# Supplementary material for: Robust phenotyping strategies for evaluation of stem non-structural carbohydrates (NSC) in rice
Source: J Exp Bot. 2016 Oct 5;67(21):6125–38. doi: 10.1093/jxb/erw375 (PMC5100024; doi:10.1093/jxb/erw375)

Robust phenotyping strategies for evaluation of stem non-structural carbohydrates (NSC) in rice

Supplemental Figures S1-S6

Wang et al. 2016

Supplementary Fig. S1. Hierarchical clustering analysis of breeder's panel using NSC traits. Results of hierarchical clustering using average linkage on scaled line means of NSC trait data on 33 breeding lines. Names are colored as in **Fig. 3**. Additional annotations indicate meta-information: yellow ovals are individuals selected for further evaluation with diverse germplasm, purple asterisks indicate entries with *tropical japonica* pedigree, and brown crosses refer to breeding lines (versus released variety).

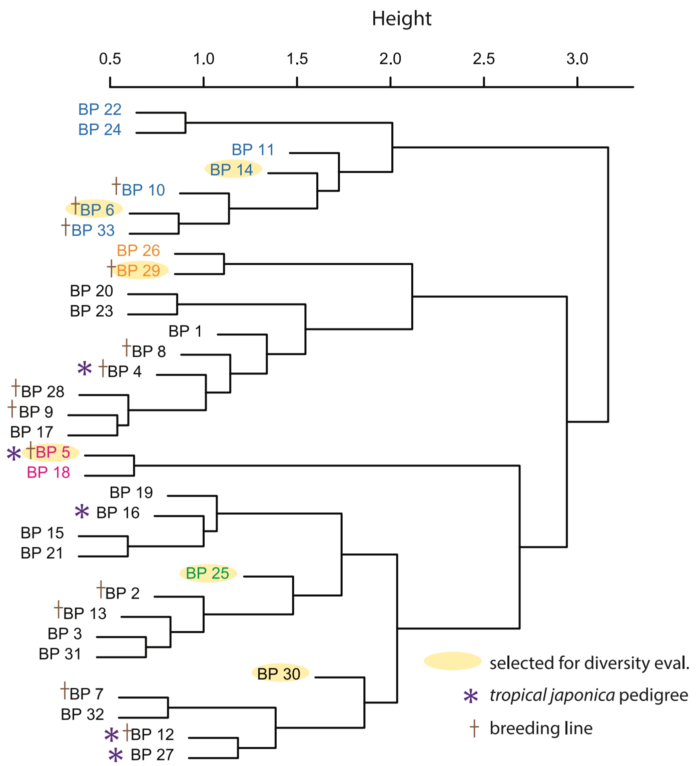

Supplementary Fig. S2. NSC distribution by senescence class

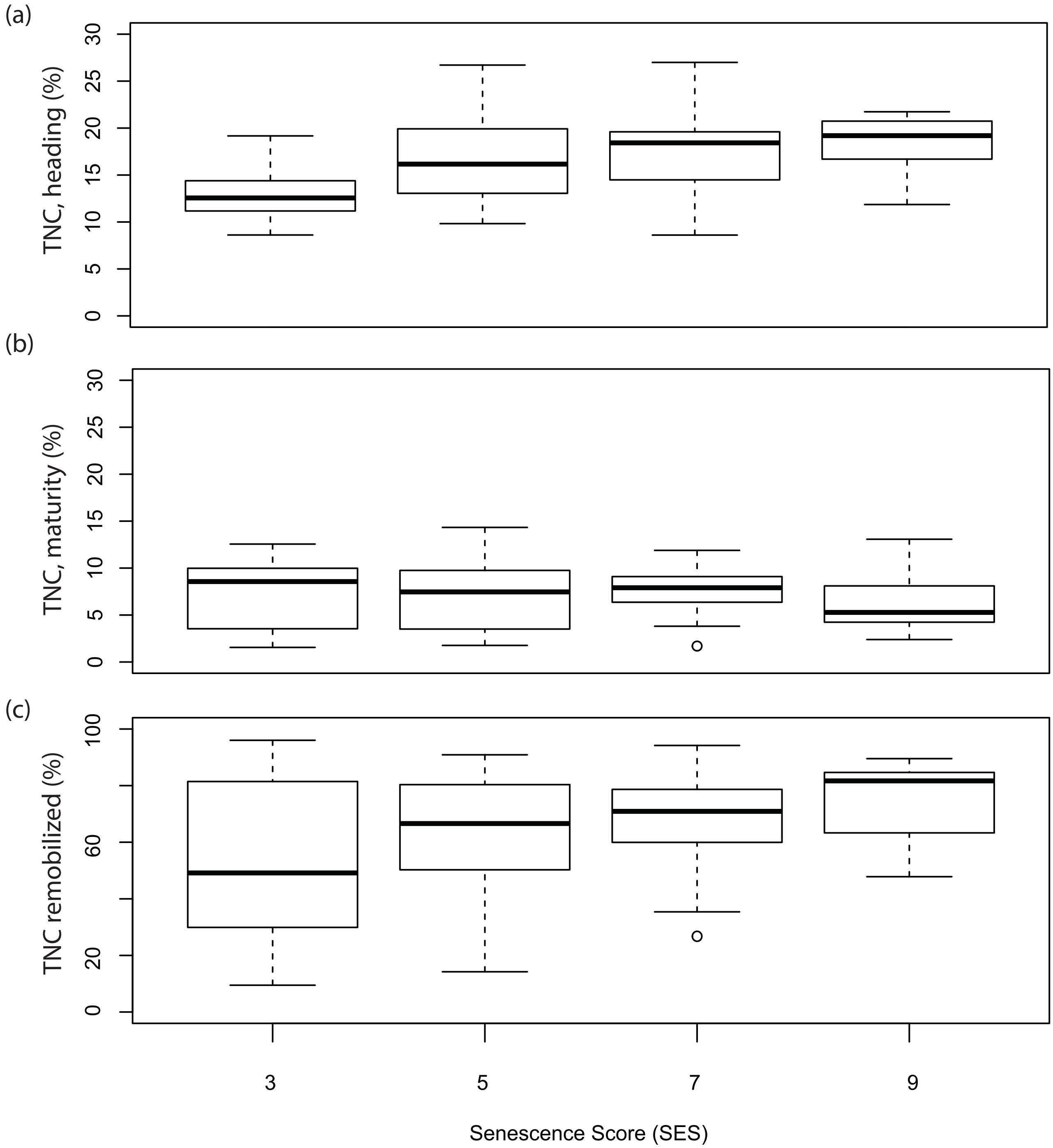

Supplementary Fig. S3. Distribution of NSC traits by sampling point for 6 selected BP entries in the IRRI field trial (a-c) and greenhouse diversity screen (d-f)

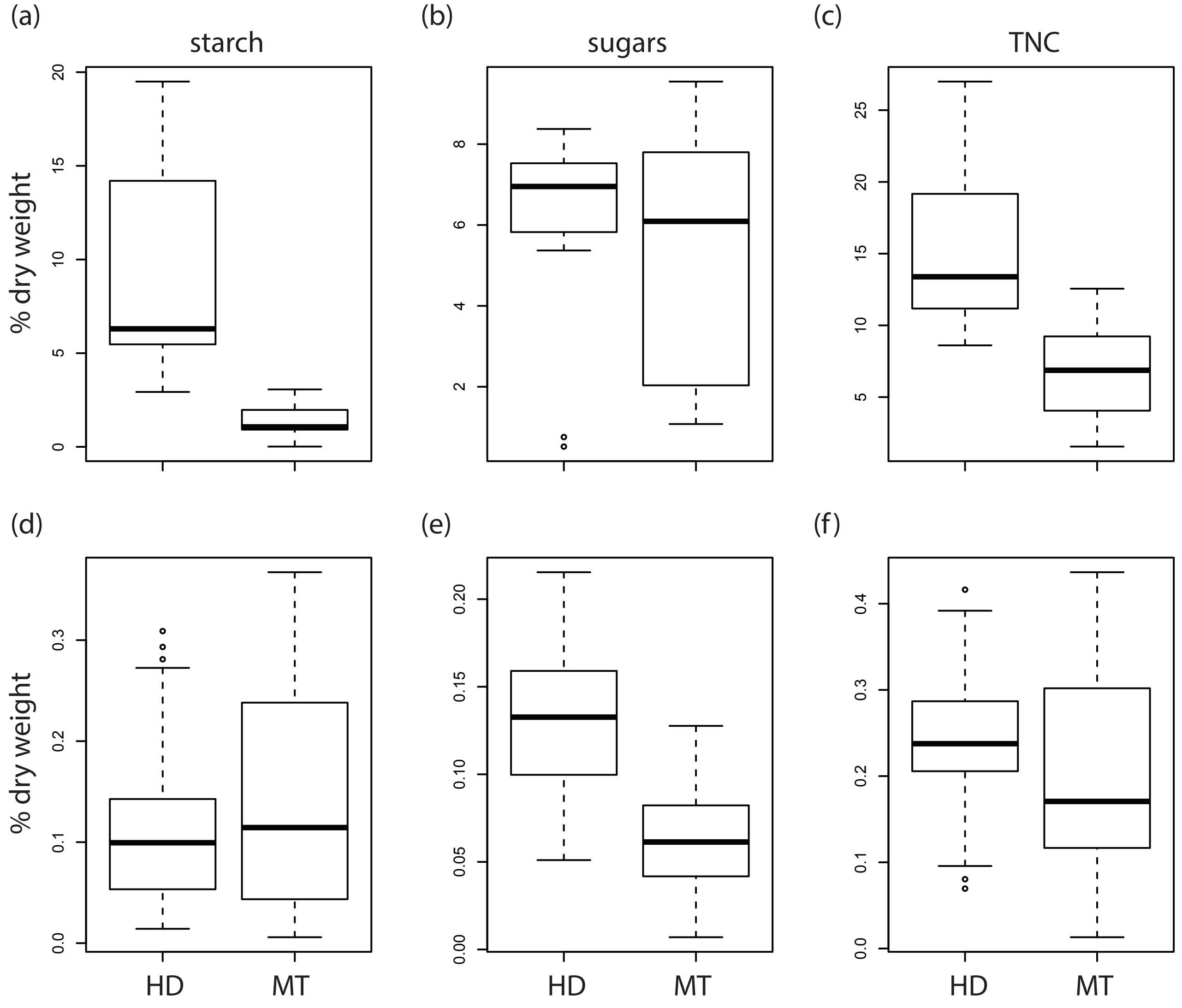

Supplementary Fig. S4. Accession-specific distributions of NSC traits ordered by decreasing line mean.

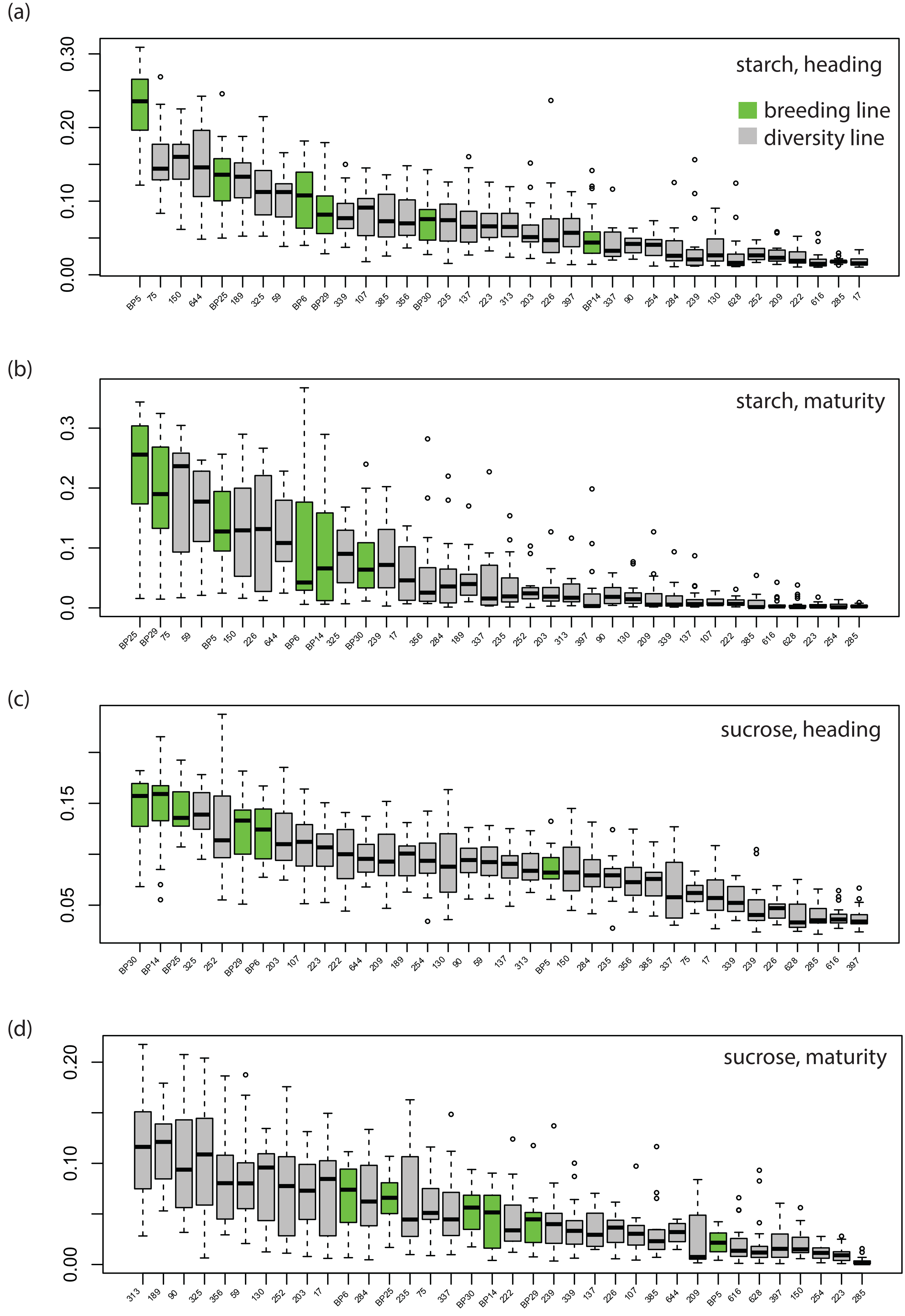

Supplementary Fig. S5. GBS results of 6 selected BP entries and 54 *O. sativa* population controls

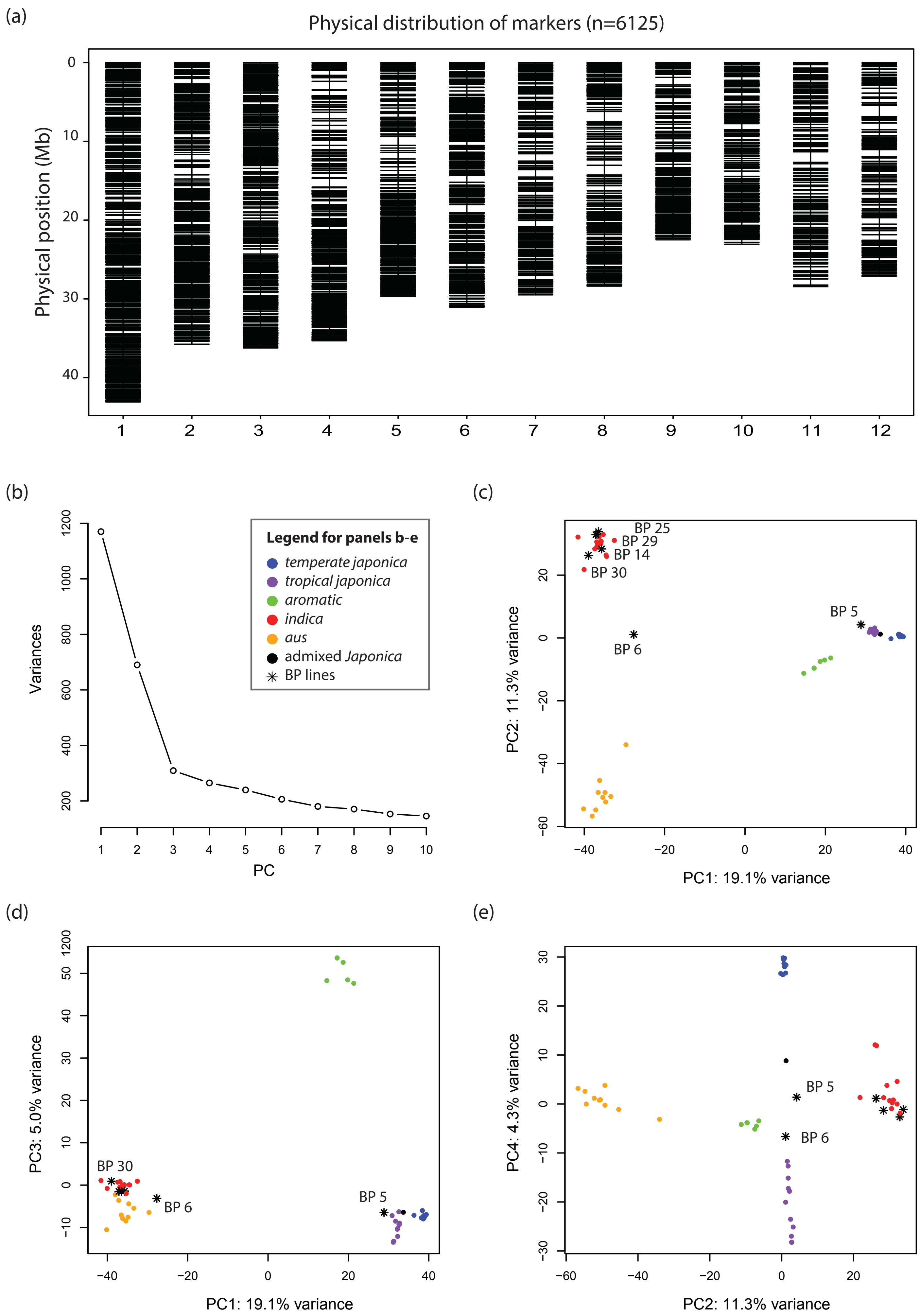

Supplementary Fig. S6. PCA of spectral data on 434 samples used in calibration and validation of NIR models.

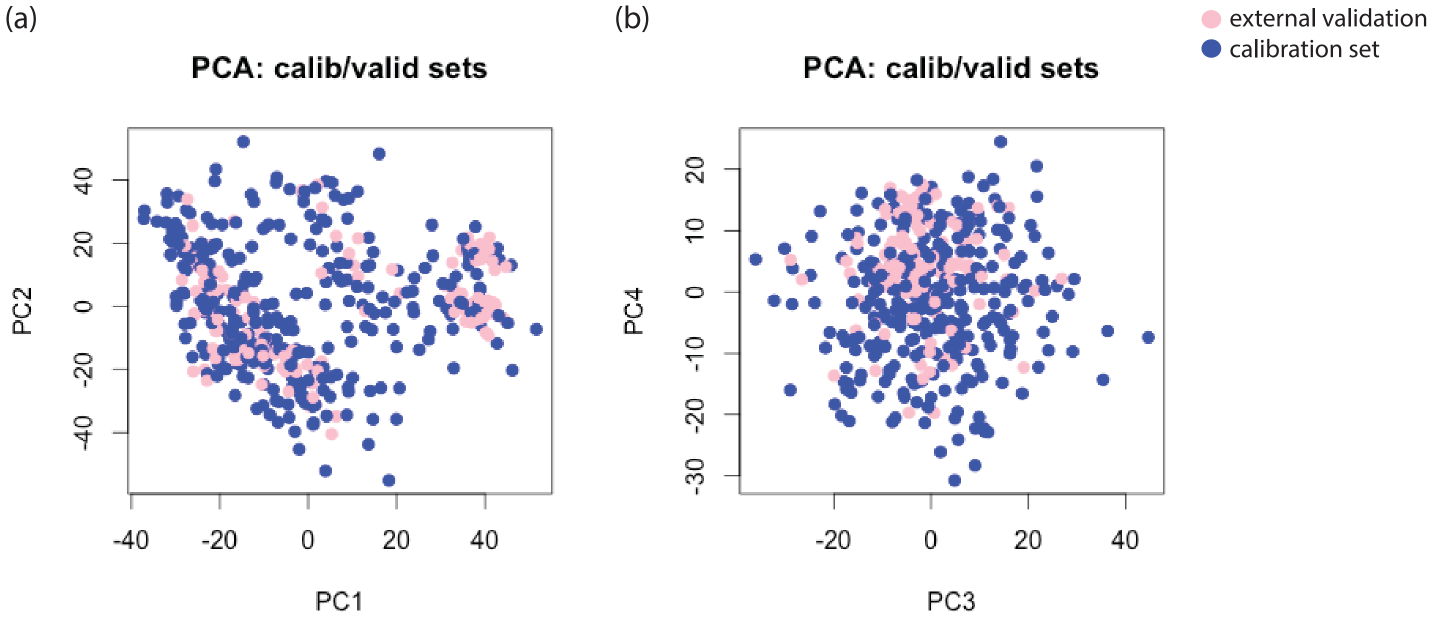

Supplement: Supplementary Data [file supp_erw375_supplementary_figures_S1_S6.pdf]
