# Supplementary material for: Robust phenotyping strategies for evaluation of stem non-structural carbohydrates (NSC) in rice
Source: J Exp Bot. 2016 Oct 5;67(21):6125–38. doi: 10.1093/jxb/erw375 (PMC5100024; doi:10.1093/jxb/erw375)
Supplement: Supplementary Data [file supp_erw375_supplementary_dataset_S1.pdf]

Full Description of Data Preparation and Analysis

Anthony J. Greenberg

May 12, 2016

```
               -
platform       x86_64-apple-darwin15.0.0
version.string R version 3.2.2 (2015-08-14)
```

This document details the Bayesian data analysis pipeline for the highly replicated greenhouse non-structural hydrocarbon (NHC) variation data set.

1 Data preparation

Read in the trait data.

```
> nscDat <- read.table(file = "hiRepDataNew.csv", sep = "\t", header = T)
> dim(nscDat)
```

```
[1] 620  14
```

```
> summary(nscDat)
```

```
     Indiv            Genotype        Rep        GROUP          DTH       
 107.1  :  1   C9000JNY.C05: 20   R1     : 29   IND :380   Min.   : 57.0  
 107.10 :  1   C9000JNY.H04: 20   R10    : 29   IRRI: 40   1st Qu.: 76.0  
 107.11 :  1   NSFTV107    : 20   R11    : 29   TRJ :200   Median : 80.0  
 107.12 :  1   NSFTV130    : 20   R12    : 29              Mean   : 80.6  
 107.13 :  1   NSFTV137    : 20   R13    : 29              3rd Qu.: 85.0  
 107.14 :  1   NSFTV150    : 20   R14    : 29              Max.   :106.0  
 (Other):614   (Other)     :500   (Other):446              NA's   :47     
    STCH_HD            SUC_HD             SEN             TIL              WT        
 Min.   :0.01032   Min.   :0.02150   Min.   : 1.00   Min.   : 1.00   Min.   : 0.260  
 1st Qu.:0.02555   1st Qu.:0.05357   1st Qu.: 3.00   1st Qu.:11.00   1st Qu.: 1.550  
 Median :0.05628   Median :0.08024   Median : 5.00   Median :14.00   Median : 2.600  
 Mean   :0.06874   Mean   :0.08274   Mean   : 4.48   Mean   :14.96   Mean   : 2.925  
 3rd Qu.:0.10087   3rd Qu.:0.10596   3rd Qu.: 5.00   3rd Qu.:18.00   3rd Qu.: 3.615  
 Max.   :0.26882   Max.   :0.23743   Max.   :12.00   Max.   :47.00   Max.   :11.870  
 NA's   :44        NA's   :44        NA's   :43      NA's   :51      NA's   :41      
     GLC_HV            SUC_HV            STCH_HV              HDRA.id   
 Min.   :0.00120   Min.   :0.00017   Min.   :0.00029   09ad7034.0: 20  
 1st Qu.:0.00379   1st Qu.:0.01759   1st Qu.:0.00542   128dd425.0: 20  
```

```
 Median :0.00707   Median :0.04211   Median :0.02096   13ae4f27.0: 20
 Mean   :0.00896   Mean   :0.05439   Mean   :0.05723   298c9652.0: 20
 3rd Qu.:0.01192   3rd Qu.:0.08331   3rd Qu.:0.07979   3995b6ff.0: 20
 Max.   :0.06222   Max.   :0.21754   Max.   :0.36725   39afe586.0: 20
 NA's   :40        NA's   :40        NA's   :40        (Other)   :500
```

Make a vector of traits and see if there are any rows of the data matrix with all trait data missing.

```
> trt.list <- c("DTH", "STCH_HD", "SUC_HD", "SEN",
+         "TIL", "WT", "GLC_HV", "SUC_HV", "STCH_HV")
> naNind <- apply(nscDat[, trt.list], 1, function(vec){sum(!is.na(vec))})
> sum(naNind == 0)
```

```
[1] 25
```

There are 25 such rows. Eliminate them. The genotype factor may have changed, and in any case I do not want it to be sorted, so I re-apply the `factor()` function.

```
> nscDat <- nscDat[naNind != 0,]
> nscDat$Genotype <- factor(nscDat$Genotype, levels = unique(nscDat$Genotype))
> dim(nscDat)
```

```
[1] 595  14
```

```
> summary(nscDat)
```

```
     Indiv          Genotype         Rep        GROUP          DTH            STCH_HD
 107.1   :  1   NSFTV17 : 20   R1      : 29   IND :374   Min.   : 57.0   Min.   :0.01032
 107.10  :  1   NSFTV59 : 20   R15     : 29   IRRI: 36   1st Qu.: 76.0   1st Qu.:0.02555
 107.15  :  1   NSFTV90 : 20   R20     : 29   TRJ :185   Median : 80.0   Median :0.05628
 107.18  :  1   NSFTV130: 20   R3      : 29              Mean   : 80.6   Mean   :0.06874
 107.19  :  1   NSFTV137: 20   R6      : 29              3rd Qu.: 85.0   3rd Qu.:0.10087
 107.20  :  1   NSFTV150: 20   R7      : 29              Max.   :106.0   Max.   :0.26882
 (Other):589   (Other) :475   (Other):421              NA's   :22      NA's   :19
     SUC_HD             SEN              TIL              WT              GLC_HV
 Min.   :0.02150   Min.   : 1.00   Min.   : 1.00   Min.   : 0.260   Min.   :0.001203
 1st Qu.:0.05357   1st Qu.: 3.00   1st Qu.:11.00   1st Qu.: 1.550   1st Qu.:0.003794
 Median :0.08024   Median : 5.00   Median :14.00   Median : 2.600   Median :0.007071
 Mean   :0.08274   Mean   : 4.48   Mean   :14.96   Mean   : 2.925   Mean   :0.008960
 3rd Qu.:0.10596   3rd Qu.: 5.00   3rd Qu.:18.00   3rd Qu.: 3.615   3rd Qu.:0.011921
 Max.   :0.23743   Max.   :12.00   Max.   :47.00   Max.   :11.870   Max.   :0.062224
 NA's   :19        NA's   :18      NA's   :26      NA's   :16       NA's   :15
     SUC_HV              STCH_HV                HDRA.id
 Min.   :0.000173   Min.   :0.000290   128dd425.0: 20
 1st Qu.:0.017590   1st Qu.:0.005418   13ae4f27.0: 20
 Median :0.042108   Median :0.020958   298c9652.0: 20
 Mean   :0.054388   Mean   :0.057234   3995b6ff.0: 20
 3rd Qu.:0.083310   3rd Qu.:0.079793   39afe586.0: 20
 Max.   :0.217544   Max.   :0.367247   446f6c62.0: 20
 NA's   :15         NA's   :15         (Other)   :475
```

The genotypes are in the file `HDRAdiane.tped`, which has SNPs filtered to eliminate singletons and variants with greater than 30% missingness. The file is coded with (1,2) SNP coding.

Plot histograms of the traits.

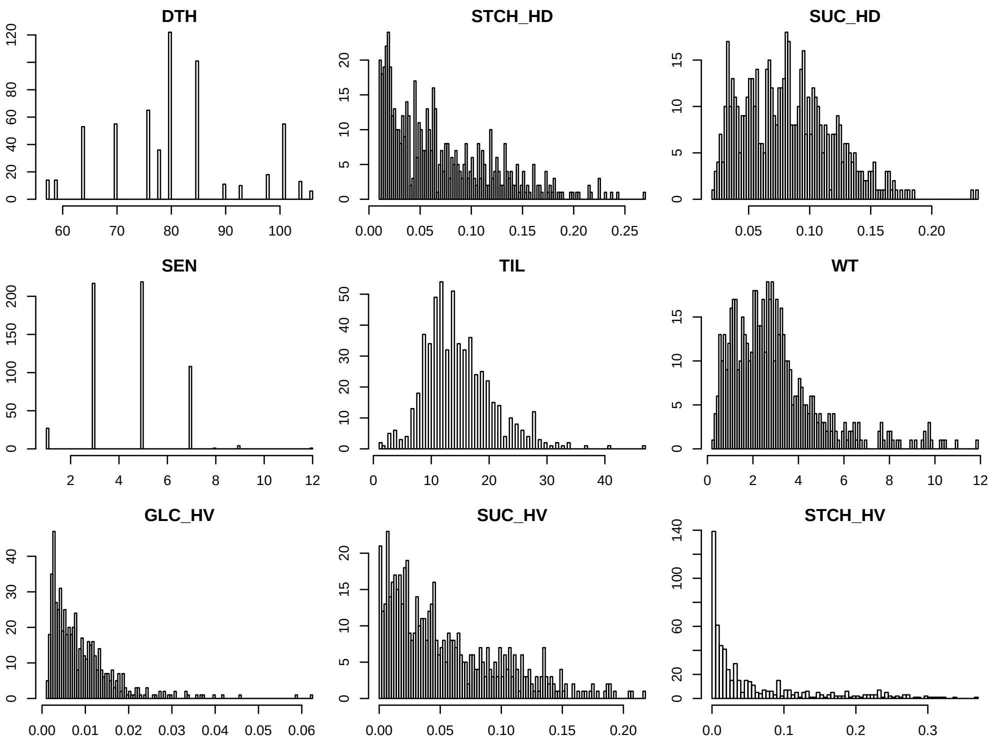

I read in the SNP file and modify the resulting table to introduce `NA`s for missing data, and convert to individual genotypes from two values for a diploid individual, as is standard in `plink`.

```
> snpFl <- pipe("cut -d ' ' -f5- HDRAdiane.tped")
> SNP.HDRA <- matrix(scan(snpFl, what = integer()),
+         ncol = 2*nlevels(nscDat$HDRA.id), byrow = T)
> close(snpFl)
> dim(SNP.HDRA)
```

```
[1] 347558     62
```

```
> SNP.HDRA[SNP.HDRA == 0] <- NA
> SNP.HDRA <- SNP.HDRA - 1
> SNP.HDRA <- (SNP.HDRA[,seq(from = 1, to = dim(SNP.HDRA)[2] - 1, by = 2)]
+         + SNP.HDRA[,seq(from = 2, to = dim(SNP.HDRA)[2], by = 2)])
```

No transpose the SNP table to bring to the format compatible with my `MuGen` library.

```
> SNP.HDRA <- t(SNP.HDRA)
> dim(SNP.HDRA)
```

```
[1]     31 347558
```

Next, I read the HDRA IDs for the master data set.

```
> pedIndFl <- pipe("cut -d ' ' -f1 HDRAdiane.tfam")
> pedInd   <- scan(pedIndFl, what = character())
> close(pedIndFl)
> rownames(SNP.HDRA) <- as.character(pedInd)
>
```

Now I re-arrange the rows of SNP.HDRA to match the order in the trait data set.

```
> SNP.HDRA <- SNP.HDRA[unique(as.character(nscDat$HDRA.id)),]
> dim(SNP.HDRA)
```

```
[1]     31 347558
```

Finally, I re-factor the allele states to the (-1, 0, 1) scale from the (0, 1, 2) scale. This is required by the rrBLUP function that calculates relationship matrices.

```
> SNP.HDRA <- SNP.HDRA - 1
```

I now proceed to calculate the relationship matrix using the rrBLUP package.

```
> library(rrBLUP)
> K       <- A.mat(SNP.HDRA, impute.method="EM")
```

```
[1] "A.mat converging:"
[1] 0.0335
[1] 0.00154
```

```
> Keig    <- eigen(K)
> round(Keig$val, 5)
```

```
 [1] 22.21155  4.02108  2.27783  2.07048  1.87564  1.82741  1.69269  1.66208  1.54071
[10]  1.51900  1.42121  1.39022  1.36398  1.29504  1.17005  1.15536  1.13178  1.08287
[19]  1.05482  1.00375  0.92343  0.85770  0.83029  0.76640  0.73824  0.60093  0.56440
[28]  0.54995  0.35689  0.23666  0.00000
```

```
> nscEVL <- Keig$val[Keig$val > 1e-8]
> nscEVC <- Keig$vec[,Keig$val > 1e-8]
```

Save the eigenvalue vector and the eigenvector and SNP matrices in a format readable by MuGen-based software.

```
> SNP.HDRA[is.na(SNP.HDRA)] <- -9
> trash <- .C("GSLmatSave", "snpHDRAnsc.gbin",
+          as.double(SNP.HDRA), nrow(SNP.HDRA), ncol(SNP.HDRA))
> trash <- .C("GSLvecSave", "nscHDRA_EVL.gbin", as.double(nscEVL), length(nscEVL))
> trash <- .C("GSLmatSave", "nscHDRA_EVC.gbin",
+          as.double(nscEVC), nrow(nscEVC), ncol(nscEVC))
>
```

Now I proceed to sub-sampling the trait data. There are (mostly) 20 replicates for each accession. I create 20 data sets each with 2, 5, 10 and 15 replicates. For the cases where the number of replicates is fewer than the full 20, I keep up to the sub-sampling number. First, I writhe functions that will do the sub-sampling.

```
> rsmp <- cmpfun(function(vec, n){
+         if(length(vec) <= n) {return(vec)}
+         else {return(sample(vec, n))}
+ })
> resmpRow <- cmpfun(function(n){
+         nscDat[unlist(tapply(1:nrow(nscDat), nscDat$Genotype, rsmp, n)),]
+ })
```

The `resmpRow()` function runs `rsmp()` on each accession, sampling the necessary number of replicates.

```
> samp2  <- replicate(20, resmpRow(2), simplify = F)
> samp5  <- replicate(20, resmpRow(5), simplify = F)
> samp10 <- replicate(20, resmpRow(10), simplify = F)
> samp15 <- replicate(20, resmpRow(15), simplify = F)
> nrow(samp2[[1]])
```

```
[1] 62
```

```
> nrow(samp5[[1]])
```

```
[1] 155
```

```
> nrow(samp10[[1]])
```

```
[1] 310
```

```
> nrow(samp15[[1]])
```

```
[1] 462
```

```
> nrow(nscDat)
```

```
[1] 595
```

Finally, I save the data sets in the `MuGen`-readable format.

```
> saveMats <- cmpfun(function(i, lst, nam){
+         dmat <- as.matrix(lst[[i]][,trt.list])
+         matNAind <- apply(dmat, 2, function(vec){ifelse(is.na(vec), 1, 0)})
+         totNAind <- rowSums(matNAind)
+         dmat[is.na(dmat)] <- -10
+         bin <- .C("GSLvecSaveInt", paste("subSampledData/hiRepMatNAind_",
+                         nam, "_", i, ".gbin", sep = ""),
+                 as.integer(t(matNAind)), as.integer(prod(dim(matNAind))))
+         bin <- .C("GSLvecSaveInt", paste("subSampledData/hiRepTotNAind_",
+                         nam, "_", i, ".gbin", sep = ""),
+                 as.integer(totNAind), length(totNAind))
+         bin <- .C("GSLvecSaveInt", paste("subSampledData/hiRepLnInd_",
+                         nam, "_", i, ".gbin", sep = ""),
+                 as.integer(lst[[i]][,2]), nrow(lst[[i]]))
+         bin <- .C("GSLmatSave", paste("subSampledData/hiRepData_",
+                         nam, "_", i, ".gbin", sep = ""),
+                 as.double(dmat), nrow(dmat), ncol(dmat))
+         return(NULL)
+ })
> trash <- sapply(1:20, saveMats, samp2, 2)
> trash <- sapply(1:20, saveMats, samp5, 5)
> trash <- sapply(1:20, saveMats, samp10, 10)
> trash <- sapply(1:20, saveMats, samp15, 15)
> dmat <- as.matrix(nscDat[,trt.list])
> matNAind <- apply(dmat, 2, function(vec){ifelse(is.na(vec), 1, 0)})
> totNAind <- rowSums(matNAind)
> dmat[is.na(dmat)] <- -10
> trash <- .C("GSLvecSaveInt",
+         paste("subSampledData/hiRepMatNAind_20_1.gbin", sep = ""),
+         as.integer(t(matNAind)), as.integer(prod(dim(matNAind))))
> trash <- .C("GSLvecSaveInt",
+         paste("subSampledData/hiRepTotNAind_20_1.gbin", sep = ""),
+         as.integer(totNAind), length(totNAind))
> trash <- .C("GSLvecSaveInt",
+         paste("subSampledData/hiRepLnInd_20_1.gbin", sep = ""),
+         as.integer(nscDat[,2]), nrow(nscDat))
> trash <- .C("GSLmatSave",
+         paste("subSampledData/hiRepData_20_1.gbin", sep = ""),
+         as.double(dmat), nrow(dmat), ncol(dmat))
>
```

I list all dimensions in one place for easy access:

```
> dim(nscDat)

[1] 595  14
```

```
> dim(SNP.HDRA)
[1]     31 347558

> nlevels(nscDat$Genotype)
[1] 31

> nrow(samp2[[1]])
[1] 62

> nrow(samp5[[1]])
[1] 155

> nrow(samp10[[1]])
[1] 310

> nrow(samp15[[1]])
[1] 462

> dim(dmat)
[1] 595   9
```

2 MCMC chain processing

The model to analyze the data is a simple hierarchical one.

$$\begin{aligned}
\boldsymbol{y}_{i\cdot} &\sim t_{\nu,d}\left(\boldsymbol{\mu}^{ln}_{j[i]\cdot};\ \boldsymbol{\Sigma}_e\right)\\
\boldsymbol{\Sigma}_e^{-1} &\sim \mathrm{W}_d\left(\left[\sum_i\left(\boldsymbol{y}_{i\cdot}-\boldsymbol{\mu}^{ln}_{j[i]\cdot}\right)^{\mathrm{T}}\left(\boldsymbol{y}_{i\cdot}-\boldsymbol{\mu}^{ln}_{j[i]\cdot}\right)\right]^{-1}\right)\\
\boldsymbol{\mu}^{ln}_{j\cdot} &\sim \mathrm{N}_d\left(\boldsymbol{\mu}+\mathbf{u}_{j\cdot}\boldsymbol{\Gamma};\ \boldsymbol{\Sigma}_s\right)\\
\boldsymbol{\Sigma}_s^{-1} &\sim \mathrm{W}_d\left(\left[\left(\boldsymbol{M}^{ln}-\boldsymbol{\mu}-\boldsymbol{U}\boldsymbol{\Gamma}\right)^{\mathrm{T}}\left(\boldsymbol{M}^{ln}-\boldsymbol{\mu}-\boldsymbol{U}\boldsymbol{\Gamma}\right)\right]^{-1}\right)\\
\boldsymbol{\gamma}_{l\cdot} &\sim \mathrm{N}_d\left(\mathbf{0};\ \boldsymbol{\Sigma}_a\right)\\
\boldsymbol{\Sigma}_a^{-1} &\sim \mathrm{W}_d\left(\left[\boldsymbol{\Gamma}^{\mathrm{T}}\boldsymbol{\Gamma}\right]^{-1}\right)\\
\boldsymbol{\mu} &\sim \mathrm{N}_d\left(\mathbf{0};\ \boldsymbol{\Sigma}_{pr}\right)
\end{aligned}$$

All location parameter matrices have the d traits in columns with replicates, lines, etc. as rows. The notation of the type $\boldsymbol{y}_{i\cdot}$ denotes a row of such a matrix. The matrix itself would be written $\boldsymbol{Y}$. Following common hierarchical model notation, $j[i]$ is the j-th high-level (in the model hierarchy) row that corresponds to the i-th row in the lower-level matrix. $\boldsymbol{\Sigma}_{\text{x}}$ are variance-covariance matrices. Among them, $\boldsymbol{\Sigma}_{pr}$ is the covariance matrix used for vague zero-mean priors, and is the d-dimensional diagonal matrix with a large value (in our case, 10^6) on the diagonal. The $\boldsymbol{\Gamma}$ matrix contains regression coefficients of the principal components of the genetic relationship matrix $\boldsymbol{K}$, multiplied by the squared-root of the corresponding eigenvalues; $\mathbf{u}_{j\cdot}$ is the j-th row of this scaled-PC matrix $\boldsymbol{U}$; it corresponds to the j-th accession. ν is the degrees freedom of the Student-t distributions for errors. They are set ahead of time. I am presenting results for the Gaussian (which is Student-t with $\nu \to \infty$, and is a achieved in practice by setting $\nu = 1000$) and Student-t with $\nu = 3$, which is the smallest value that still results in defined covariance and mean of the distribution. The distributions are $\text{N}_d()$ for multivariate Gaussian, $t_{\nu,d}()$for multivariate Student-t and W_d for Wieshart.

I ran the analyses using the `hiRep.cpp` program (included in the folder with this document), that depends on the `MuGen` library. The library in turn depends on the GNU Scientific Library. `MuGen` source and compilation instructions can be obtained from `https://github.com/tonymugen/MuGen`; documentation of the interface can be found at `http://www.bayesicresearch.org/mugen/`. I used a `bash` script, `runHiRep.sh`, to run `hiRep` on all the sub-sampled data sets.

2.1 Model with Gaussian errors

I first load the results from the full data set, after defining some constants. I start with line (accession) means.

```
> d      <- 9
> Nln    <- 31
> lnDim  <- d*Nln
> nChn   <- 5
> chnLen <- 2000
> chn1 <- matrix(.C("GSLmatLoad",
+         "NSCchains1000/LN_20_1_1000_1.gbin",
+         as.integer(chnLen), as.integer(lnDim), out = double(chnLen*lnDim))$out,
+                nrow = chnLen, byrow = T)
> chn2 <- matrix(.C("GSLmatLoad",
+         "NSCchains1000/LN_20_1_1000_2.gbin",
+         as.integer(chnLen), as.integer(lnDim), out = double(chnLen*lnDim))$out,
+                nrow = chnLen, byrow = T)
> chn3 <- matrix(.C("GSLmatLoad",
+         "NSCchains1000/LN_20_1_1000_3.gbin",
+         as.integer(chnLen), as.integer(lnDim), out = double(chnLen*lnDim))$out,
+                nrow = chnLen, byrow = T)
> chn4 <- matrix(.C("GSLmatLoad",
+         "NSCchains1000/LN_20_1_1000_4.gbin",
+         as.integer(chnLen), as.integer(lnDim), out = double(chnLen*lnDim))$out,
+                nrow = chnLen, byrow = T)
```

```
> chn5 <- matrix(.C("GSLmatLoad",
+         "NSCchains1000/LN_20_1_1000_5.gbin",
+         as.integer(chnLen), as.integer(lnDim), out = double(chnLen*lnDim))$out,
+                 nrow = chnLen, byrow = T)
>
```

Now I plot the Gelman-Rubin convergence statistics for each trait. The function to calculate statistic follows.

```
> gelRub <- cmpfun(function(vrNam, nChn, chnLen){
+         mat <- NULL
+         for (i in 1:nChn){
+                 mat <- rbind(mat, eval(as.symbol(paste(vrNam, i, sep=""))))
+         }
+         chnFac <- factor(rep(1:nChn, each=chnLen))
+         W <- colMeans(apply(mat, 2, tapply, chnFac, var))
+         B <- apply(apply(mat, 2, tapply, chnFac, mean), 2, var)
+         return( sqrt((((chnLen - 1)/chnLen)*W + B)/W) )
+ })
```

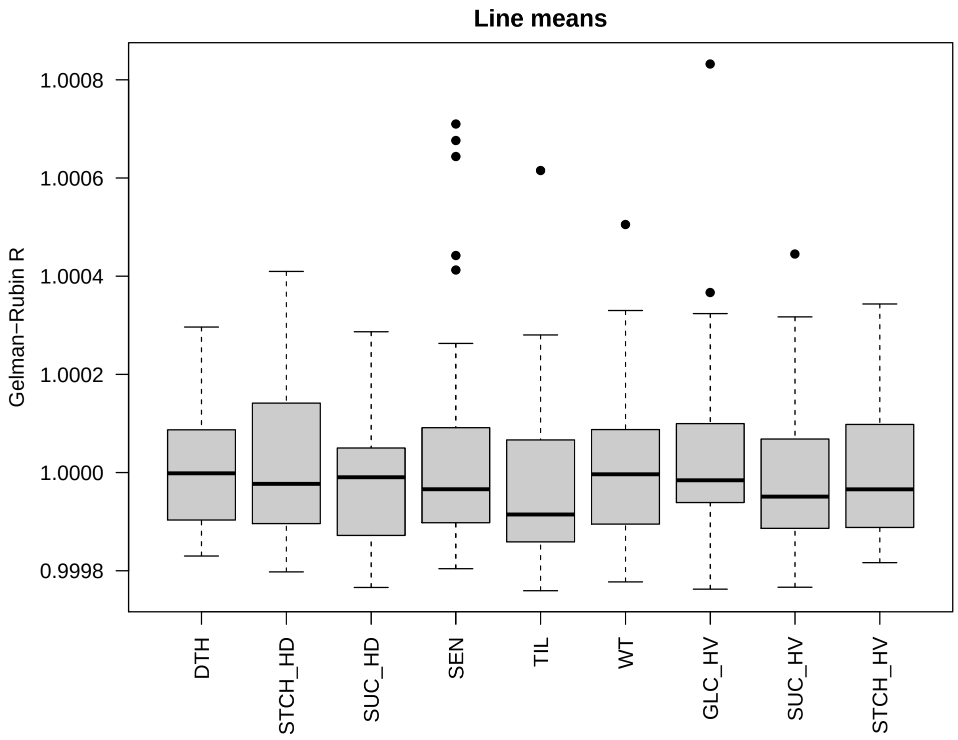

Values smaller than 1.2 reflect complete convergence. To calculate point estimates, I use the posterior mode. I define a function, `pmode(vec)`, that uses the standard `density()` function to find the mode (the function name `mode()` is already taken).

```
> pmode <- cmpfun(function(vec){
+         dst <- density(vec, adjust = 2)
```

```
+         mxi <- which(dst$y == max(dst$y))
+         if(length(mxi) > 1){
+                 warning("More than one mode in call to pmode(); picking randomly")
+                 mxi <- sample(mxi, 1)
+         }
+         dst$x[mxi]
+ })
```

I proceed to create a matrix of line mean point estimates, and a matrix of their MCMC relative errors.

```
> tmpMat       <- rbind(chn1, chn2, chn3, chn4, chn5)
> fullLNmatN   <- matrix(apply(tmpMat, 2, pmode), ncol = d, byrow = T)
> fullLNmatNcv <- matrix(apply(tmpMat, 2, sd), ncol = d, byrow = T)/abs(fullLNmatN)
> colnames(fullLNmatN)   <- trt.list
> colnames(fullLNmatNcv) <- trt.list
```

Next, I move to genome-estimated breeding values (GEBV).

```
> chn1 <- matrix(.C("GSLmatLoad",
+         "NSCchains1000/BV_20_1_1000_1.gbin",
+         as.integer(chnLen), as.integer(lnDim), out = double(chnLen*lnDim))$out,
+                 nrow = chnLen, byrow = T)
> chn2 <- matrix(.C("GSLmatLoad",
+         "NSCchains1000/BV_20_1_1000_2.gbin",
+         as.integer(chnLen), as.integer(lnDim), out = double(chnLen*lnDim))$out,
+                 nrow = chnLen, byrow = T)
> chn3 <- matrix(.C("GSLmatLoad",
+         "NSCchains1000/BV_20_1_1000_3.gbin",
+         as.integer(chnLen), as.integer(lnDim), out = double(chnLen*lnDim))$out,
+                 nrow = chnLen, byrow = T)
> chn4 <- matrix(.C("GSLmatLoad",
+         "NSCchains1000/BV_20_1_1000_4.gbin",
+         as.integer(chnLen), as.integer(lnDim), out = double(chnLen*lnDim))$out,
+                 nrow = chnLen, byrow = T)
> chn5 <- matrix(.C("GSLmatLoad",
+         "NSCchains1000/BV_20_1_1000_5.gbin",
+         as.integer(chnLen), as.integer(lnDim), out = double(chnLen*lnDim))$out,
+                 nrow = chnLen, byrow = T)
>
```

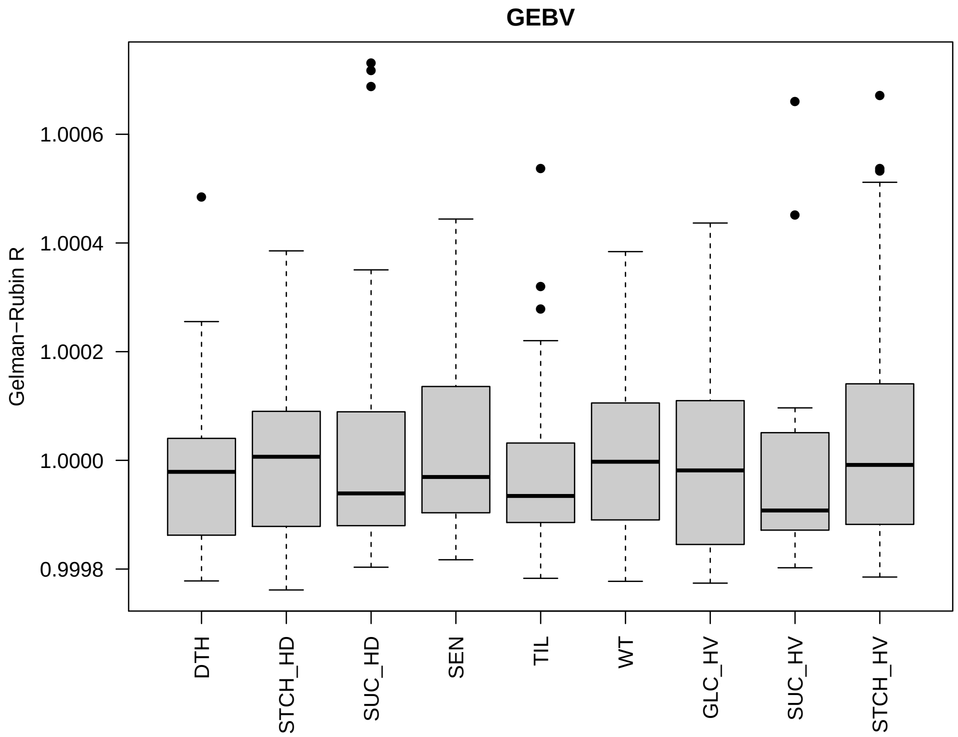

No detectable convergence problems here, either. Make point estimate and error matrices.

```
> tmpMat        <- rbind(chn1, chn2, chn3, chn4, chn5)
> fullBVmatN    <- matrix(apply(tmpMat, 2, pmode), ncol = d, byrow = T)
> fullBVmatNcv <- matrix(apply(tmpMat, 2, sd), ncol = d, byrow = T)/abs(fullBVmatN)
> colnames(fullBVmatN)   <- trt.list
> colnames(fullBVmatNcv) <- trt.list
```

Finally, I look at covariance matrices. We only need the upper triangles.

```
> upInd <- matrix(1:(d^2), ncol = d, byrow = T)
> upInd <- upInd[row(upInd) <= col(upInd)]
> levFac <- rep(c("e","s","a"), each = length(upInd))
> tmpInd  <- matrix(1:(d^2), ncol = d, byrow = T)
> tmpInd  <- tmpInd[row(tmpInd) < col(tmpInd)]
> diagInd <- which(!(upInd %in% tmpInd))
> rm(tmpInd)
> chn1 <- matrix(.C("GSLmatLoad",
+         "NSCchains1000/SigE_20_1_1000_1.gbin",
+         as.integer(chnLen), as.integer(d^2), out = double(chnLen*d^2))$out,
+                 nrow = chnLen, byrow = T)[,upInd]
> chn1 <- cbind(chn1,
+         matrix(.C("GSLmatLoad",
+         "NSCchains1000/SigS_20_1_1000_1.gbin",
```

```
+         as.integer(chnLen), as.integer(d^2), out = double(chnLen*d^2))$out,
+                 nrow = chnLen, byrow = T)[,upInd])
> chn1 <- cbind(chn1,
+         matrix(.C("GSLmatLoad",
+         "NSCchains1000/SigA_20_1_1000_1.gbin",
+         as.integer(chnLen), as.integer(d^2), out = double(chnLen*d^2))$out,
+                 nrow = chnLen, byrow = T)[,upInd])
> chn2 <- matrix(.C("GSLmatLoad",
+         "NSCchains1000/SigE_20_1_1000_2.gbin",
+         as.integer(chnLen), as.integer(d^2), out = double(chnLen*d^2))$out,
+                 nrow = chnLen, byrow = T)[,upInd]
> chn2 <- cbind(chn2,
+         matrix(.C("GSLmatLoad",
+         "NSCchains1000/SigS_20_1_1000_2.gbin",
+         as.integer(chnLen), as.integer(d^2), out = double(chnLen*d^2))$out,
+                 nrow = chnLen, byrow = T)[,upInd])
> chn2 <- cbind(chn2,
+         matrix(.C("GSLmatLoad",
+         "NSCchains1000/SigA_20_1_1000_2.gbin",
+         as.integer(chnLen), as.integer(d^2), out = double(chnLen*d^2))$out,
+                 nrow = chnLen, byrow = T)[,upInd])
> chn3 <- matrix(.C("GSLmatLoad",
+         "NSCchains1000/SigE_20_1_1000_3.gbin",
+         as.integer(chnLen), as.integer(d^2), out = double(chnLen*d^2))$out,
+                 nrow = chnLen, byrow = T)[,upInd]
> chn3 <- cbind(chn3,
+         matrix(.C("GSLmatLoad",
+         "NSCchains1000/SigS_20_1_1000_3.gbin",
+         as.integer(chnLen), as.integer(d^2), out = double(chnLen*d^2))$out,
+                 nrow = chnLen, byrow = T)[,upInd])
> chn3 <- cbind(chn3,
+         matrix(.C("GSLmatLoad",
+         "NSCchains1000/SigA_20_1_1000_3.gbin",
+         as.integer(chnLen), as.integer(d^2), out = double(chnLen*d^2))$out,
+                 nrow = chnLen, byrow = T)[,upInd])
> chn4 <- matrix(.C("GSLmatLoad",
+         "NSCchains1000/SigE_20_1_1000_4.gbin",
+         as.integer(chnLen), as.integer(d^2), out = double(chnLen*d^2))$out,
+                 nrow = chnLen, byrow = T)[,upInd]
> chn4 <- cbind(chn4,
+         matrix(.C("GSLmatLoad",
+         "NSCchains1000/SigS_20_1_1000_4.gbin",
+         as.integer(chnLen), as.integer(d^2), out = double(chnLen*d^2))$out,
+                 nrow = chnLen, byrow = T)[,upInd])
> chn4 <- cbind(chn4,
```

```
+         matrix(.C("GSLmatLoad",
+         "NSCchains1000/SigA_20_1_1000_4.gbin",
+         as.integer(chnLen), as.integer(d^2), out = double(chnLen*d^2))$out,
+                 nrow = chnLen, byrow = T)[,upInd])
> chn5 <- matrix(.C("GSLmatLoad",
+         "NSCchains1000/SigE_20_1_1000_5.gbin",
+         as.integer(chnLen), as.integer(d^2), out = double(chnLen*d^2))$out,
+                 nrow = chnLen, byrow = T)[,upInd]
> chn5 <- cbind(chn5,
+         matrix(.C("GSLmatLoad",
+         "NSCchains1000/SigS_20_1_1000_5.gbin",
+         as.integer(chnLen), as.integer(d^2), out = double(chnLen*d^2))$out,
+                 nrow = chnLen, byrow = T)[,upInd])
> chn5 <- cbind(chn5,
+         matrix(.C("GSLmatLoad",
+         "NSCchains1000/SigA_20_1_1000_5.gbin",
+         as.integer(chnLen), as.integer(d^2), out = double(chnLen*d^2))$out,
+                 nrow = chnLen, byrow = T)[,upInd])
```

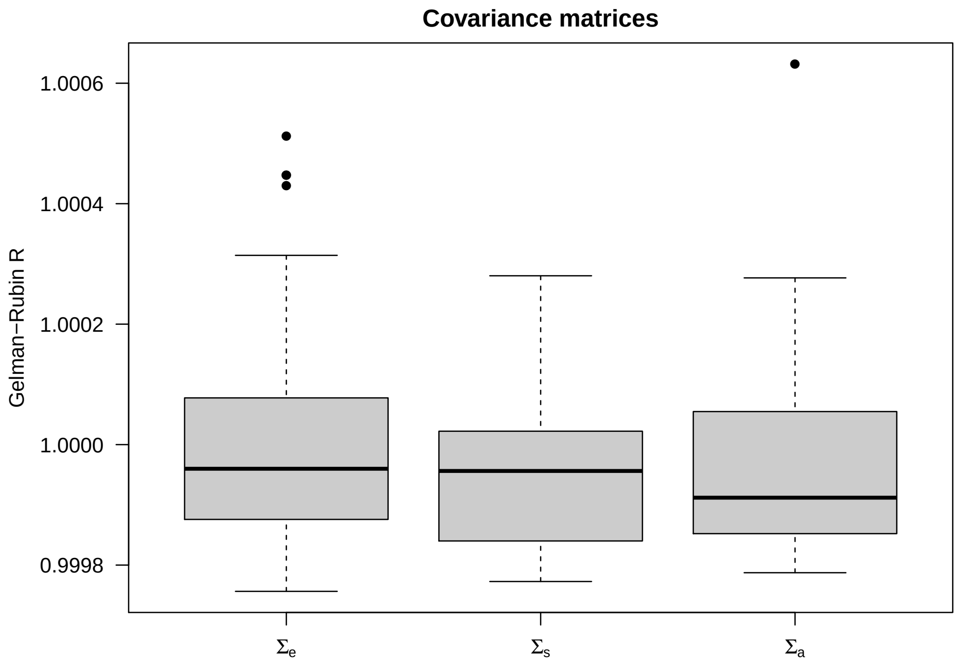

No discernible convergence problems. To make sure, I plot Markov chains for variances, starting from σ_e^2.

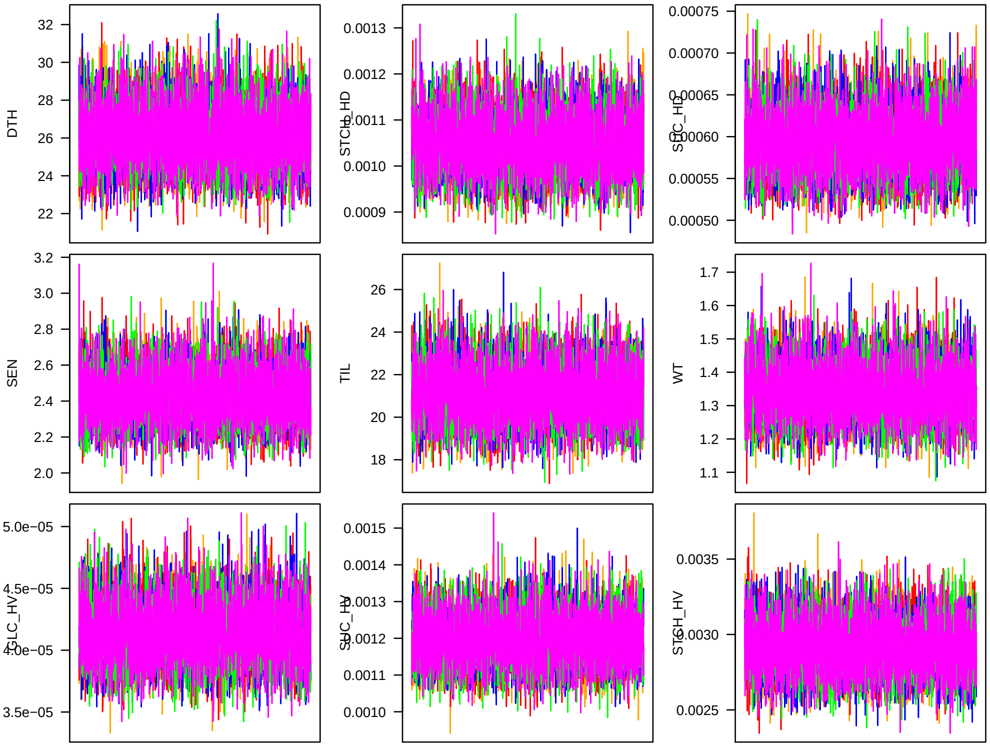

Then the non-additive variances.

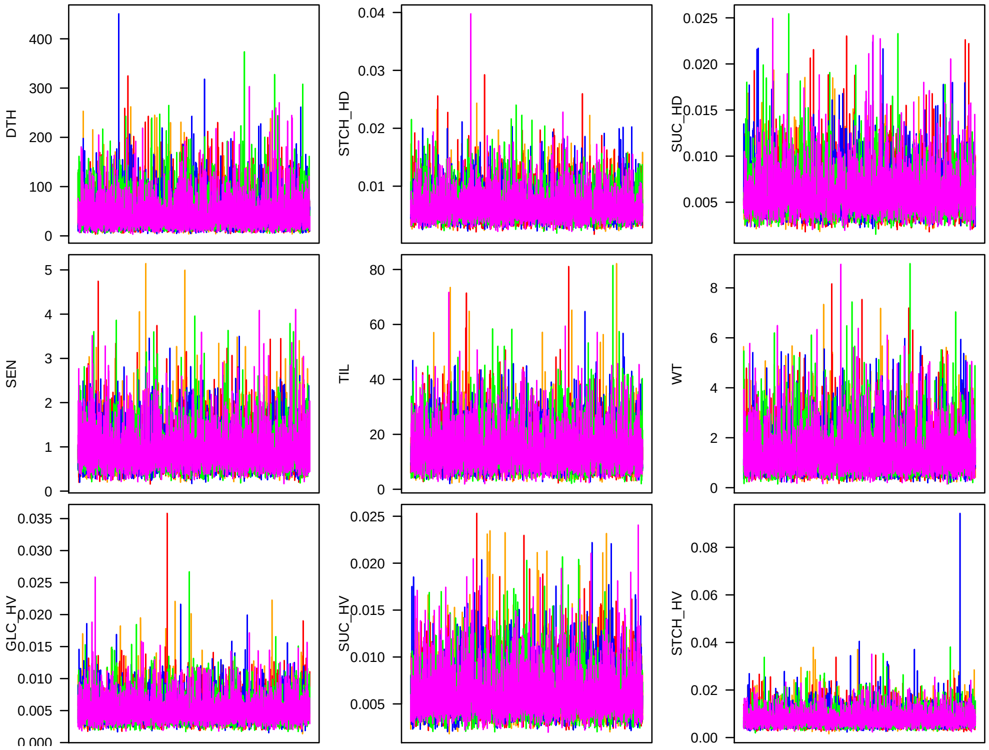

Finally, the additive.

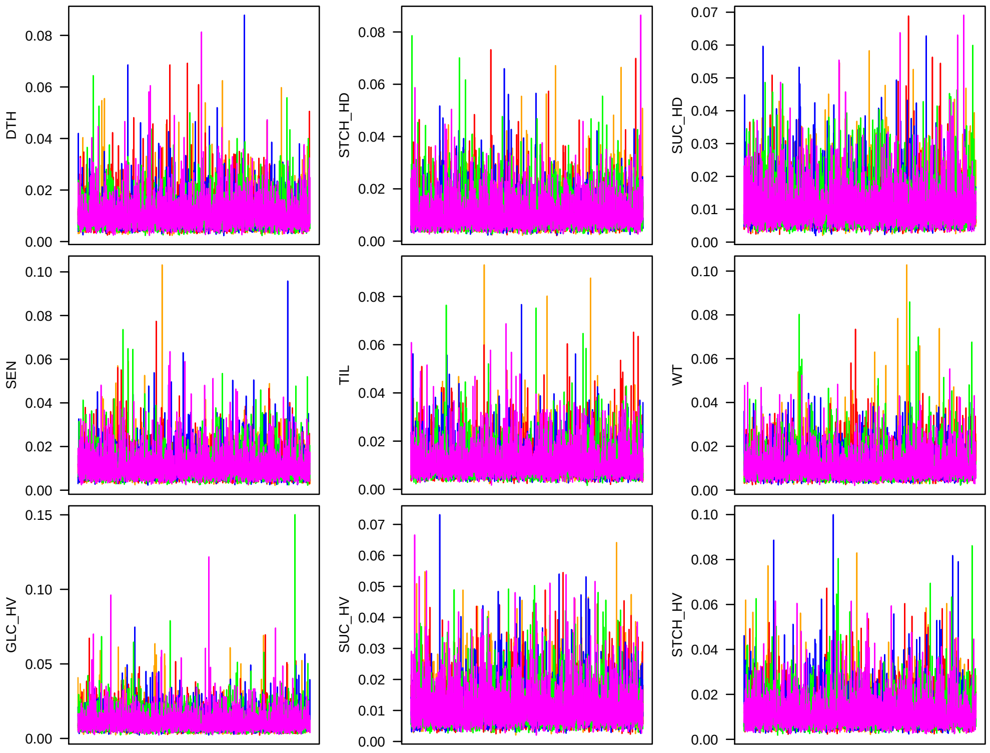

I save genome-estimated narrow-sense heritabilities. For trait k, they are calculated as

$$h_k^2 = \frac{\boldsymbol{\Sigma}^a_{k,k}}{\boldsymbol{\Sigma}^a_{k,k} + \boldsymbol{\Sigma}^s_{k,k} + \boldsymbol{\Sigma}^e_{k,k}}$$

I also save their MCMC errors.

```
> tmpMat <- rbind(chn1, chn2, chn3, chn4, chn5)
> hrMat  <- tmpMat[,levFac == "a"][,diagInd]/(
+          tmpMat[, levFac == "a"][, diagInd] + tmpMat[, levFac == "s"][,diagInd]
+                  + tmpMat[, levFac == "e"][,diagInd]
+          )
> fullHvecN              <- apply(hrMat, 2, pmode)
> names(fullHvecN)       <- trt.list
> fullHvecNci            <- apply(hrMat, 2, quantile, c(0.025, 0.975))
> colnames(fullHvecNci) <- trt.list
> fullHvecNcv            <- apply(hrMat, 2, sd)/abs(fullHvecN)
> names(fullHvecNcv)     <- trt.list
```

Individual variances are:

```
> fullEvecN              <- apply(tmpMat[,levFac == "e"][,diagInd], 2, pmode)
> names(fullEvecN)       <- trt.list
> fullEvecNci            <- apply(tmpMat[,levFac == "e"][,diagInd], 2,
+                                                          quantile, c(0.025, 0.975))
```

```
> colnames(fullEvecNci) <- trt.list
> fullEvecNcv           <- apply(tmpMat[,levFac == "e"][,diagInd], 2, sd)/abs(fullEvecN)
> names(fullEvecNcv)    <- trt.list
> fullSvecN             <- apply(tmpMat[,levFac == "s"][,diagInd], 2, pmode)
> names(fullSvecN)      <- trt.list
> fullSvecNci           <- apply(tmpMat[,levFac == "s"][,diagInd], 2,
+                                                          quantile, c(0.025, 0.975))
> colnames(fullSvecNci) <- trt.list
> fullSvecNcv           <- apply(tmpMat[,levFac == "s"][,diagInd], 2, sd)/abs(fullSvecN)
> names(fullSvecNcv)    <- trt.list
> fullAvecN             <- apply(tmpMat[,levFac == "a"][,diagInd], 2, pmode)
> names(fullAvecN)      <- trt.list
> fullAvecNci           <- apply(tmpMat[,levFac == "a"][,diagInd], 2,
+                                                          quantile, c(0.025, 0.975))
> colnames(fullAvecNci) <- trt.list
> fullAvecNcv           <- apply(tmpMat[,levFac == "a"][,diagInd], 2, sd)/abs(fullAvecN)
> names(fullAvecNcv)    <- trt.list
> round(fullHvecN,4)
```

```
    DTH STCH_HD  SUC_HD     SEN     TIL      WT  GLC_HV  SUC_HV STCH_HV
 0.0001  0.5615  0.6080  0.0022  0.0002  0.0027  0.6910  0.5838  0.4873
```

```
> round(fullHvecNci,4)
```

```
        DTH STCH_HD SUC_HD    SEN   TIL     WT GLC_HV SUC_HV STCH_HV
2.5%  0e+00  0.2969 0.3310 0.0011 1e-04 0.0012 0.3757 0.3158  0.2327
97.5% 5e-04  0.8111 0.8392 0.0084 9e-04 0.0119 0.8726 0.8186  0.7677
```

```
> round(fullEvecN,4)
```

```
    DTH STCH_HD  SUC_HD     SEN     TIL      WT  GLC_HV  SUC_HV STCH_HV
25.9460  0.0010  0.0006  2.4166 20.9881  1.3361  0.0000  0.0012  0.0029
```

```
> round(fullEvecNci,4)
```

```
          DTH STCH_HD SUC_HD    SEN     TIL     WT GLC_HV SUC_HV STCH_HV
2.5%  23.1739  0.0009  5e-04 2.1650 18.7130 1.1911      0 0.0011  0.0026
97.5% 29.4563  0.0012  7e-04 2.7395 23.7298 1.5094      0 0.0013  0.0033
```

```
> round(fullSvecN,4)
```

```
    DTH STCH_HD  SUC_HD     SEN     TIL      WT  GLC_HV  SUC_HV STCH_HV
24.3776  0.0054  0.0048  0.7063 10.2538  0.8726  0.0040  0.0050  0.0063
```

```
> round(fullSvecNci,4)

           DTH STCH_HD SUC_HD    SEN     TIL     WT GLC_HV SUC_HV STCH_HV
2.5%    9.4746  0.0031 0.0028 0.3425  4.5860 0.3822 0.0024 0.0029  0.0036
97.5% 137.3751  0.0134 0.0122 2.1230 33.4675 3.7098 0.0107 0.0124  0.0171


> round(fullAvecN,4)

    DTH STCH_HD  SUC_HD     SEN     TIL      WT  GLC_HV  SUC_HV STCH_HV
 0.0072  0.0074  0.0073  0.0074  0.0074  0.0073  0.0075  0.0074  0.0078


> round(fullAvecNci,4)

         DTH STCH_HD SUC_HD    SEN    TIL     WT GLC_HV SUC_HV STCH_HV
2.5%  0.0039  0.0038 0.0039 0.0039 0.0039 0.0039 0.0039  0.004  0.0039
97.5% 0.0258  0.0274 0.0276 0.0278 0.0282 0.0283 0.0280  0.028  0.0306
```

Now I save the values and their credible intervals to a file. These will be used for display rather than further analyses, so I am rounding them to three significant digits.

```
> cat("trait_name\th^2\tsigma^2_e\tsigma^2_s\tsigma^2_e",
+         apply(
+                 cbind(trt.list, rep("\t", d),
+                         signif(fullHvecN,3),
+                         rep(" (", d), signif(fullHvecNci[1,],3),
+                         rep(", ", d), signif(fullHvecNci[2,],3), rep(")\t", d),
+                         signif(fullEvecN,3),
+                         rep(" (", d), signif(fullEvecNci[1,],3),
+                         rep(", ", d), signif(fullEvecNci[2,],3), rep(")\t", d),
+                         signif(fullSvecN,3),
+                         rep(" (", d), signif(fullSvecNci[1,],3),
+                         rep(", ", d), signif(fullSvecNci[2,],3), rep(")\t", d),
+                         signif(fullAvecN,3),
+                         rep(" (", d), signif(fullAvecNci[1,],3),
+                         rep(", ", d), signif(fullAvecNci[2,],3), rep(")\t", d)
+                         ),
+                 1, paste, collapse=""),
+                 file = "variancesGaussian.tsv", sep = "\n")
>
```

I now look at genetic correlations from the $\mathbf{\Sigma}_e$ and $\mathbf{\Sigma}_a$ matrices. Since I only read in upper triangles of the covariance matrices, I have to write a function that that transforms covariances into correlations, using variances that are on the diagonals. The function works on each MCMC sample.

```
> cov2corTri <- cmpfun(function(vec){
+         tmp <- matrix(1, d, d)
+         tmp[upper.tri(tmp)] <- vec[-diagInd]
+
+         isdMat <- sapply(1/sqrt(vec[diagInd]), rep, d)
+         tmp    <- tmp*isdMat*t(isdMat)
+         return(tmp[upper.tri(tmp)])
+ })
```

I run this function on the environmental and additive genetic portions of the covariance MCMC matrix (`tmpMat`), keeping in mind that the `apply()` function returns each result in a column, thus the MCMC chains are the rows of resulting matrices (as opposed to clumns in `tmpMat`).

```
> eCorMCMC <- apply(tmpMat[,levFac == "e"], 1, cov2corTri)
> aCorMCMC <- apply(tmpMat[,levFac == "a"], 1, cov2corTri)
```

I calculate point estimates and 95% credible intervals for each correlation value.

```
> eCorMnN   <- apply(eCorMCMC, 1, pmode)
> eCorMnNci <- apply(eCorMCMC, 1, quantile, c(0.025, 0.975))
> aCorMnN   <- apply(aCorMCMC, 1, pmode)
> aCorMnNci <- apply(aCorMCMC, 1, quantile, c(0.025, 0.975))
```

I construct a $d \times d$ matrix that has 1.0 on the diagonal, additive correlations in the upper and environmental correlations in the lower triangle. I repeat that for lower and upper bounds of the credible intervals.

```
> corMatN <- matrix(1, d, d)
> colnames(corMatN) <- trt.list
> rownames(corMatN) <- trt.list
> corMatN[upper.tri(corMatN)] <- aCorMnN
> corMatNl <- matrix(1, d, d)
> corMatNl[upper.tri(corMatNl)] <- aCorMnNci[1,]
> corMatNu <- matrix(1, d, d)
> corMatNu[upper.tri(corMatNu)] <- aCorMnNci[2,]
```

Putting the environmental correlations in the lower triangle is not as easy, since they come from the upper triangle of the covariance matrix. It requires the use of a temporary matrix.

```
> tmpCmat <- matrix(1, d, d)
> tmpCmat[upper.tri(tmpCmat)]   <- eCorMnN
> corMatN[lower.tri(corMatN)]   <- t(tmpCmat)[lower.tri(tmpCmat)]
> tmpCmat[upper.tri(tmpCmat)]   <- eCorMnNci[1,]
> corMatNl[lower.tri(corMatNl)] <- t(tmpCmat)[lower.tri(tmpCmat)]
> tmpCmat[upper.tri(tmpCmat)]   <- eCorMnNci[2,]
> corMatNu[lower.tri(corMatNu)] <- t(tmpCmat)[lower.tri(tmpCmat)]
> rm(tmpCmat)
> signif(corMatN, 3)
```

```
             DTH  STCH_HD   SUC_HD       SEN       TIL        WT    GLC_HV   SUC_HV  STCH_HV
DTH       1.0000 -0.00958  0.01720 -0.00202  0.03110  0.00358  0.007860 -0.00322  0.01190
STCH_HD   0.0878  1.00000 -0.00385  0.00620 -0.00796  0.00473  0.000815  0.01180 -0.00297
SUC_HD    0.0604  0.19900  1.00000  0.00524 -0.00683  0.00343 -0.026100  0.03500  0.02670
SEN       0.1030  0.03610  0.01170  1.00000  0.00330 -0.00573 -0.005980  0.03050  0.00815
TIL       0.0541  0.10500  0.04460  0.09620  1.00000 -0.01570 -0.026800 -0.00388  0.00952
WT        0.0282  0.04660  0.10800 -0.11700 -0.11100  1.00000 -0.007090  0.00968 -0.02800
GLC_HV    0.0800  0.07510  0.13400  0.12300  0.08380  0.03940  1.000000 -0.00409  0.02300
SUC_HV   -0.0348 -0.01310  0.11500 -0.25500  0.01670  0.16800  0.212000  1.00000  0.01610
STCH_HV   0.1190  0.03170  0.13600 -0.11400 -0.07740  0.56200  0.061000  0.27800  1.00000
```

```
> signif(corMatNu, 3)

        [,1]  [,2]   [,3]    [,4]     [,5]   [,6]  [,7]  [,8]  [,9]
 [1,] 1.0000 0.465 0.5340  0.5370  0.53000 0.5340 0.530 0.529 0.537
 [2,] 0.1340 1.000 0.4660  0.5290  0.53500 0.5330 0.535 0.524 0.552
 [3,] 0.1500 0.234 1.0000  0.4510  0.53400 0.5290 0.534 0.537 0.551
 [4,] 0.1870 0.119 0.0555  1.0000  0.45100 0.5320 0.530 0.525 0.536
 [5,] 0.1410 0.186 0.1300  0.1340  1.00000 0.4350 0.533 0.528 0.539
 [6,] 0.1120 0.131 0.1920 -0.0346 -0.05690 1.0000 0.426 0.529 0.544
 [7,] 0.1620 0.156 0.2130  0.2030  0.16600 0.0909 1.000 0.404 0.549
 [8,] 0.0476 0.072 0.1930 -0.1750  0.10400 0.2390 0.246 1.000 0.411
 [9,] 0.2030 0.116 0.2170 -0.0311  0.00827 0.6170 0.143 0.310 1.000
```

```
> signif(corMatNl, 3)

         [,1]    [,2]    [,3]    [,4]      [,5]    [,6]    [,7]   [,8]   [,9]
 [1,]  1.0000 -0.4650 -0.5300 -0.5400 -0.531000 -0.5320 -0.5330 -0.526 -0.549
 [2,]  0.0418  1.0000 -0.4710 -0.5340 -0.526000 -0.5330 -0.5330 -0.531 -0.547
 [3,] -0.0262  0.1680  1.0000 -0.4580 -0.538000 -0.5330 -0.5330 -0.526 -0.540
 [4,]  0.0186 -0.0490 -0.0296  1.0000 -0.437000 -0.5290 -0.5300 -0.536 -0.538
 [5,] -0.0330  0.0173 -0.0406  0.0585  1.000000 -0.4420 -0.5330 -0.530 -0.545
 [6,] -0.0567 -0.0390  0.0232 -0.1980 -0.162000  1.0000 -0.4160 -0.533 -0.542
 [7,] -0.0037 -0.0106  0.0480  0.0380  0.000444 -0.0131  1.0000 -0.409 -0.551
 [8,] -0.1240 -0.0978  0.0246 -0.3320 -0.064600  0.0969  0.1790  1.000 -0.408
 [9,]  0.0328 -0.0544  0.0527 -0.1940 -0.161000  0.5030 -0.0192  0.251  1.000
```

Credible intervals for additive genetic correlations are very broad, reflecting the low sample size relative to the number of traits. I therefore switch to estimating these correlations by calculating their values directly from MCMC samples of GEBVs.

```
> chn1 <- matrix(.C("GSLmatLoad",
+          "NSCchains1000/BV_20_1_1000_1.gbin",
+          as.integer(chnLen), as.integer(lnDim), out = double(chnLen*lnDim))$out,
+                  nrow = chnLen, byrow = T)
> chn2 <- matrix(.C("GSLmatLoad",
+          "NSCchains1000/BV_20_1_1000_2.gbin",
+          as.integer(chnLen), as.integer(lnDim), out = double(chnLen*lnDim))$out,
```

```
+                 nrow = chnLen, byrow = T)
> chn3 <- matrix(.C("GSLmatLoad",
+         "NSCchains1000/BV_20_1_1000_3.gbin",
+         as.integer(chnLen), as.integer(lnDim), out = double(chnLen*lnDim))$out,
+                 nrow = chnLen, byrow = T)
> chn4 <- matrix(.C("GSLmatLoad",
+         "NSCchains1000/BV_20_1_1000_4.gbin",
+         as.integer(chnLen), as.integer(lnDim), out = double(chnLen*lnDim))$out,
+                 nrow = chnLen, byrow = T)
> chn5 <- matrix(.C("GSLmatLoad",
+         "NSCchains1000/BV_20_1_1000_5.gbin",
+         as.integer(chnLen), as.integer(lnDim), out = double(chnLen*lnDim))$out,
+                 nrow = chnLen, byrow = T)
> tmpMat       <- rbind(chn1, chn2, chn3, chn4, chn5)
> sampCor <- cmpfun(function(vec){
+         res <- cor(matrix(vec, ncol = d, byrow = T))
+         return(res[upper.tri(res)])
+ })
> GCorNmat    <- apply(tmpMat, 1, sampCor)
> fullGCorN   <- apply(GCorNmat, 1, pmode)
> fullGCorNci <- apply(GCorNmat, 1, quantile, c(0.025, 0.975))
> corMatN[upper.tri(corMatN)]   <- fullGCorN
> corMatNl[upper.tri(corMatNl)] <- fullGCorNci[1,]
> corMatNu[upper.tri(corMatNu)] <- fullGCorNci[2,]
> signif(corMatN, 3)
```

```
            DTH STCH_HD SUC_HD     SEN     TIL      WT  GLC_HV  SUC_HV STCH_HV
DTH      1.0000  0.4680 0.3330  0.0484  0.4150  0.3370  0.1150  0.2910  0.6170
STCH_HD  0.0878  1.0000 0.1910 -0.0840  0.2220  0.4690  0.0599  0.0777  0.4640
SUC_HD   0.0604  0.1990 1.0000 -0.0188  0.3410  0.2180  0.0883  0.2470  0.1230
SEN      0.1030  0.0361 0.0117  1.0000  0.1890 -0.3630 -0.0181 -0.1240 -0.0905
TIL      0.0541  0.1050 0.0446  0.0962  1.0000  0.0606  0.1220  0.1790  0.0417
WT       0.0282  0.0466 0.1080 -0.1170 -0.1110  1.0000  0.0548  0.3260  0.5090
GLC_HV   0.0800  0.0751 0.1340  0.1230  0.0838  0.0394  1.0000  0.0381  0.0287
SUC_HV  -0.0348 -0.0131 0.1150 -0.2550  0.0167  0.1680  0.2120  1.0000  0.1110
STCH_HV  0.1190  0.0317 0.1360 -0.1140 -0.0774  0.5620  0.0610  0.2780  1.0000
```

Now save the correlation matrix to a file. Each cell in the table has the upper-bound, point estimate and lower-bound value in a column.

```
> cat(paste(c("trait_names", trt.list), collapse = "\t"),
+         file = "correlationMatrixN.tsv", sep = "\n")
> for (trt in 1:d){
+         cat(paste(c("", signif(corMatNu[trt,], 3)), collapse = "\t"),
+                 file = "correlationMatrixN.tsv", sep = "\n", append = T)
+         cat(paste(c(trt.list[trt], signif(corMatN[trt,], 3)), collapse = "\t"),
+                 file = "correlationMatrixN.tsv", sep = "\n", append = T)
```

```
+         cat(paste(c("", signif(corMatNl[trt,], 3)), collapse = "\t"),
+                 file = "correlationMatrixN.tsv", sep = "\n", append = T)
+ }
```

Now I check convergence for the sub-sampled data, for each number of sub-replicates separately, but concatenating values for all 20 samples.

I encapsulate the basic operation in a function, `subGR()`, that takes three parameters: `iSam` – sampled data ID, `rNum` – number of replicates, `varNam` – name of the variable (`LN` for line means and `BV` for GEBVs), and `mdl` – Gaussian or Student-t model for errors. I calls the function `readChain()` that reads individual chains with the same parameters.

```
> readChain <- cmpfun(function(iChn, iSam, rNum, varNam, mdl){
+          matrix(.C("GSLmatLoad",
+                 paste("NSCchains", mdl, "/", varNam, "_",
+                         rNum, "_", iSam, "_", mdl, "_", iChn, ".gbin", sep = ""),
+                 as.integer(chnLen), as.integer(lnDim), out = double(chnLen*lnDim))$out,
+                         nrow = chnLen, byrow = T)
+ })
> subGR <- cmpfun(function(iSam, rNum, varNam, mdl){
+         chn <- lapply(1:5, readChain, iSam, rNum, varNam, mdl)
+
+         # rowMeans because the result of sapply() will have 5 chains in columns
+         W  <- rowMeans(sapply(chn, function(mat){apply(mat, 2, var)}))
+         B  <- apply(sapply(chn, colMeans), 1, var)
+         GR <- sqrt((((chnLen - 1)/chnLen)*W + B)/W)
+         return(GR)
+ })
> allGR <- cmpfun(function(rNum, varNam, mdl){
+         matrix( array(sapply(1:20, subGR, rNum, varNam, mdl)), ncol = d, byrow = T )
+ })
> LNgr <- lapply(c(2, 5, 10, 15), allGR, "LN", 1000)
> BVgr <- lapply(c(2, 5, 10, 15), allGR, "BV", 1000)
```

Now plot the results for line means:

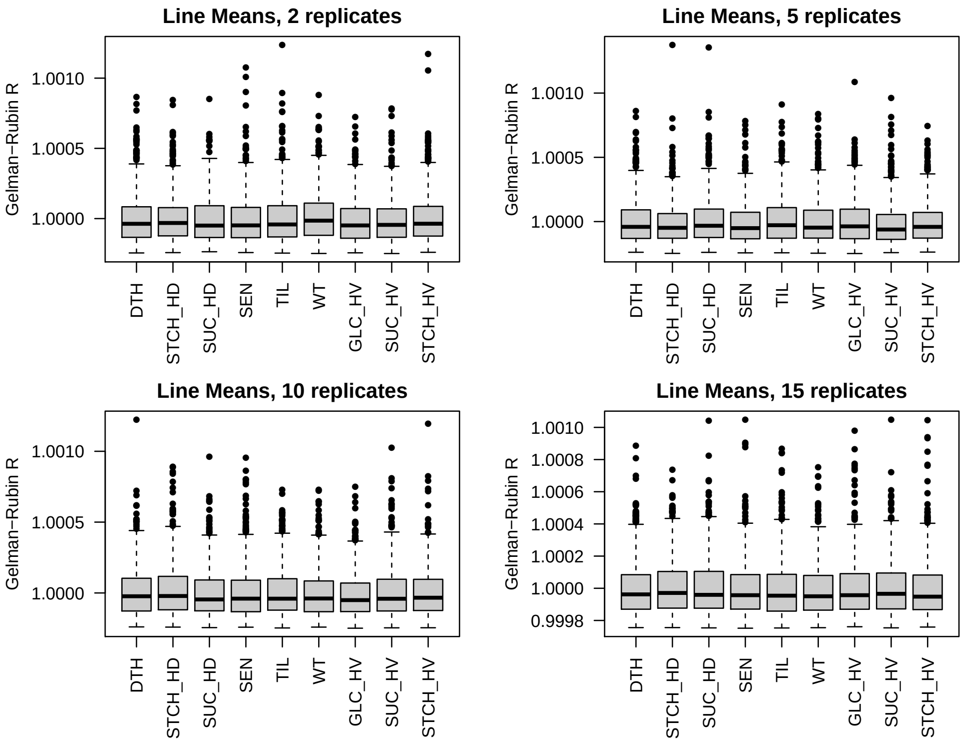

And GEBVs:

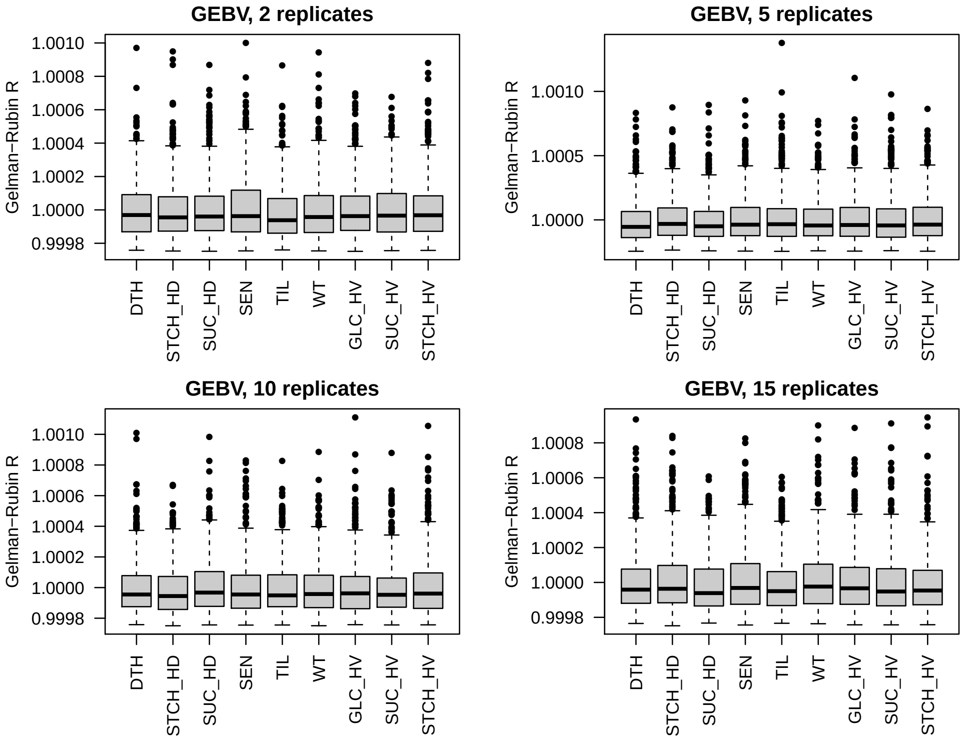

Now I do the same for covariance matrices.

```
> readChainCV <- cmpfun(function(iChn, iSam, rNum, mdl){
+          cbind(
+                  matrix(.C("GSLmatLoad",
+                         paste("NSCchains", mdl, "/SigE_", rNum, "_",
+                                 iSam, "_", mdl, "_", iChn, ".gbin", sep = ""),
+                         as.integer(chnLen), as.integer(d^2),
+                                         out = double(chnLen*d^2))$out,
+                                 nrow = chnLen, byrow = T)[,upInd],
+                  matrix(.C("GSLmatLoad",
+                         paste("NSCchains", mdl, "/SigS_", rNum, "_",
+                                 iSam, "_", mdl, "_", iChn, ".gbin", sep = ""),
+                         as.integer(chnLen), as.integer(d^2),
+                                         out = double(chnLen*d^2))$out,
+                                 nrow = chnLen, byrow = T)[,upInd],
+                  matrix(.C("GSLmatLoad",
+                         paste("NSCchains", mdl, "/SigA_", rNum, "_",
+                                 iSam, "_", mdl, "_", iChn, ".gbin", sep = ""),
+                         as.integer(chnLen), as.integer(d^2),
+                                         out = double(chnLen*d^2))$out,
+                                 nrow = chnLen, byrow = T)[,upInd]
+         )
+ })
> subGRvr <- cmpfun(function(iSam, rNum, mdl){
+         chn <- lapply(1:5, readChainCV, iSam, rNum, mdl)
+
+         # rowMeans because the result of sapply() will have 5 chains in columns
+         W  <- rowMeans(sapply(chn, function(mat){apply(mat, 2, var)}))
+         B  <- apply(sapply(chn, colMeans), 1, var)
+         GR <- sqrt((((chnLen - 1)/chnLen)*W + B)/W)
+         return(GR)
+ })
> allGRvr <- cmpfun(function(rNum, mdl){
+         matrix( array(sapply(1:20, subGRvr, rNum, mdl)), ncol = 3, byrow = T )
+ })
> SigGR <- lapply(c(2, 5, 10, 15), allGRvr, 1000)
```

Now plot the Gelman-Rubin estimates for covariance matrices:

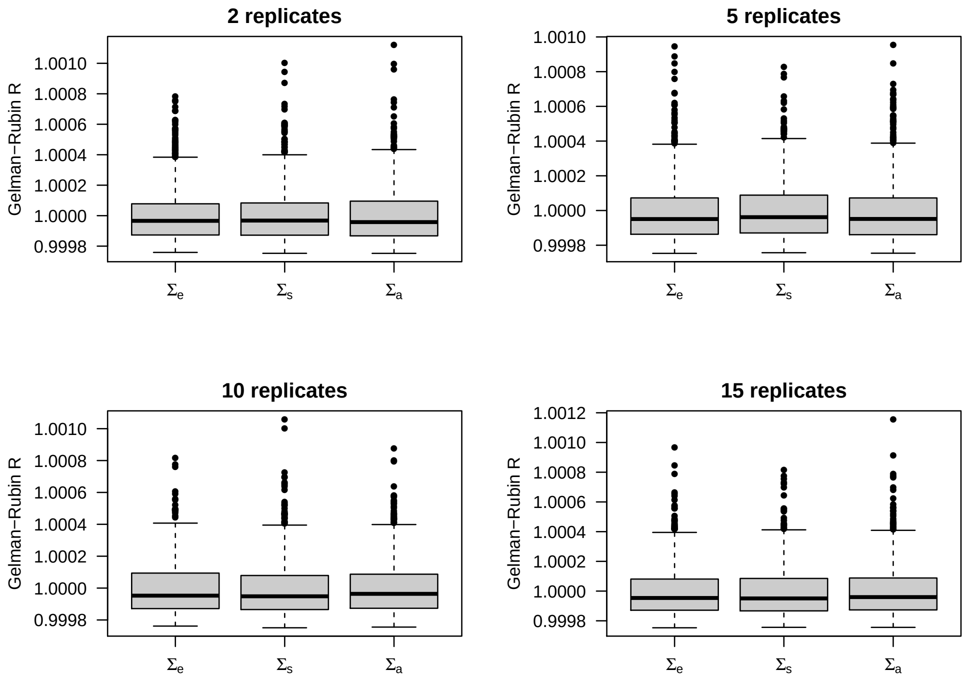

All parameters appear to have converged completely. I can proceed to analyze the results.

First I look at the accuracy of estimates based on sub-sampled data, compared to the full data set, assuming that the most accurate estimates are based on the full data.

I define a function that reads the chains from a given simulation and sub-replicate number and calculates point estimates of specified parameters. The `subMn()` function parameters are as for the `subGR()` function above.

```
> subMn <- cmpfun(function(iSam, rNum, varNam, mdl){
+         chn <- lapply(1:5, readChain, iSam, rNum, varNam, mdl)
+
+         # converting to a matrix with chains as columns
+         chn <- matrix(array(sapply(chn, t)), nrow = chnLen*nChn, byrow = T)
+
+         return(apply(chn, 2, pmode))
+ })
> allMn <- cmpfun(function(rNum, varNam, mdl){
+         matrix( array(sapply(1:20, subMn, rNum, varNam, mdl)), ncol = d, byrow = T )
+ })
> LNmn <- lapply(c(2, 5, 10, 15), allMn, "LN", 1000)
> BVmn <- lapply(c(2, 5, 10, 15), allMn, "BV", 1000)
>
```

Plot accuracies (reflected by correlations of point estimates from sub-sampled data with estimates from the whole data set) by trait, comparing replicate sizes directly on the same plot. First, line means.

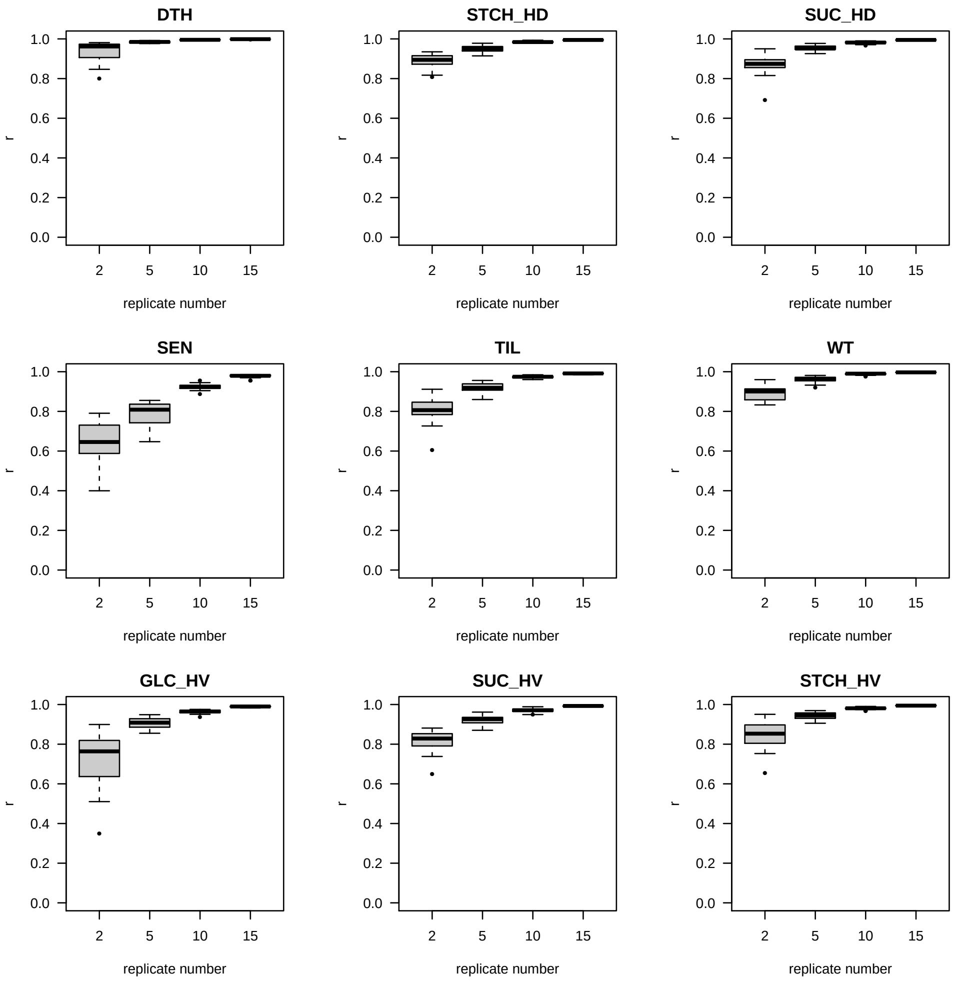
DTH
STCH_HD
SUC_HD
SEN
TIL
WT
GLC_HV
SUC_HV
STCH_HV
r
1.0
0.8
0.6
0.4
0.2
0.0
2
5
10
15
replicate number

Then GEBVs.

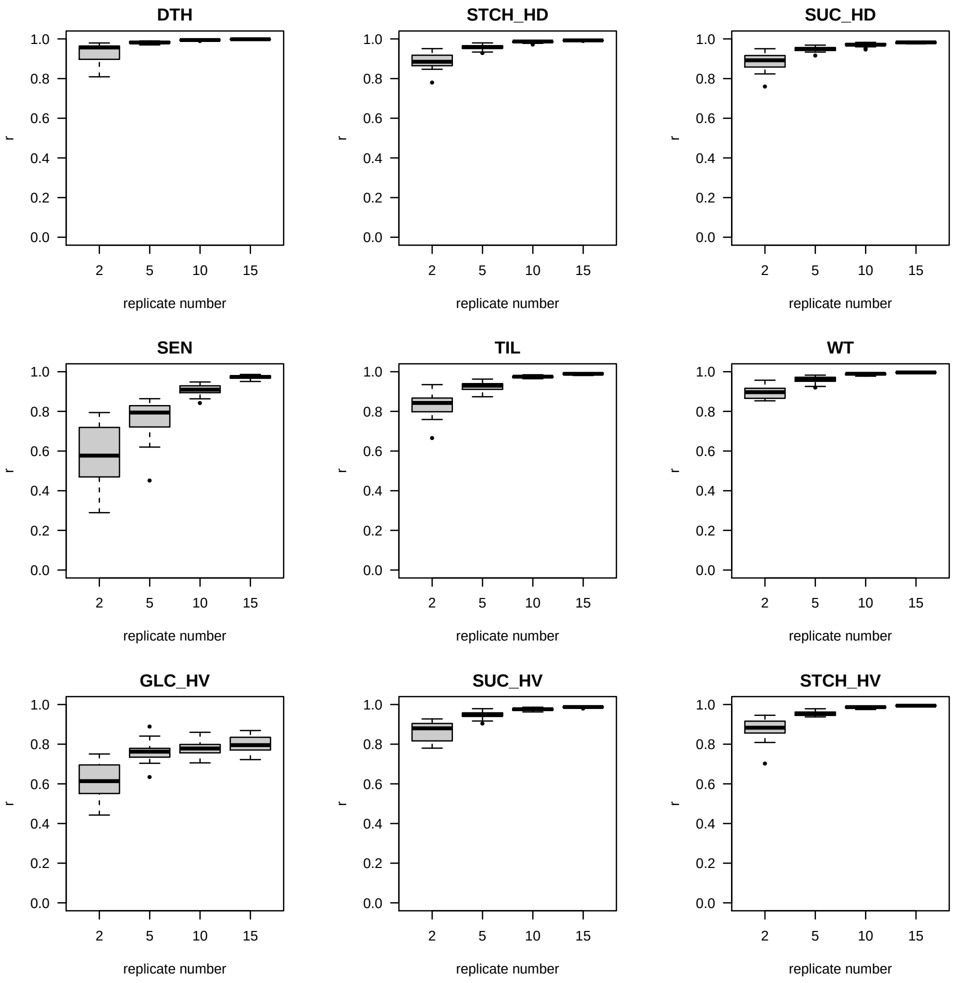

I am also interested in a measure of uncertainty of estimates. I use the coefficient of variation (CV, equal to standard deviation divided by the mean) of the Markov chain as such a measure. First, I calculate this statistic.

```
> subCV <- cmpfun(function(iSam, rNum, varNam, mdl){
+         chn <- lapply(1:5, readChain, iSam, rNum, varNam, mdl)
+
+         # converting to a matrix with chains as columns
+         chn <- matrix(array(sapply(chn, t)), nrow = chnLen*nChn, byrow = T)
+
+         return(apply(chn, 2, sd)/abs(apply(chn, 2, pmode)))
+ })
> allCV <- cmpfun(function(rNum, varNam, mdl){
+         matrix( array(sapply(1:20, subCV, rNum, varNam, mdl)), ncol = d, byrow = T )
+ })
> LNcv <- lapply(c(2, 5, 10, 15), allCV, "LN", 1000)
> BVcv <- lapply(c(2, 5, 10, 15), allCV, "BV", 1000)
```

>

Plot the values for each replicate number, divided by the CVs for the whole data set. Start with line means.

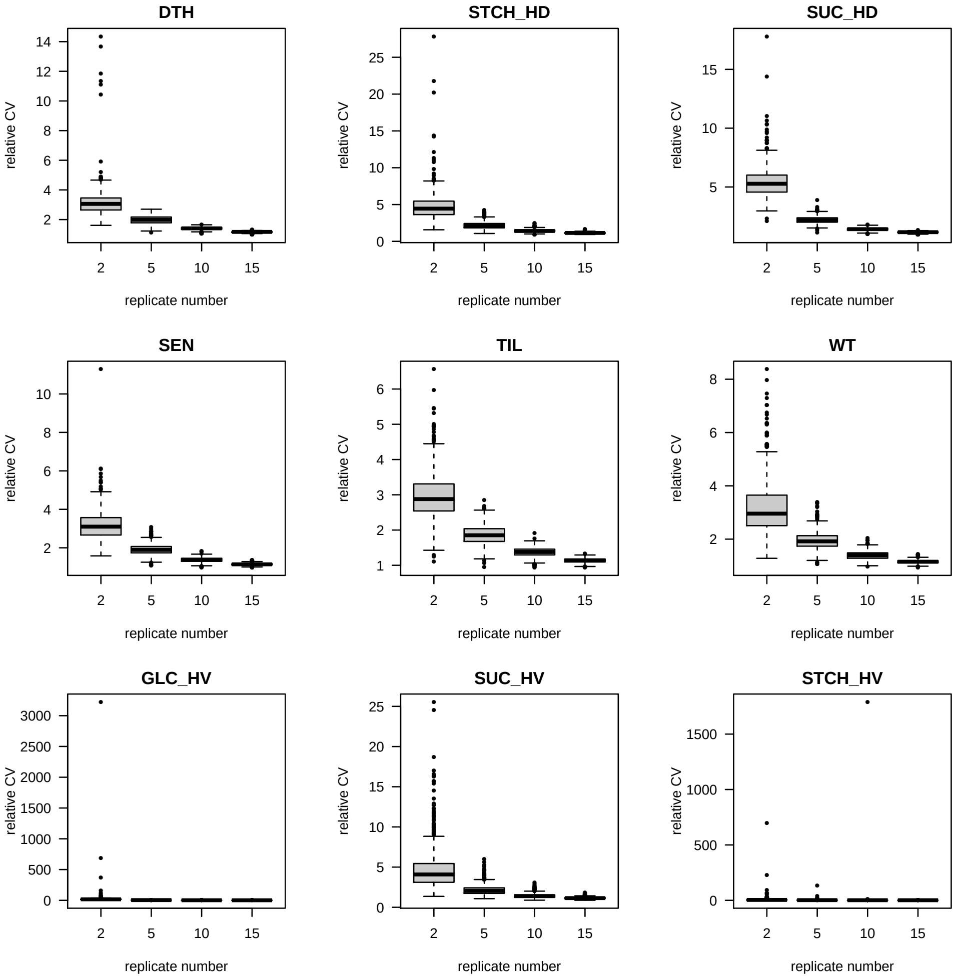

And GEBVs:

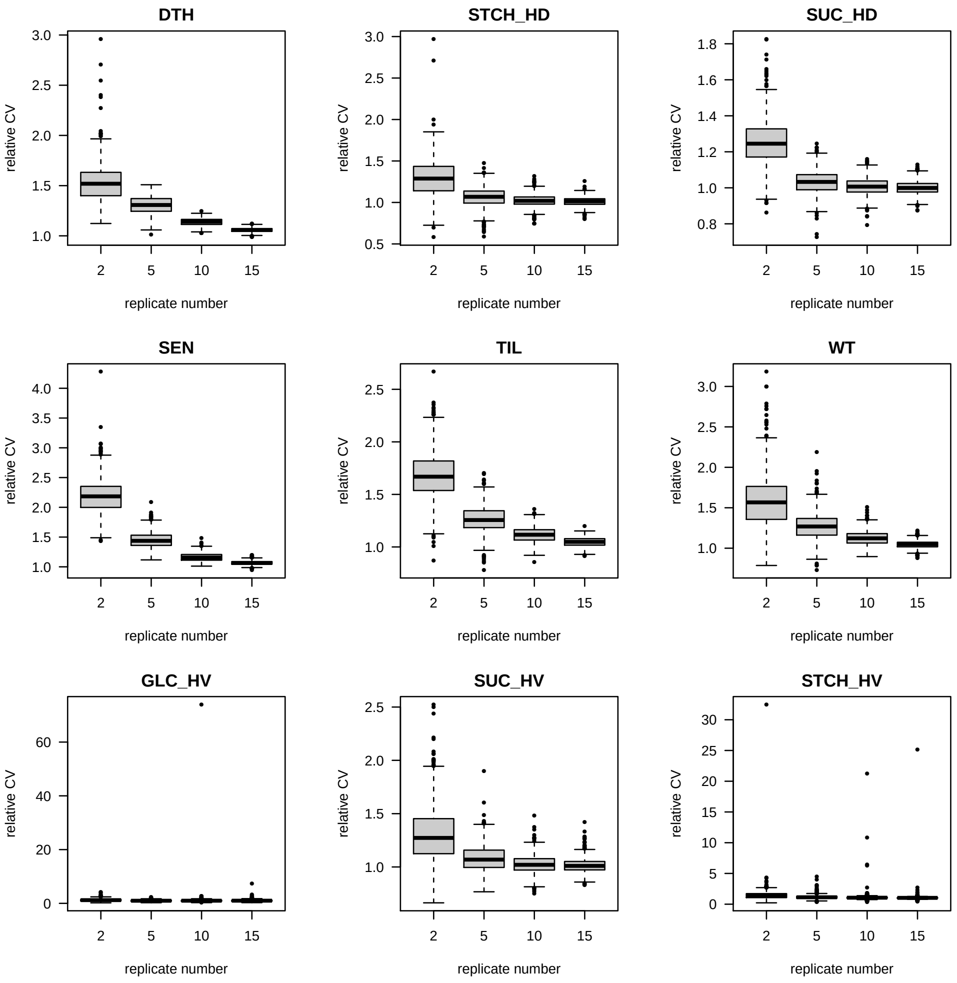

Outliers on the plots obscure the main differnces, so I re-plot without outliers. Line means:

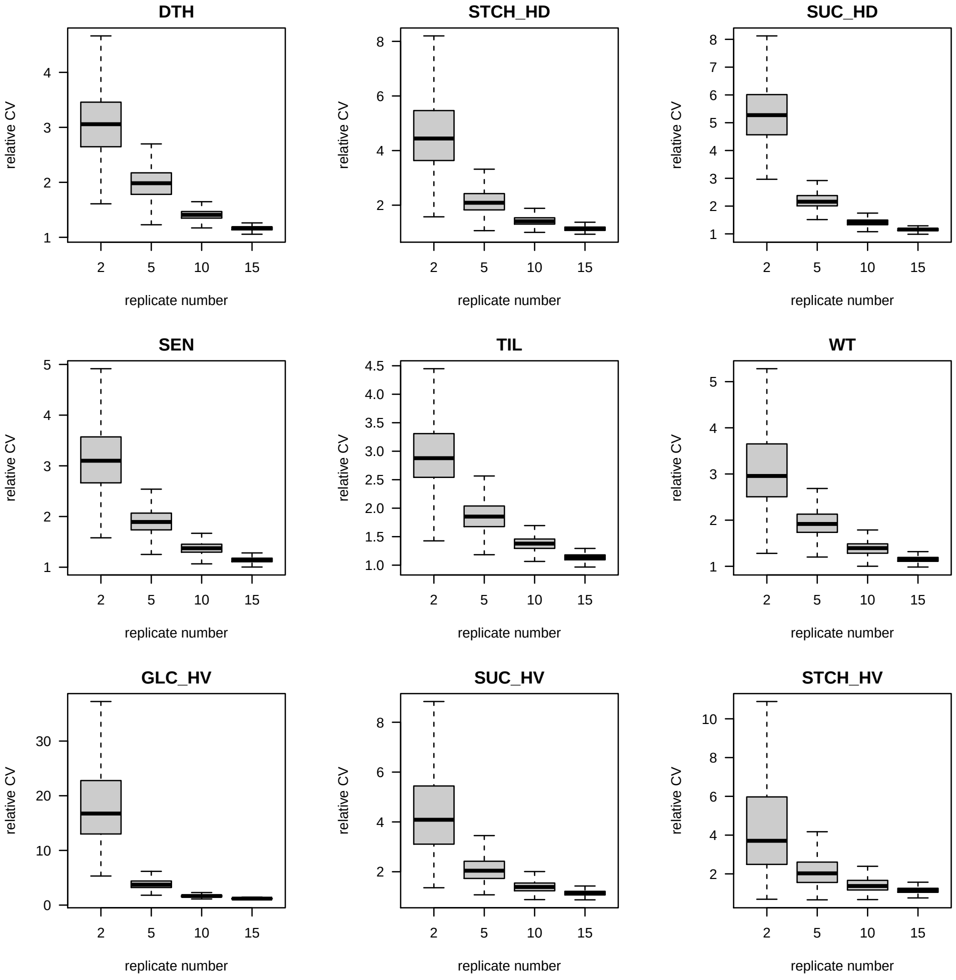
DTH
STCH_HD
SUC_HD
SEN
TIL
WT
GLC_HV
SUC_HV
STCH_HV
relative CV
replicate number
2
5
10
15

And GEBVs:

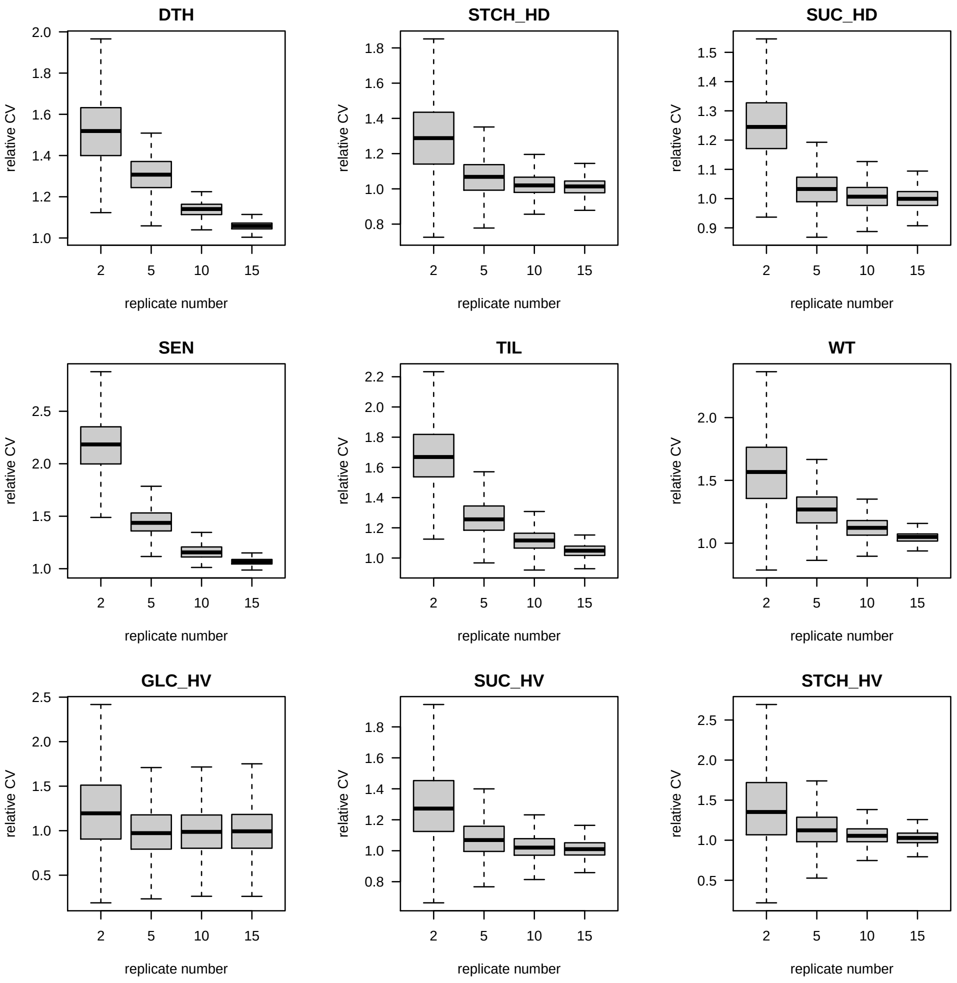

With variances and heritabilities correlation is not a viable metric of accuracy because there is only one value per data set. I therefore use the relative deviation of a sub-sampled `versus` full-data value.

$$RD = \frac{\sigma_s^2 - \sigma_f^2}{\sigma_f^2}$$

I am not taking a norm because I want to see if there is any bias in the estimates.

```
> readChainVR <- cmpfun(function(iChn, iSam, rNum, mdl){
+         cbind(
+                 matrix(.C("GSLmatLoad",
+                         paste("NSCchains", mdl, "/SigE_", rNum, "_",
+                                 iSam, "_", mdl, "_", iChn, ".gbin", sep = ""),
+                         as.integer(chnLen), as.integer(d^2),
+                                         out = double(chnLen*d^2))$out,
+                                 nrow = chnLen, byrow = T)[,upInd][,diagInd],
+                 matrix(.C("GSLmatLoad",
```

```
+                          paste("NSCchains", mdl, "/SigS_", rNum, "_",
+                                  iSam, "_", mdl, "_", iChn, ".gbin", sep = ""),
+                          as.integer(chnLen), as.integer(d^2),
+                                          out = double(chnLen*d^2))$out,
+                                  nrow = chnLen, byrow = T)[,upInd][,diagInd],
+                   matrix(.C("GSLmatLoad",
+                          paste("NSCchains", mdl, "/SigA_", rNum, "_",
+                                  iSam, "_", mdl, "_", iChn, ".gbin", sep = ""),
+                          as.integer(chnLen), as.integer(d^2),
+                                          out = double(chnLen*d^2))$out,
+                                  nrow = chnLen, byrow = T)[,upInd][,diagInd]
+         )
+ })
> subVR <- cmpfun(function(iSam, rNum, mdl){
+         chn <- lapply(1:5, readChainVR, iSam, rNum, mdl)
+
+         # converting to a matrix with chains as columns
+         chn <- matrix(array(sapply(chn, t)), nrow = chnLen*nChn, byrow = T)
+         eachMn <- apply(chn, 2, pmode)
+         mnMat  <- matrix(eachMn, ncol = 3)
+         hr     <- mnMat[,3]/rowSums(mnMat)
+         return(c(eachMn, hr))
+ })
> allVR <- cmpfun(function(rNum, mdl){
+         matrix( array(sapply(1:20, subVR, rNum, mdl)),  ncol = d, byrow = T)
+ })
> VRmn <- lapply(c(2, 5, 10, 15), allVR, 1000)
```

Plot the results.

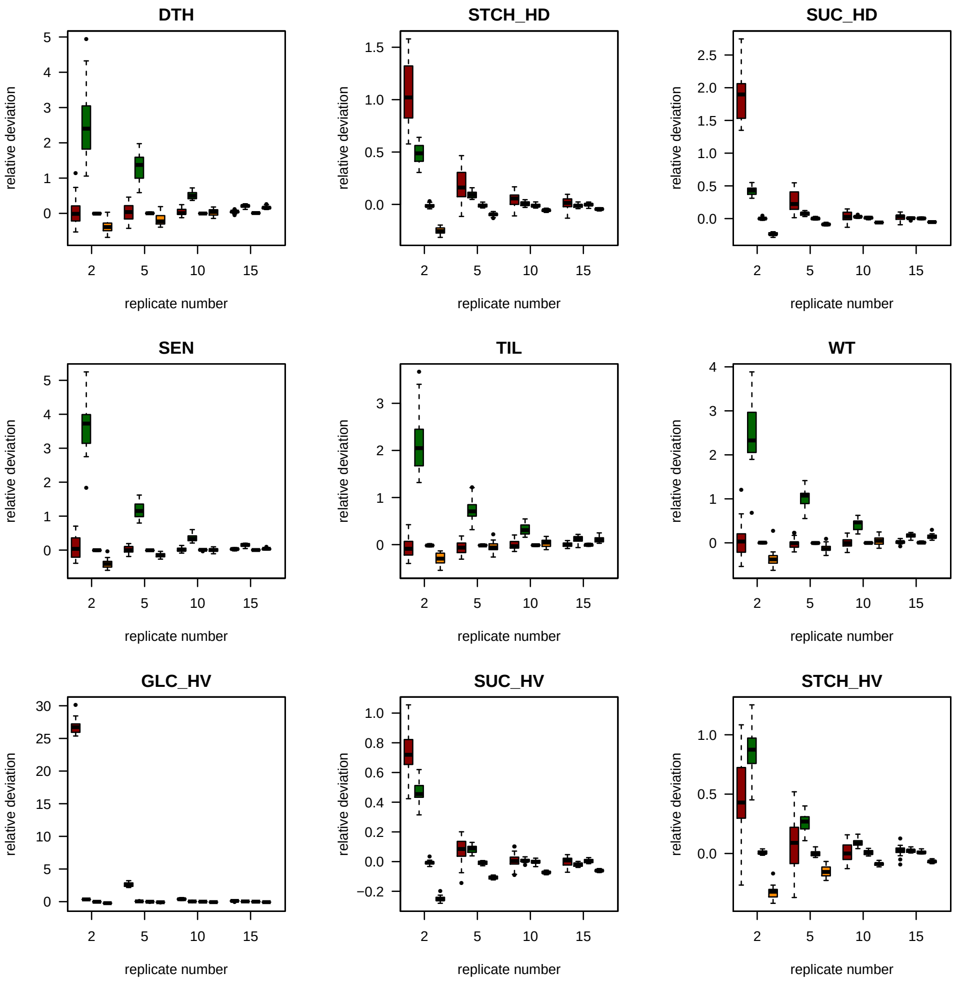

On each plot, relative accuracy distributions for σ_e^2, σ_s^2, σ_a^2 and h^2 are depicted next to each other for each sub-replicate size.

Lastly, I look at the variability of estimates, as for the point estimates.

```
> subVRcv <- cmpfun(function(iSam, rNum, mdl){
+         chn <- lapply(1:5, readChainVR, iSam, rNum, mdl)
+
+         chn <- matrix(array(sapply(chn, t)), nrow = chnLen*nChn, byrow = T)
+         # add heritability
+         idx <- rep(c("e", "s", "a"), each = d)
+         chn <- cbind(chn,
+                 chn[,idx == "a"]/(chn[,idx == "e"] + chn[,idx == "s"] + chn[,idx == "a"]))
+         return(return(apply(chn, 2, sd)/abs(apply(chn, 2, pmode))))
+ })
> allVRcv <- cmpfun(function(rNum, mdl){
+         matrix( array(sapply(1:20, subVRcv, rNum, mdl)),  ncol = d, byrow = T)
+ })
```

```
> VRcv <- lapply(c(2, 5, 10, 15), allVRcv, 1000)
>
```

Now plot the variation metric, scaled by the full-data-derived estimates as before.

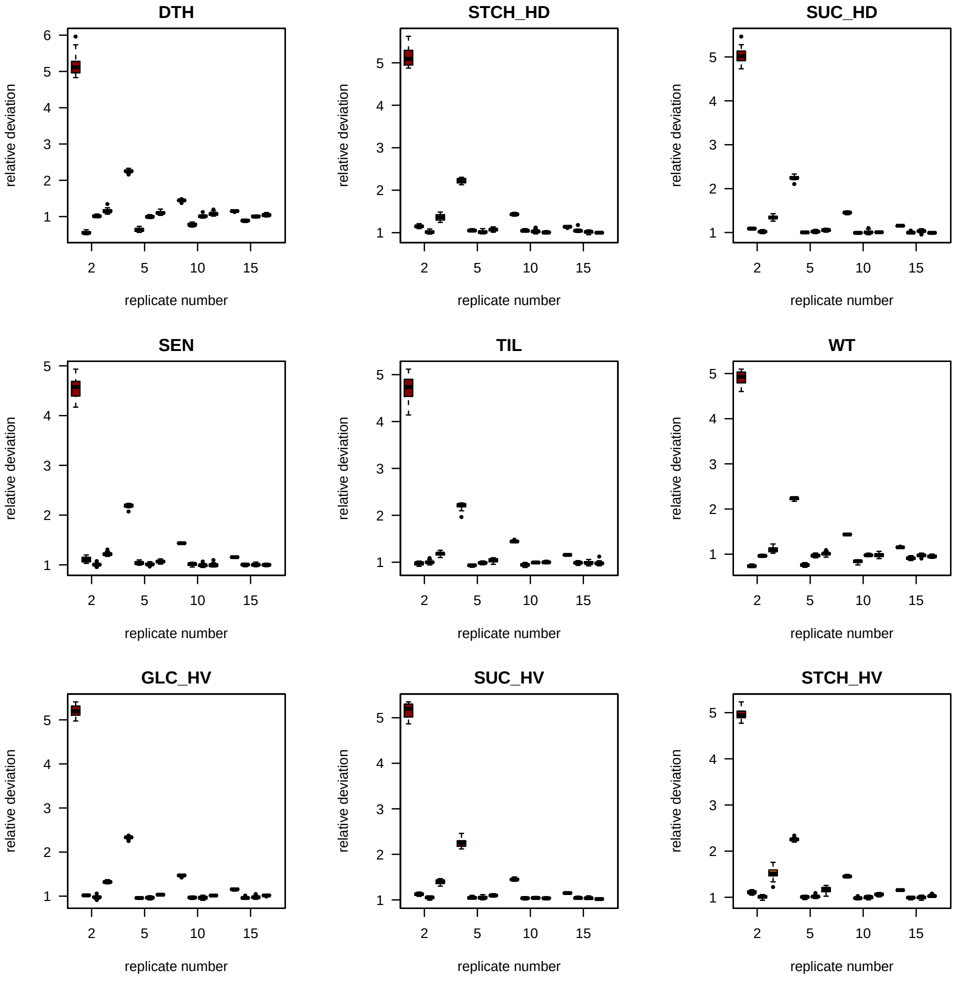

Next I look at accuracy of genetic correlation estimates. I use the correlation between upper triangles of matrices estimated from sub-sampled data and the upper triangle of the matrix derived from the full data set. This is a simple yet adequate measure of matrix similarity, related to the well-known Mantel test. I am only looking at genetic correlations.

```
> subCC <- cmpfun(function(iSam, rNum, mdl){
+         chn <- lapply(1:5, readChain, iSam, rNum, "BV", mdl)
+
+         chn  <- matrix(array(sapply(chn, t)), nrow = chnLen*nChn, byrow = T)
+         gcor <- apply(chn, 1, sampCor)
+         return(apply(gcor, 1, pmode))
+ })
> allCC <- cmpfun(function(rNum, mdl){
```

```
+         array(sapply(1:20, subCC, rNum, mdl))
+ })
> BVcc <- sapply(c(2, 5, 10, 15), allCC, 1000)
```

Now plot the results.

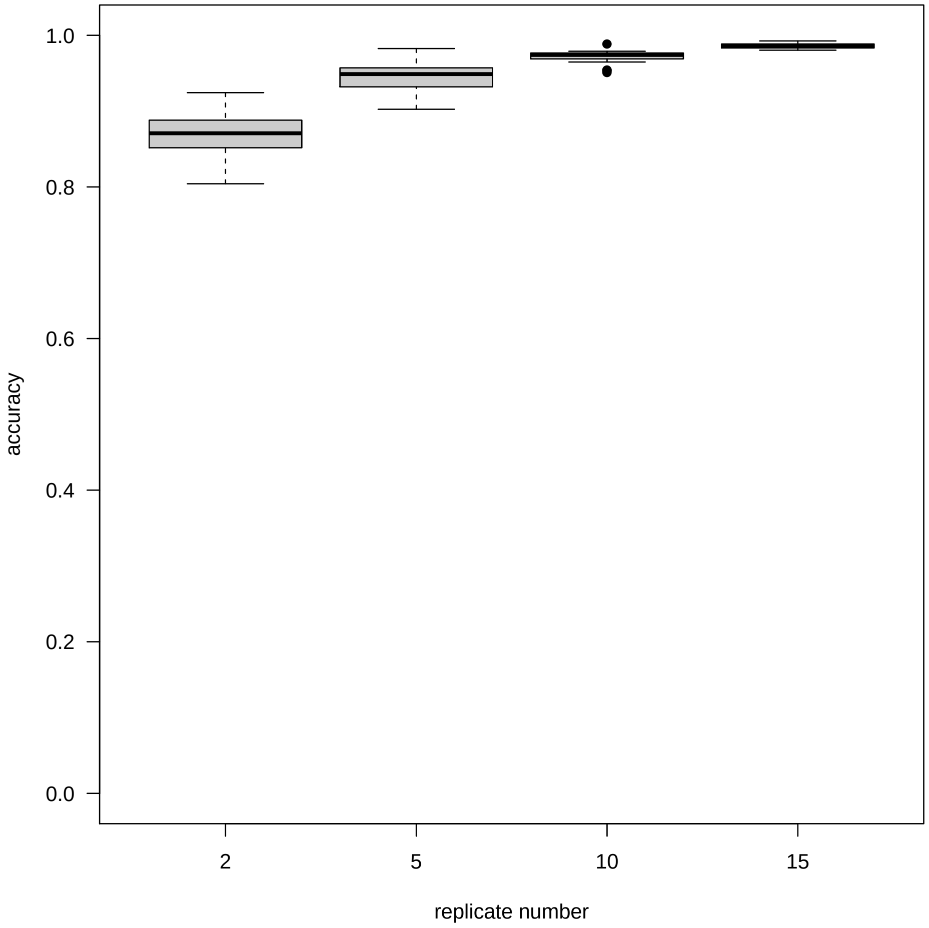

2.2 Model with Student-t errors

I perform exactly the same analyses with estimates from the Student-t model. I am using 3 degrees of freedom for maximally robust inference, an approach that proved successful in numerous simulations and real-data applications.

I give fewer comments to save space, as the analyses are exactly the same as for the Gaussian model.

```
> chn1 <- matrix(.C("GSLmatLoad",
+         "NSCchains3/LN_20_1_3_1.gbin",
+         as.integer(chnLen), as.integer(lnDim), out = double(chnLen*lnDim))$out,
+                 nrow = chnLen, byrow = T)
```

```
> chn2 <- matrix(.C("GSLmatLoad",
+         "NSCchains3/LN_20_1_3_2.gbin",
+         as.integer(chnLen), as.integer(lnDim), out = double(chnLen*lnDim))$out,
+                 nrow = chnLen, byrow = T)
> chn3 <- matrix(.C("GSLmatLoad",
+         "NSCchains3/LN_20_1_3_3.gbin",
+         as.integer(chnLen), as.integer(lnDim), out = double(chnLen*lnDim))$out,
+                 nrow = chnLen, byrow = T)
> chn4 <- matrix(.C("GSLmatLoad",
+         "NSCchains3/LN_20_1_3_4.gbin",
+         as.integer(chnLen), as.integer(lnDim), out = double(chnLen*lnDim))$out,
+                 nrow = chnLen, byrow = T)
> chn5 <- matrix(.C("GSLmatLoad",
+         "NSCchains3/LN_20_1_3_5.gbin",
+         as.integer(chnLen), as.integer(lnDim), out = double(chnLen*lnDim))$out,
+                 nrow = chnLen, byrow = T)
>
```

Now I plot the Gelman-Rubin convergence statistics for each trait. The function to calculate statistic follows.

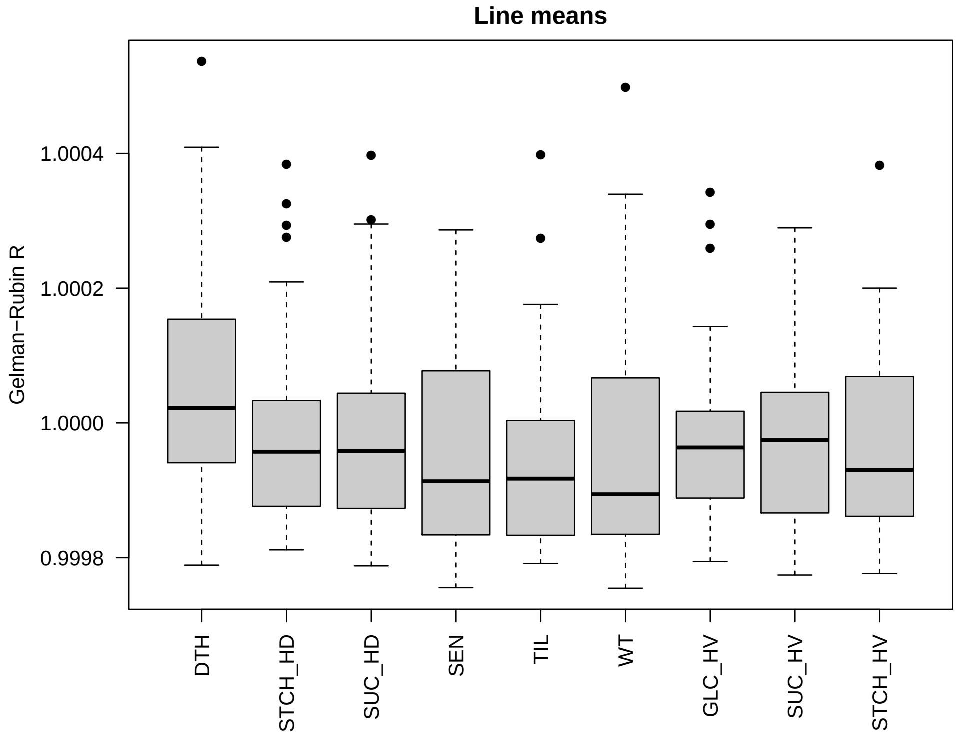

Values smaller than 1.2 reflect complete convergence. I proceed to create a matrix of line mean point estimates, and a matrix of their MCMC relative errors.

```
> tmpMat       <- rbind(chn1, chn2, chn3, chn4, chn5)
> fullLNmatT   <- matrix(apply(tmpMat, 2, pmode), ncol = d, byrow = T)
```

```
> fullLNmatTcv <- matrix(apply(tmpMat, 2, sd), ncol = d, byrow = T)/abs(fullLNmatT)
> colnames(fullLNmatT)   <- trt.list
> colnames(fullLNmatTcv) <- trt.list
```

Next, I move to genome-estimated breeding values (GEBV).

```
> chn1 <- matrix(.C("GSLmatLoad",
+         "NSCchains3/BV_20_1_3_1.gbin",
+         as.integer(chnLen), as.integer(lnDim), out = double(chnLen*lnDim))$out,
+                 nrow = chnLen, byrow = T)
> chn2 <- matrix(.C("GSLmatLoad",
+         "NSCchains3/BV_20_1_3_2.gbin",
+         as.integer(chnLen), as.integer(lnDim), out = double(chnLen*lnDim))$out,
+                 nrow = chnLen, byrow = T)
> chn3 <- matrix(.C("GSLmatLoad",
+         "NSCchains3/BV_20_1_3_3.gbin",
+         as.integer(chnLen), as.integer(lnDim), out = double(chnLen*lnDim))$out,
+                 nrow = chnLen, byrow = T)
> chn4 <- matrix(.C("GSLmatLoad",
+         "NSCchains3/BV_20_1_3_4.gbin",
+         as.integer(chnLen), as.integer(lnDim), out = double(chnLen*lnDim))$out,
+                 nrow = chnLen, byrow = T)
> chn5 <- matrix(.C("GSLmatLoad",
+         "NSCchains3/BV_20_1_3_5.gbin",
+         as.integer(chnLen), as.integer(lnDim), out = double(chnLen*lnDim))$out,
+                 nrow = chnLen, byrow = T)
>
```

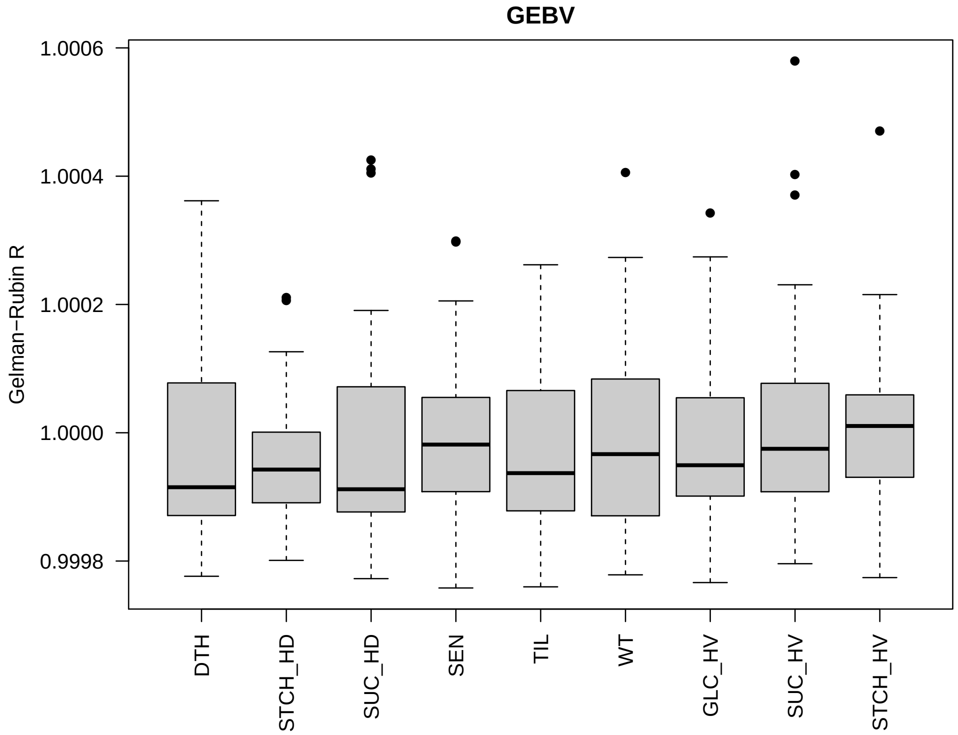

No detectable convergence problems here, either. Make point estimate and error matrices.

```
> tmpMat       <- rbind(chn1, chn2, chn3, chn4, chn5)
> fullBVmatT   <- matrix(apply(tmpMat, 2, pmode), ncol = d, byrow = T)
> fullBVmatTcv <- matrix(apply(tmpMat, 2, sd), ncol = d, byrow = T)/abs(fullBVmatT)
> colnames(fullBVmatT)   <- trt.list
> colnames(fullBVmatTcv) <- trt.list
```

Finally, I look at covariance matrices. We only need the upper triangles.

```
> chn1 <- matrix(.C("GSLmatLoad",
+         "NSCchains3/SigE_20_1_3_1.gbin",
+         as.integer(chnLen), as.integer(d^2), out = double(chnLen*d^2))$out,
+                 nrow = chnLen, byrow = T)[,upInd]
> chn1 <- cbind(chn1,
+         matrix(.C("GSLmatLoad",
+         "NSCchains3/SigS_20_1_3_1.gbin",
+         as.integer(chnLen), as.integer(d^2), out = double(chnLen*d^2))$out,
+                 nrow = chnLen, byrow = T)[,upInd])
> chn1 <- cbind(chn1,
+         matrix(.C("GSLmatLoad",
+         "NSCchains3/SigA_20_1_3_1.gbin",
+         as.integer(chnLen), as.integer(d^2), out = double(chnLen*d^2))$out,
+                 nrow = chnLen, byrow = T)[,upInd])
```

```
> chn2 <- matrix(.C("GSLmatLoad",
+         "NSCchains3/SigE_20_1_3_2.gbin",
+         as.integer(chnLen), as.integer(d^2), out = double(chnLen*d^2))$out,
+                 nrow = chnLen, byrow = T)[,upInd]
> chn2 <- cbind(chn2,
+         matrix(.C("GSLmatLoad",
+         "NSCchains3/SigS_20_1_3_2.gbin",
+         as.integer(chnLen), as.integer(d^2), out = double(chnLen*d^2))$out,
+                 nrow = chnLen, byrow = T)[,upInd])
> chn2 <- cbind(chn2,
+         matrix(.C("GSLmatLoad",
+         "NSCchains3/SigA_20_1_3_2.gbin",
+         as.integer(chnLen), as.integer(d^2), out = double(chnLen*d^2))$out,
+                 nrow = chnLen, byrow = T)[,upInd])
> chn3 <- matrix(.C("GSLmatLoad",
+         "NSCchains3/SigE_20_1_3_3.gbin",
+         as.integer(chnLen), as.integer(d^2), out = double(chnLen*d^2))$out,
+                 nrow = chnLen, byrow = T)[,upInd]
> chn3 <- cbind(chn3,
+         matrix(.C("GSLmatLoad",
+         "NSCchains3/SigS_20_1_3_3.gbin",
+         as.integer(chnLen), as.integer(d^2), out = double(chnLen*d^2))$out,
+                 nrow = chnLen, byrow = T)[,upInd])
> chn3 <- cbind(chn3,
+         matrix(.C("GSLmatLoad",
+         "NSCchains3/SigA_20_1_3_3.gbin",
+         as.integer(chnLen), as.integer(d^2), out = double(chnLen*d^2))$out,
+                 nrow = chnLen, byrow = T)[,upInd])
> chn4 <- matrix(.C("GSLmatLoad",
+         "NSCchains3/SigE_20_1_3_4.gbin",
+         as.integer(chnLen), as.integer(d^2), out = double(chnLen*d^2))$out,
+                 nrow = chnLen, byrow = T)[,upInd]
> chn4 <- cbind(chn4,
+         matrix(.C("GSLmatLoad",
+         "NSCchains3/SigS_20_1_3_4.gbin",
+         as.integer(chnLen), as.integer(d^2), out = double(chnLen*d^2))$out,
+                 nrow = chnLen, byrow = T)[,upInd])
> chn4 <- cbind(chn4,
+         matrix(.C("GSLmatLoad",
+         "NSCchains3/SigA_20_1_3_4.gbin",
+         as.integer(chnLen), as.integer(d^2), out = double(chnLen*d^2))$out,
+                 nrow = chnLen, byrow = T)[,upInd])
> chn5 <- matrix(.C("GSLmatLoad",
+         "NSCchains3/SigE_20_1_3_5.gbin",
+         as.integer(chnLen), as.integer(d^2), out = double(chnLen*d^2))$out,
```

```
+                  nrow = chnLen, byrow = T)[,upInd]
> chn5 <- cbind(chn5,
+         matrix(.C("GSLmatLoad",
+         "NSCchains3/SigS_20_1_3_5.gbin",
+         as.integer(chnLen), as.integer(d^2), out = double(chnLen*d^2))$out,
+                  nrow = chnLen, byrow = T)[,upInd])
> chn5 <- cbind(chn5,
+         matrix(.C("GSLmatLoad",
+         "NSCchains3/SigA_20_1_3_5.gbin",
+         as.integer(chnLen), as.integer(d^2), out = double(chnLen*d^2))$out,
+                  nrow = chnLen, byrow = T)[,upInd])
```

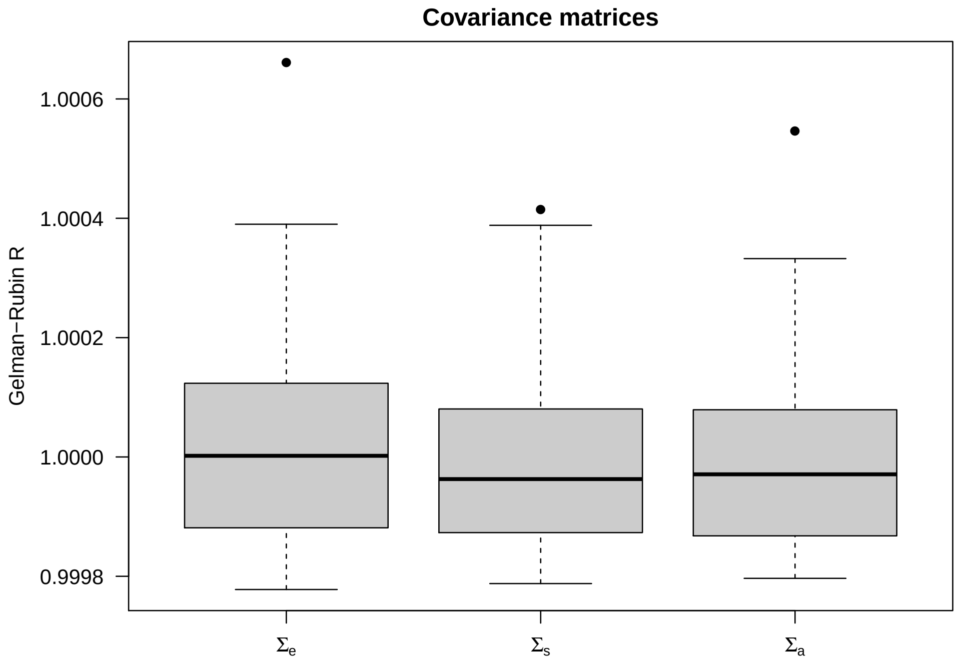

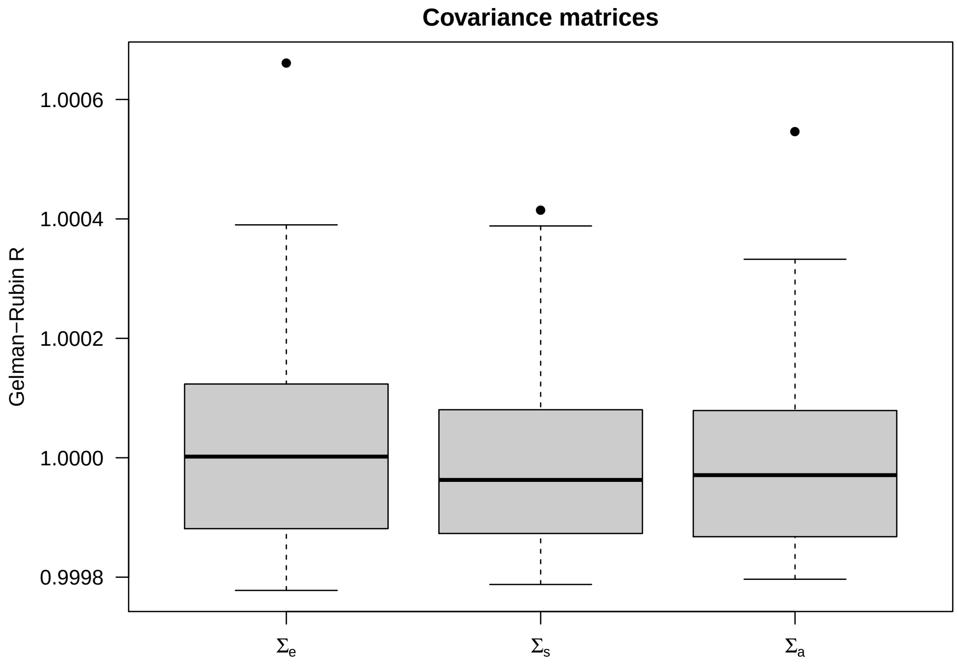

No discernible convergence problems. To make sure, I plot Markov chains for variances, starting from σ_e^2.

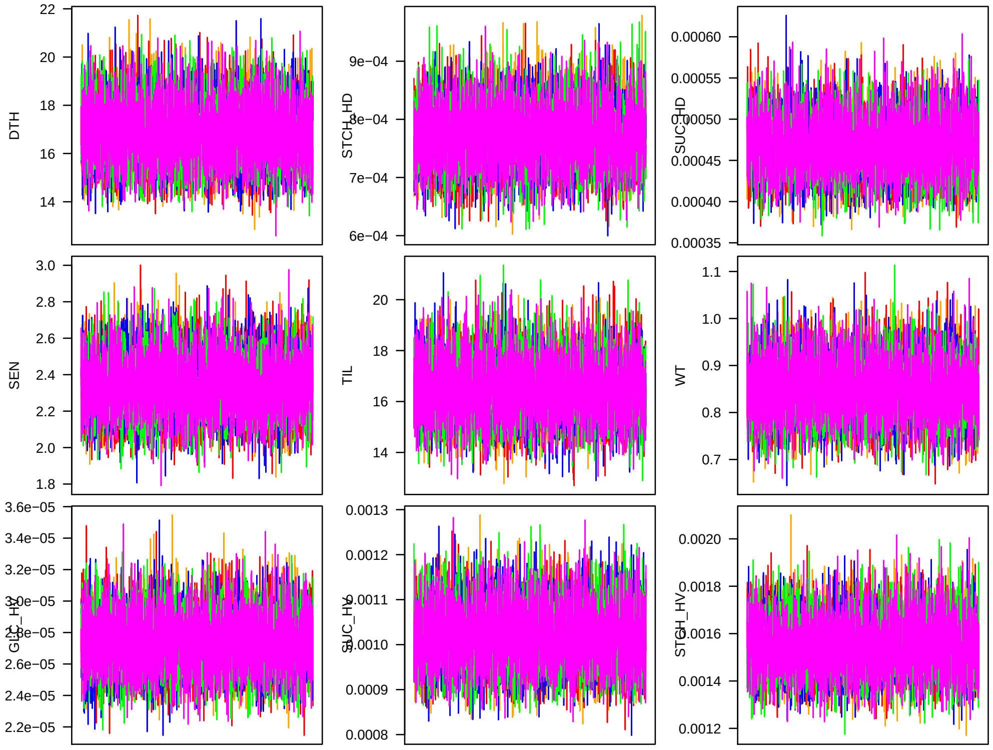

Then the non-additive variances.

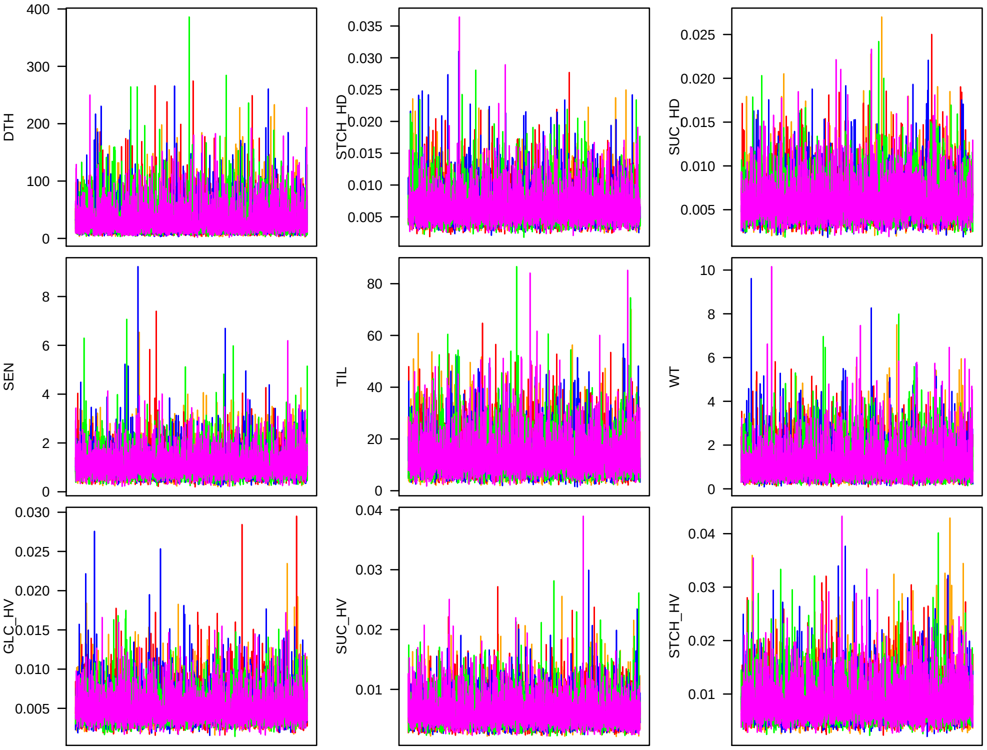

Finally, the additive.

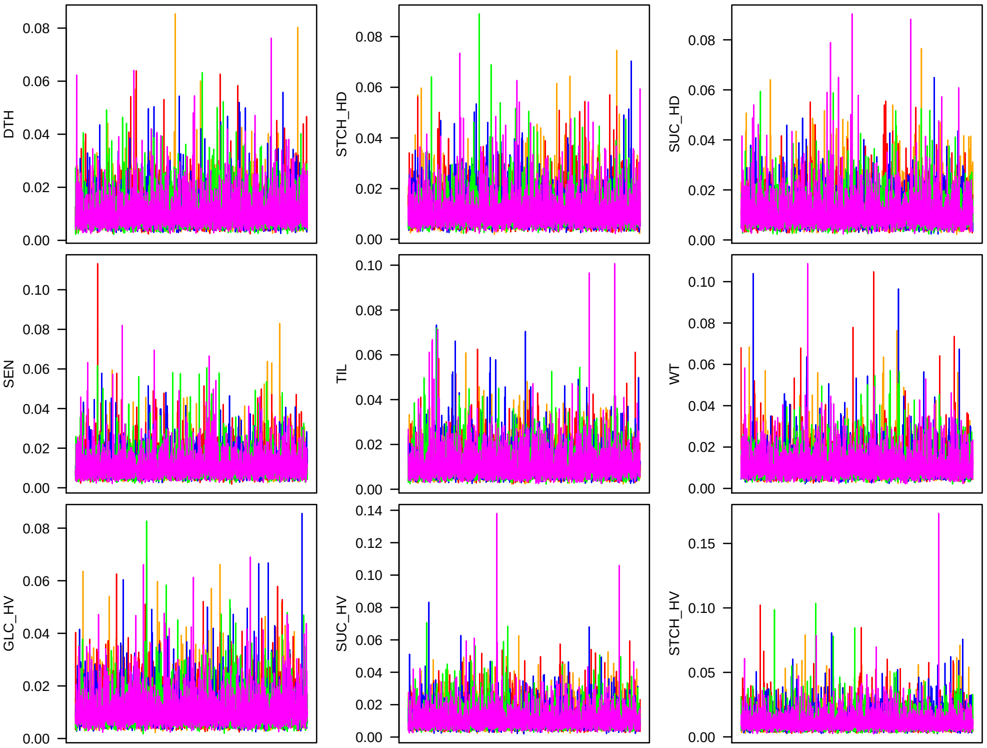

```
> tmpMat <- rbind(chn1, chn2, chn3, chn4, chn5)
> hrMat  <- tmpMat[,levFac == "a"][,diagInd]/(
+          tmpMat[, levFac == "a"][, diagInd] + tmpMat[, levFac == "s"][,diagInd]
+                  + tmpMat[, levFac == "e"][,diagInd]
+          )
> fullHvecT             <- apply(hrMat, 2, pmode)
> names(fullHvecT)      <- trt.list
> fullHvecTci           <- apply(hrMat, 2, quantile, c(0.025, 0.975))
> colnames(fullHvecTci) <- trt.list
> fullHvecTcv           <- apply(hrMat, 2, sd)/abs(fullHvecT)
> names(fullHvecTcv)    <- trt.list
```

Individual variances are:

```
> fullEvecT             <- apply(tmpMat[,levFac == "e"][,diagInd], 2, pmode)
> names(fullEvecT)      <- trt.list
> fullEvecTci           <- apply(tmpMat[,levFac == "e"][,diagInd], 2,
+                                                       quantile, c(0.025, 0.975))
> colnames(fullEvecTci) <- trt.list
> fullEvecTcv           <- apply(tmpMat[,levFac == "e"][,diagInd], 2, sd)/abs(fullEvecT)
> names(fullEvecTcv)    <- trt.list
> fullSvecT             <- apply(tmpMat[,levFac == "s"][,diagInd], 2, pmode)
> names(fullSvecT)      <- trt.list
> fullSvecTci           <- apply(tmpMat[,levFac == "s"][,diagInd], 2,
+                                                       quantile, c(0.025, 0.975))
> colnames(fullSvecTci) <- trt.list
> fullSvecTcv           <- apply(tmpMat[,levFac == "s"][,diagInd], 2, sd)/abs(fullSvecT)
> names(fullSvecTcv)    <- trt.list
> fullAvecT             <- apply(tmpMat[,levFac == "a"][,diagInd], 2, pmode)
> names(fullAvecT)      <- trt.list
> fullAvecTci           <- apply(tmpMat[,levFac == "a"][,diagInd], 2,
+                                                       quantile, c(0.025, 0.975))
> colnames(fullAvecTci) <- trt.list
> fullAvecTcv           <- apply(tmpMat[,levFac == "a"][,diagInd], 2, sd)/abs(fullAvecT)
> names(fullAvecTcv)    <- trt.list
> round(fullHvecT,4)

    DTH STCH_HD  SUC_HD     SEN     TIL      WT  GLC_HV  SUC_HV STCH_HV
 0.0001  0.5805  0.6147  0.0022  0.0002  0.0038  0.6906  0.5823  0.5072


> round(fullHvecTci,4)

        DTH STCH_HD SUC_HD    SEN   TIL     WT GLC_HV SUC_HV STCH_HV
2.5%  1e-04  0.2983 0.3305 0.0011 1e-04 0.0016 0.3727 0.3098  0.2576
97.5% 7e-04  0.8207 0.8421 0.0084 1e-03 0.0169 0.8759 0.8266  0.7901
```

```
> round(fullEvecT,4)

    DTH STCH_HD  SUC_HD     SEN     TIL      WT  GLC_HV  SUC_HV STCH_HV
16.7704  0.0008  0.0005  2.3073 16.4319  0.8413  0.0000  0.0010  0.0015


> round(fullEvecTci,4)

          DTH STCH_HD SUC_HD    SEN     TIL     WT GLC_HV SUC_HV STCH_HV
2.5%  14.5337   7e-04  4e-04 2.0379 14.3018 0.7321      0 0.0009  0.0013
97.5% 19.4399   9e-04  5e-04 2.6719 18.8801 0.9743      0 0.0012  0.0018


> round(fullSvecT,4)

    DTH STCH_HD  SUC_HD     SEN     TIL      WT  GLC_HV  SUC_HV STCH_HV
17.2522  0.0054  0.0048  0.8445  9.3458  0.6907  0.0042  0.0050  0.0066


> round(fullSvecTci,4)

           DTH STCH_HD SUC_HD    SEN     TIL     WT GLC_HV SUC_HV STCH_HV
2.5%    6.3002  0.0032 0.0029 0.4081  4.0415 0.2703 0.0025  0.003  0.0038
97.5% 108.3572  0.0138 0.0121 2.5647 33.8054 3.3272 0.0107  0.013  0.0181


> round(fullAvecT,4)

    DTH STCH_HD  SUC_HD     SEN     TIL      WT  GLC_HV  SUC_HV STCH_HV
 0.0071  0.0075  0.0073  0.0073  0.0073  0.0075  0.0074  0.0074  0.0080


> round(fullAvecTci,4)

         DTH STCH_HD SUC_HD    SEN    TIL     WT GLC_HV SUC_HV STCH_HV
2.5%  0.0039  0.0040 0.0039 0.0039 0.0039 0.0039 0.0039 0.0040  0.0041
97.5% 0.0263  0.0275 0.0274 0.0282 0.0281 0.0280 0.0277 0.0287  0.0308
```

Now I save the values and their credible intervals to a file.

```
> cat("trait_name\th^2\tsigma^2_e\tsigma^2_s\tsigma^2_e",
+         apply(
+                 cbind(trt.list, rep("\t", d),
+                         signif(fullHvecT,3),
+                         rep(" (", d), signif(fullHvecTci[1,],3),
+                         rep(", ", d), signif(fullHvecTci[2,],3), rep(")\t", d),
+                         signif(fullEvecT,3),
+                         rep(" (", d), signif(fullEvecTci[1,],3),
+                         rep(", ", d), signif(fullEvecTci[2,],3), rep(")\t", d),
+                         signif(fullSvecT,3),
```

```
+                          rep(" (", d), signif(fullSvecTci[1,],3),
+                          rep(", ", d), signif(fullSvecTci[2,],3), rep(")\t", d),
+                          signif(fullAvecT,3),
+                          rep(" (", d), signif(fullAvecTci[1,],3),
+                          rep(", ", d), signif(fullAvecTci[2,],3), rep(")\t", d)
+                          ),
+                  1, paste, collapse=""),
+                  file = "variancesStudentT.tsv", sep = "\n")
>
```

I now look at genetic correlations from the $\mathbf{\Sigma}_e$ and $\mathbf{\Sigma}_a$ matrices.

```
> eCorMCMC <- apply(tmpMat[,levFac == "e"], 1, cov2corTri)
> eCorMnT   <- apply(eCorMCMC, 1, pmode)
> eCorMnTci <- apply(eCorMCMC, 1, quantile, c(0.025, 0.975))
>
```

I calculate point estimates and 95% credible intervals for each correlation value.

```
> chn1 <- matrix(.C("GSLmatLoad",
+         "NSCchains1000/BV_20_1_1000_1.gbin",
+         as.integer(chnLen), as.integer(lnDim), out = double(chnLen*lnDim))$out,
+                 nrow = chnLen, byrow = T)
> chn2 <- matrix(.C("GSLmatLoad",
+         "NSCchains1000/BV_20_1_1000_2.gbin",
+         as.integer(chnLen), as.integer(lnDim), out = double(chnLen*lnDim))$out,
+                 nrow = chnLen, byrow = T)
> chn3 <- matrix(.C("GSLmatLoad",
+         "NSCchains1000/BV_20_1_1000_3.gbin",
+         as.integer(chnLen), as.integer(lnDim), out = double(chnLen*lnDim))$out,
+                 nrow = chnLen, byrow = T)
> chn4 <- matrix(.C("GSLmatLoad",
+         "NSCchains1000/BV_20_1_1000_4.gbin",
+         as.integer(chnLen), as.integer(lnDim), out = double(chnLen*lnDim))$out,
+                 nrow = chnLen, byrow = T)
> chn5 <- matrix(.C("GSLmatLoad",
+         "NSCchains1000/BV_20_1_1000_5.gbin",
+         as.integer(chnLen), as.integer(lnDim), out = double(chnLen*lnDim))$out,
+                 nrow = chnLen, byrow = T)
> tmpMat       <- rbind(chn1, chn2, chn3, chn4, chn5)
> sampCor <- cmpfun(function(vec){
+         res <- cor(matrix(vec, ncol = d, byrow = T))
+         return(res[upper.tri(res)])
+ })
> corMatT <- matrix(1, d, d)
> colnames(corMatT) <- trt.list
> rownames(corMatT) <- trt.list
```

```
> GCorTmat    <- apply(tmpMat, 1, sampCor)
> fullGCorT   <- apply(GCorTmat, 1, pmode)
> fullGCorTci <- apply(GCorTmat, 1, quantile, c(0.025, 0.975))
> corMatT[upper.tri(corMatT)] <- fullGCorT
> corMatTl <- matrix(1, d, d)
> corMatTl[upper.tri(corMatTl)] <- fullGCorTci[1,]
> corMatTu <- matrix(1, d, d)
> corMatTu[upper.tri(corMatTu)] <- fullGCorTci[2,]
> tmpCmat <- matrix(1, d, d)
> tmpCmat[upper.tri(tmpCmat)]   <- eCorMnT
> corMatT[lower.tri(corMatT)]   <- t(tmpCmat)[lower.tri(tmpCmat)]
> tmpCmat[upper.tri(tmpCmat)]   <- eCorMnTci[1,]
> corMatTl[lower.tri(corMatTl)] <- t(tmpCmat)[lower.tri(tmpCmat)]
> tmpCmat[upper.tri(tmpCmat)]   <- eCorMnTci[2,]
> corMatTu[lower.tri(corMatTu)] <- t(tmpCmat)[lower.tri(tmpCmat)]
> rm(tmpCmat)
> signif(corMatT, 3)
```

```
             DTH   STCH_HD   SUC_HD     SEN     TIL      WT  GLC_HV  SUC_HV STCH_HV
DTH       1.0000  0.468000  0.33300  0.0484  0.4150  0.3370  0.1150  0.2910  0.6170
STCH_HD   0.0054  1.000000  0.19100 -0.0840  0.2220  0.4690  0.0599  0.0777  0.4640
SUC_HD    0.0411  0.229000  1.00000 -0.0188  0.3410  0.2180  0.0883  0.2470  0.1230
SEN       0.0753 -0.037100 -0.00554  1.0000  0.1890 -0.3630 -0.0181 -0.1240 -0.0905
TIL       0.0160  0.068600  0.04480  0.0681  1.0000  0.0606  0.1220  0.1790  0.0417
WT       -0.0253  0.035000  0.08660 -0.1420 -0.1040  1.0000  0.0548  0.3260  0.5090
GLC_HV    0.0754  0.047500  0.09580  0.0521  0.0365  0.0790  1.0000  0.0381  0.0287
SUC_HV    0.0122  0.000381  0.13700 -0.2340  0.0239  0.2150  0.2840  1.0000  0.1110
STCH_HV   0.0330  0.041600  0.09970 -0.1260 -0.0496  0.4920  0.1220  0.3450  1.0000
```

Now save the correlation matrix to a file.

```
> cat(paste(c("trait_names", trt.list), collapse = "\t"),
+         file = "correlationMatrixT.tsv", sep = "\n")
> for (trt in 1:d){
+         cat(paste(c("", signif(corMatTu[trt,], 3)), collapse = "\t"),
+                 file = "correlationMatrixT.tsv", sep = "\n", append = T)
+         cat(paste(c(trt.list[trt], signif(corMatT[trt,], 3)), collapse = "\t"),
+                 file = "correlationMatrixT.tsv", sep = "\n", append = T)
+         cat(paste(c("", signif(corMatTl[trt,], 3)), collapse = "\t"),
+                 file = "correlationMatrixT.tsv", sep = "\n", append = T)
+ }
```

Now I check convergence for the sub-sampled data.

```
> LNgr <- lapply(c(2, 5, 10, 15), allGR, "LN", 3)
> BVgr <- lapply(c(2, 5, 10, 15), allGR, "BV", 3)
```

Now plot the results for line means:

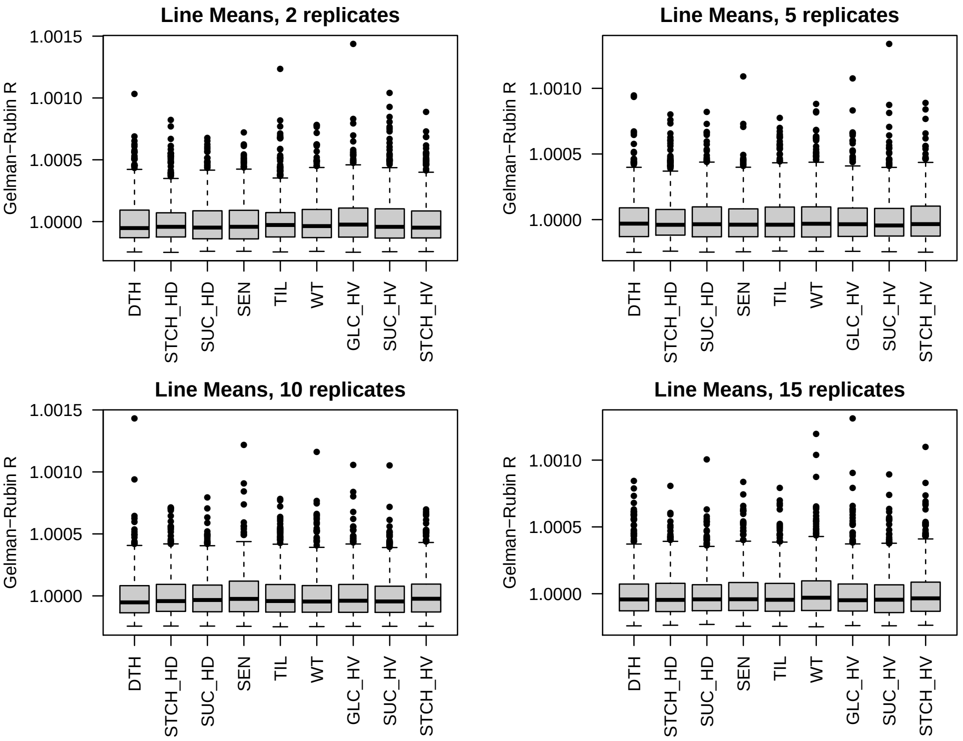

And GEBVs:

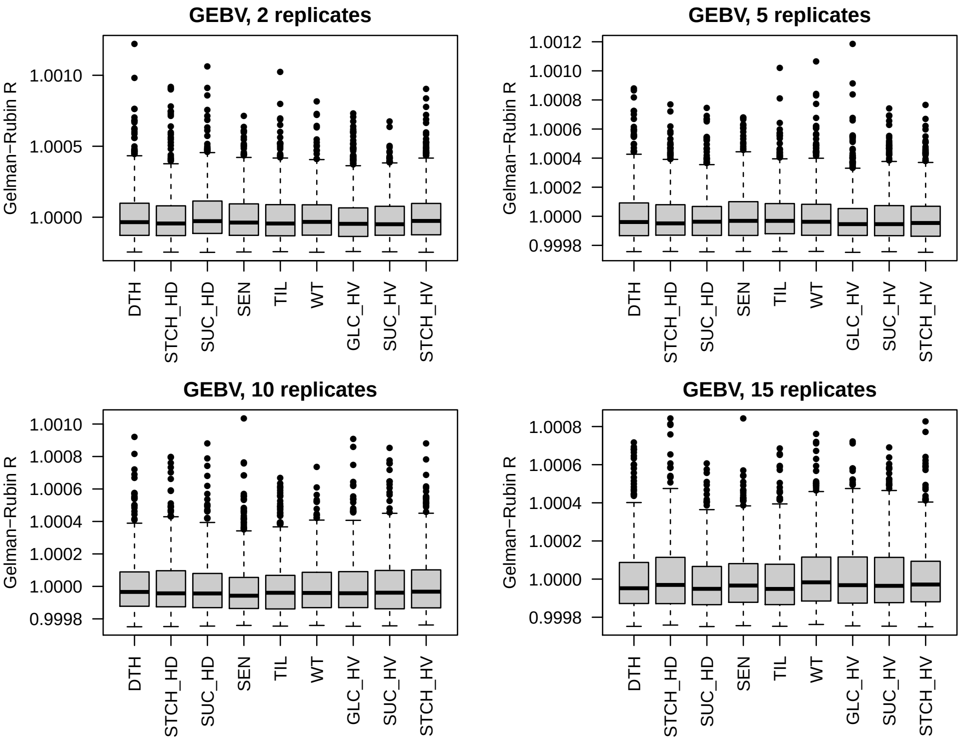

Now I do the same for covariance matrices.

```
> SigGR <- lapply(c(2, 5, 10, 15), allGRvr, 3)
```

Plot.

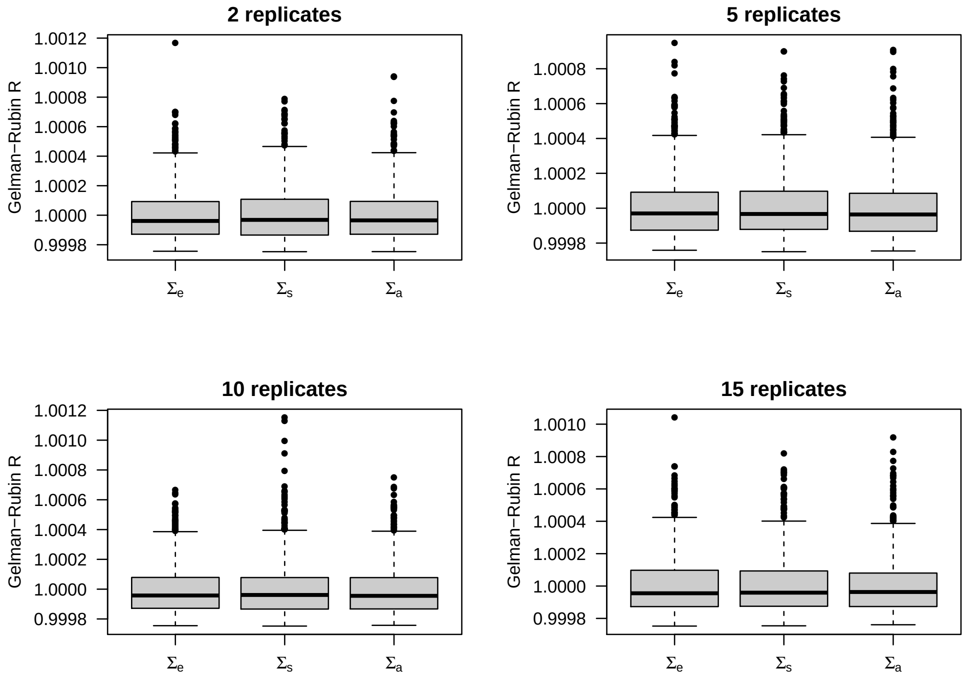

All parameters appear to have converged completely. I can proceed to analyze the results.

```
> LNmnT <- lapply(c(2, 5, 10, 15), allMn, "LN", 3)
> BVmnT <- lapply(c(2, 5, 10, 15), allMn, "BV", 3)
>
```

Plot accuracies (reflected by correlations of point estimates from sub-sampled data with estimates from the whole data set) by trait, comparing replicate sizes directly on the same plot. First, line means.

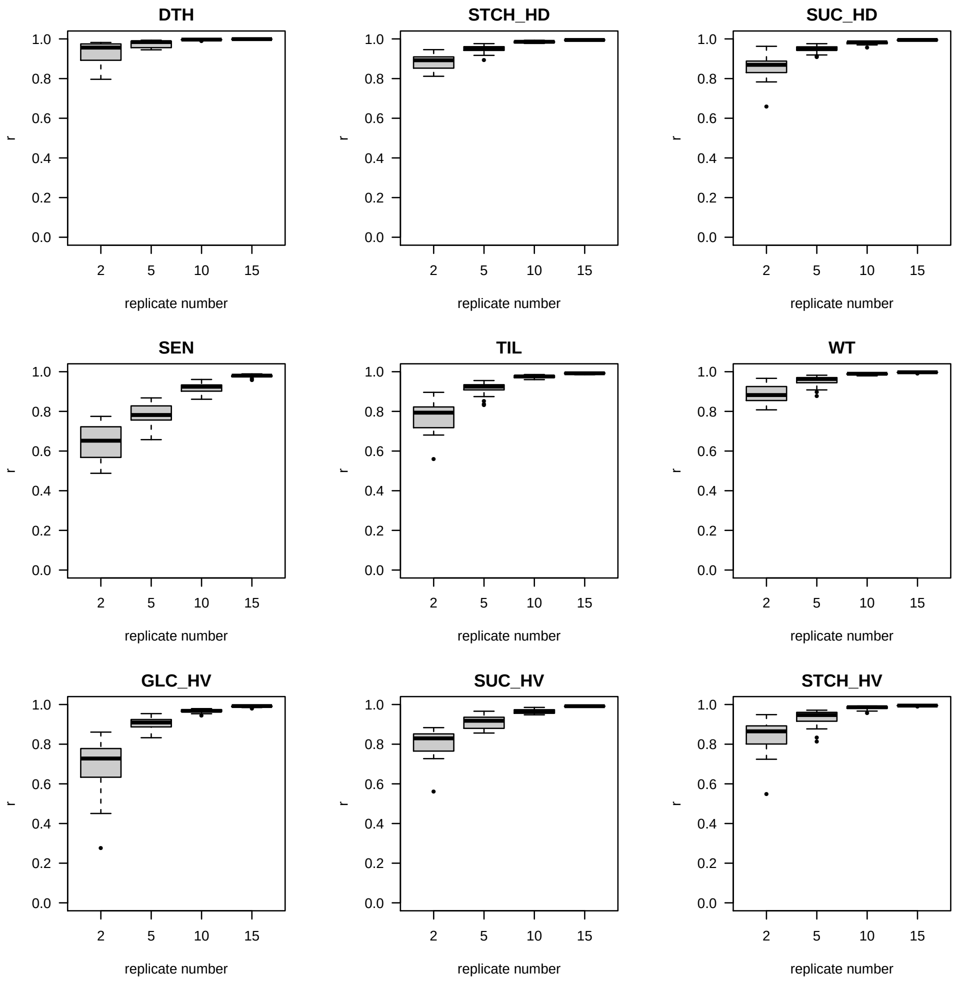
DTH
STCH_HD
SUC_HD
SEN
TIL
WT
GLC_HV
SUC_HV
STCH_HV
r
replicate number
2
5
10
15
0.0
0.2
0.4
0.6
0.8
1.0

Then GEBVs.

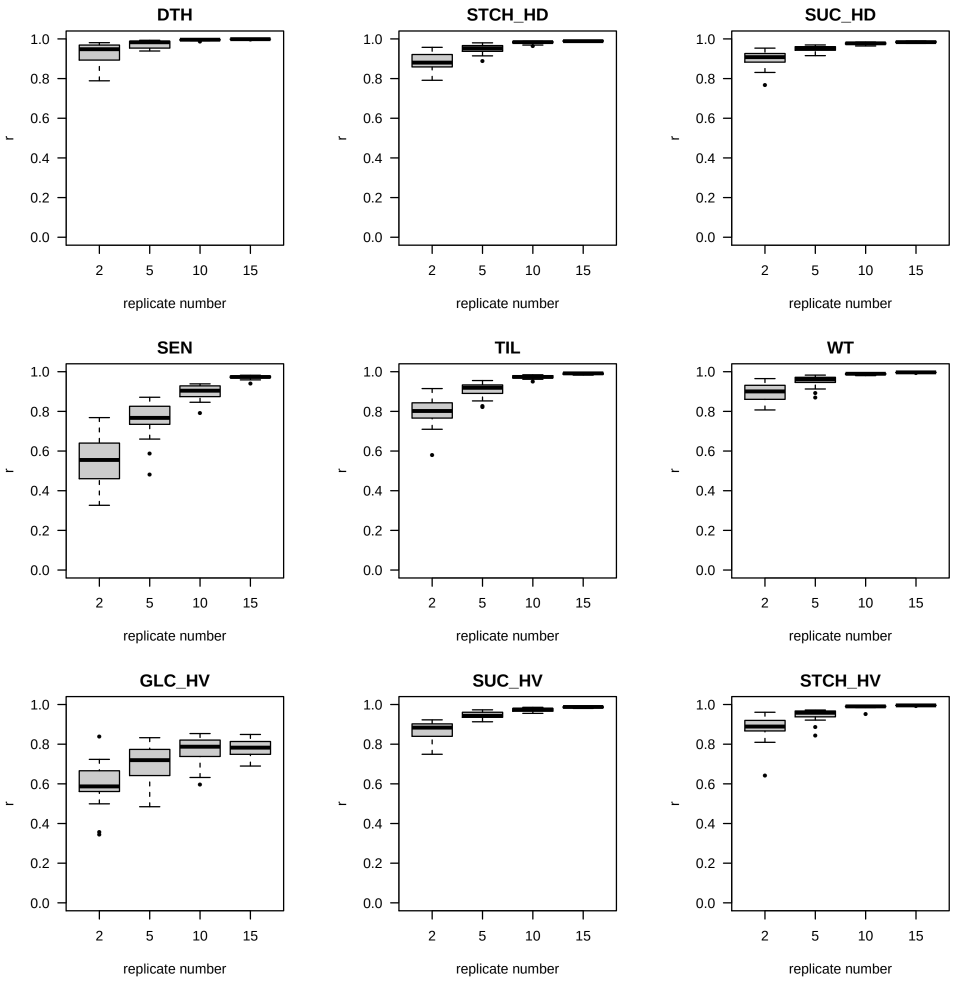

Look at uncertainty of estimates.

```
> LNcvT <- lapply(c(2, 5, 10, 15), allCV, "LN", 3)
> BVcvT <- lapply(c(2, 5, 10, 15), allCV, "BV", 3)
>
```

Plot the values for each replicate number, divided by the CVs for the whole data set. Start with line means.

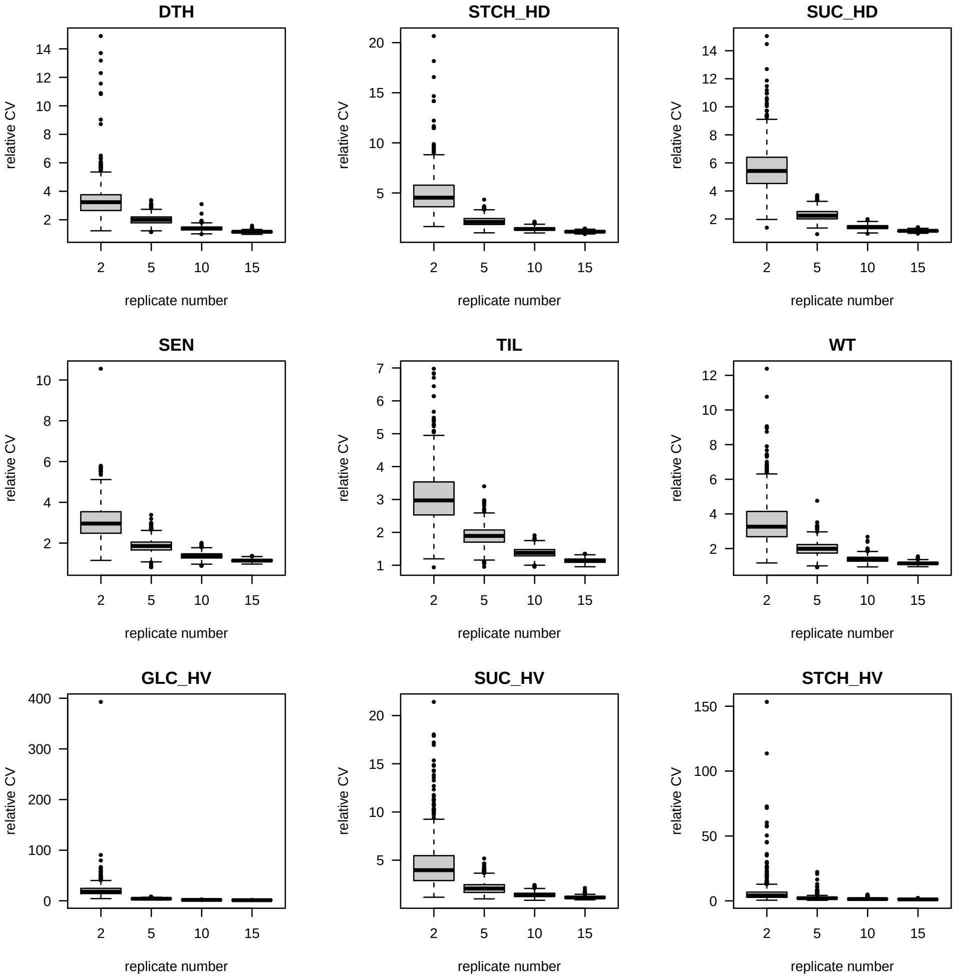
DTH
STCH_HD
SUC_HD
SEN
TIL
WT
GLC_HV
SUC_HV
STCH_HV
relative CV
replicate number
2
5
10
15

And GEBVs:

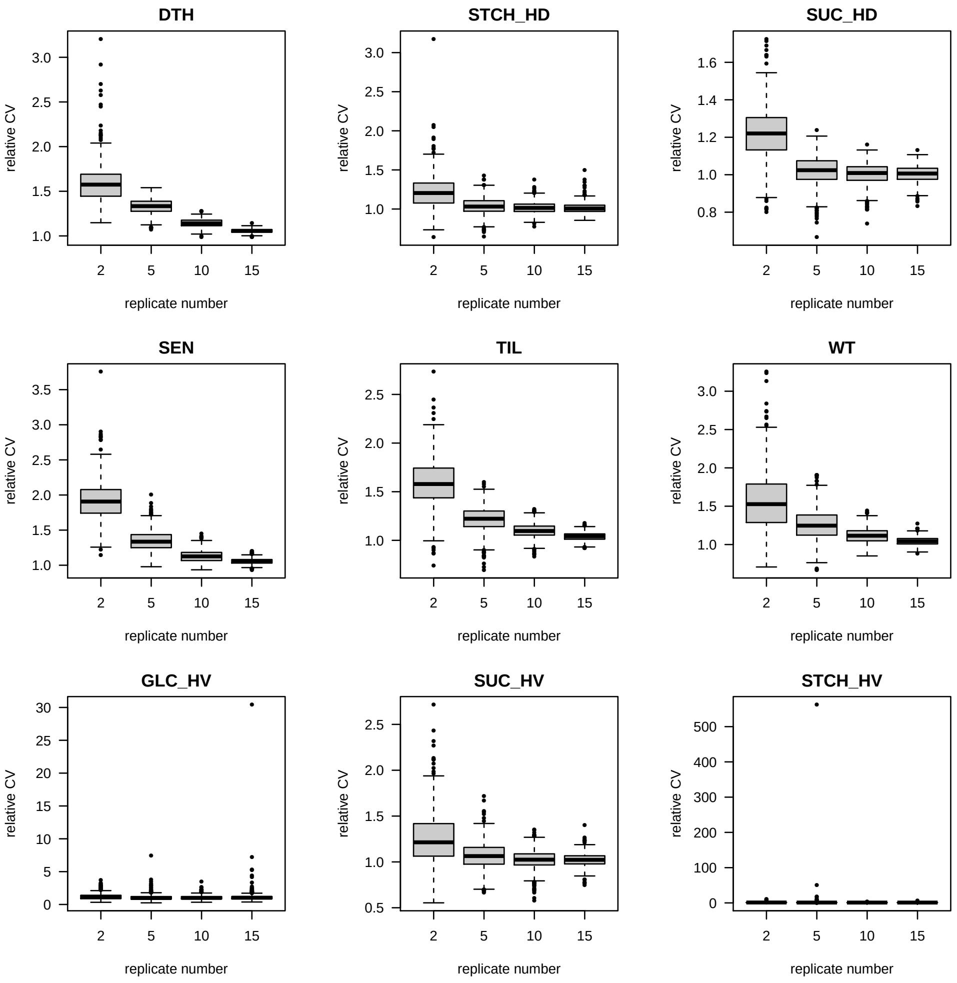

Re-plot without outliers:

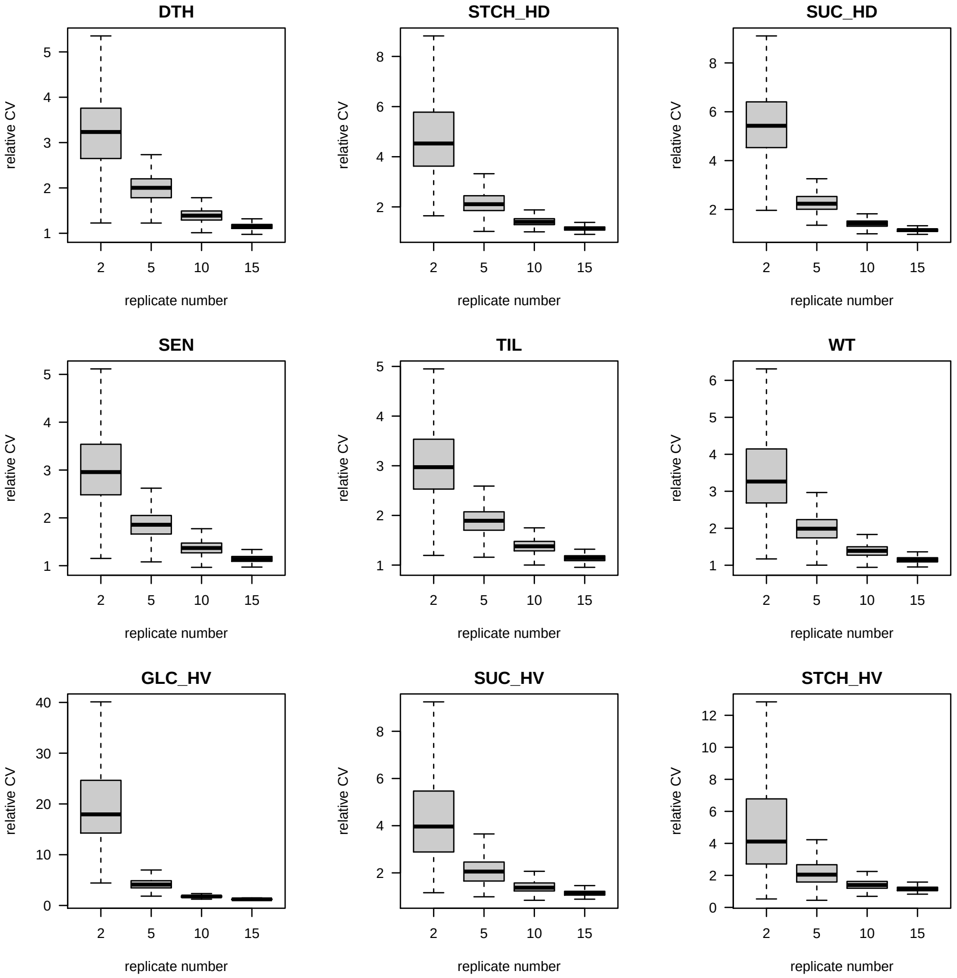
DTH
STCH_HD
SUC_HD
SEN
TIL
WT
GLC_HV
SUC_HV
STCH_HV
relative CV
replicate number

And GEBVs:

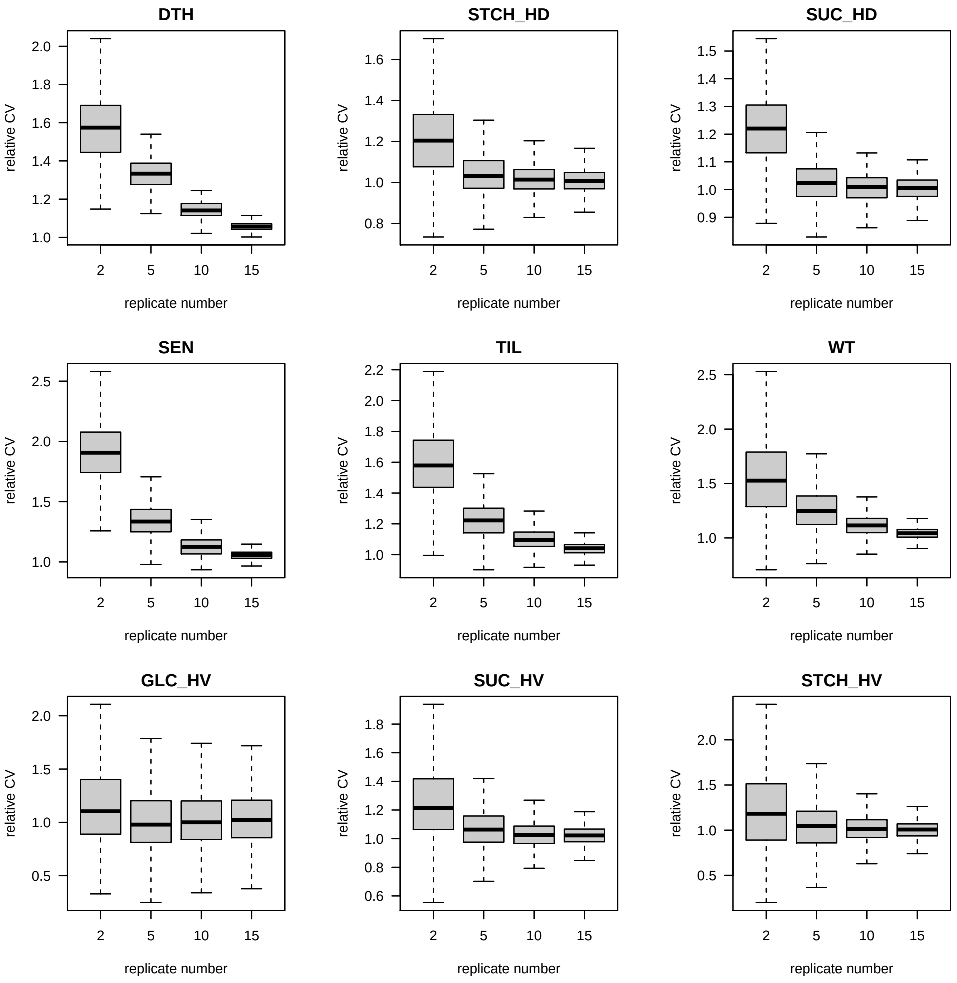

Next, variance and heritability accuracies.

```
> VRmnT <- lapply(c(2, 5, 10, 15), allVR, 3)
```

Plot the results.

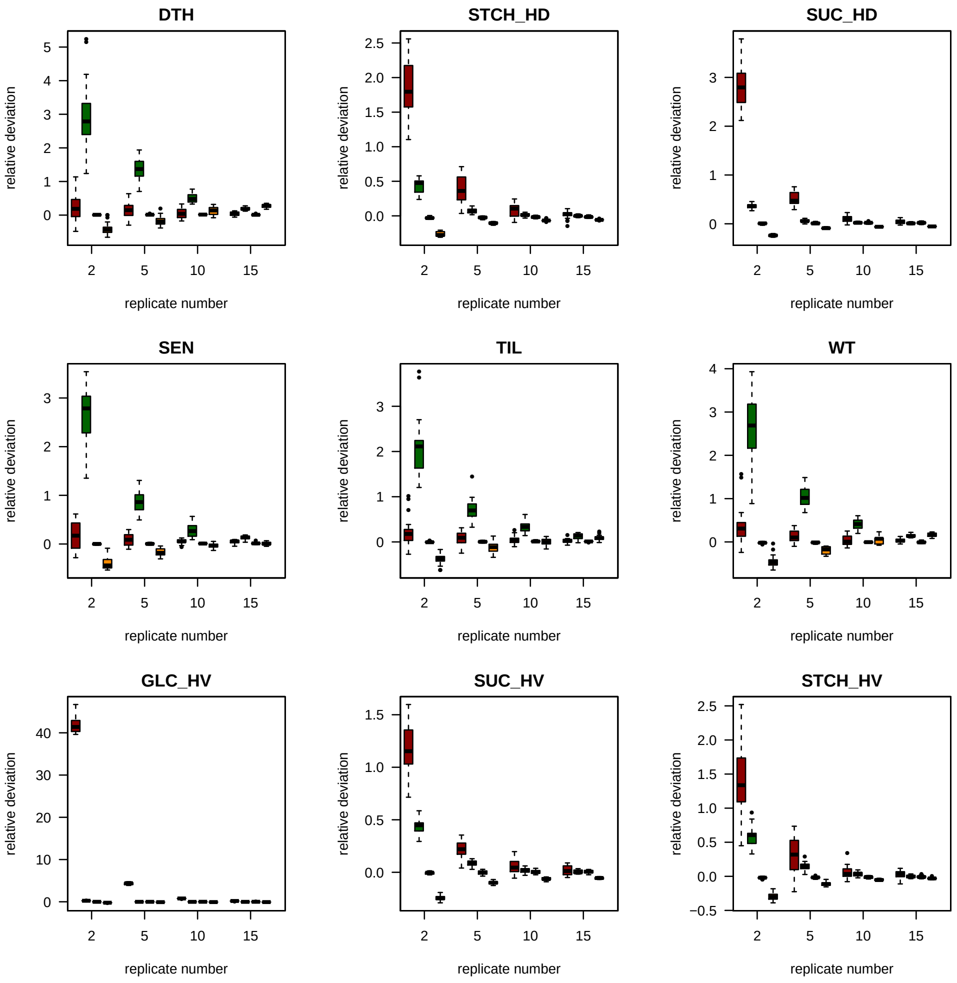

Lastly, I look at the variability of estimates, as for the point estimates.

```
> VRcvT <- lapply(c(2, 5, 10, 15), allVRcv, 3)
>
```

Now plot the variation metric, scaled by the full-data-derived estimates as before.

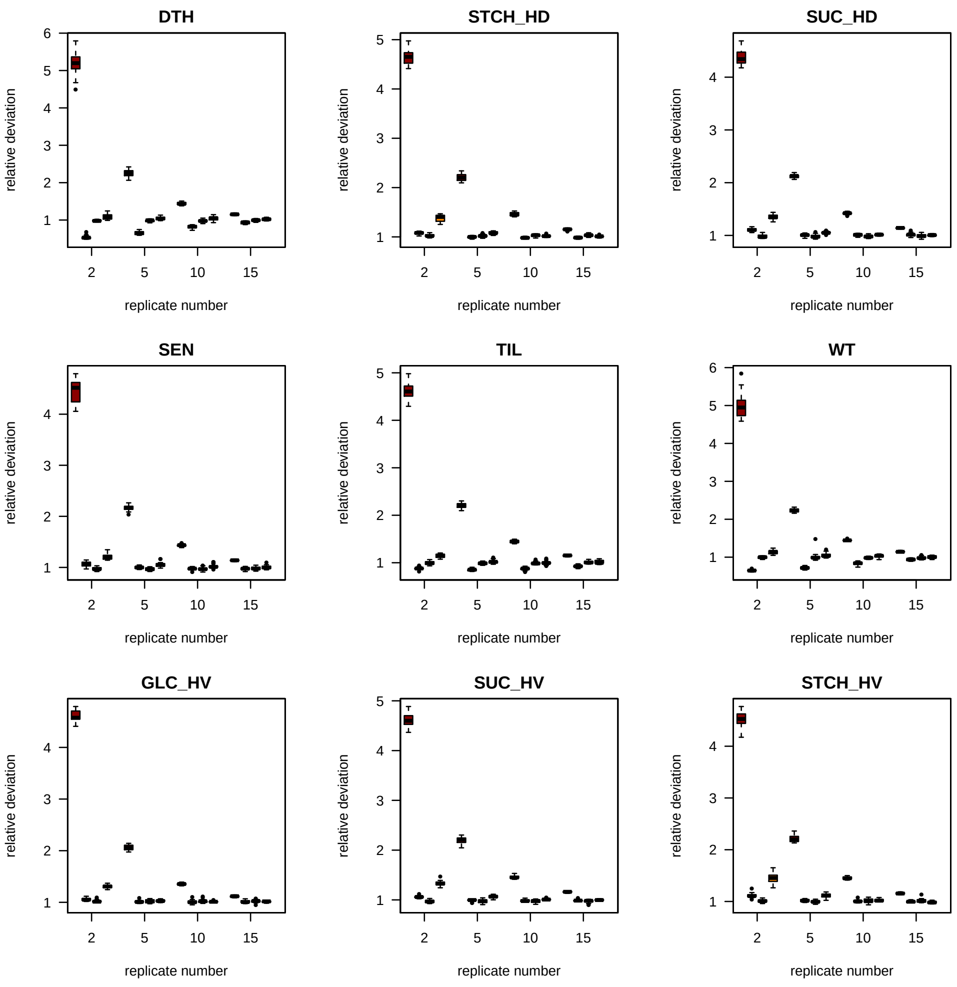

Next I look at accuracy of genetic correlation estimates.

```
> BVccT <- sapply(c(2, 5, 10, 15), allCC, 3)
```

Now plot the results.

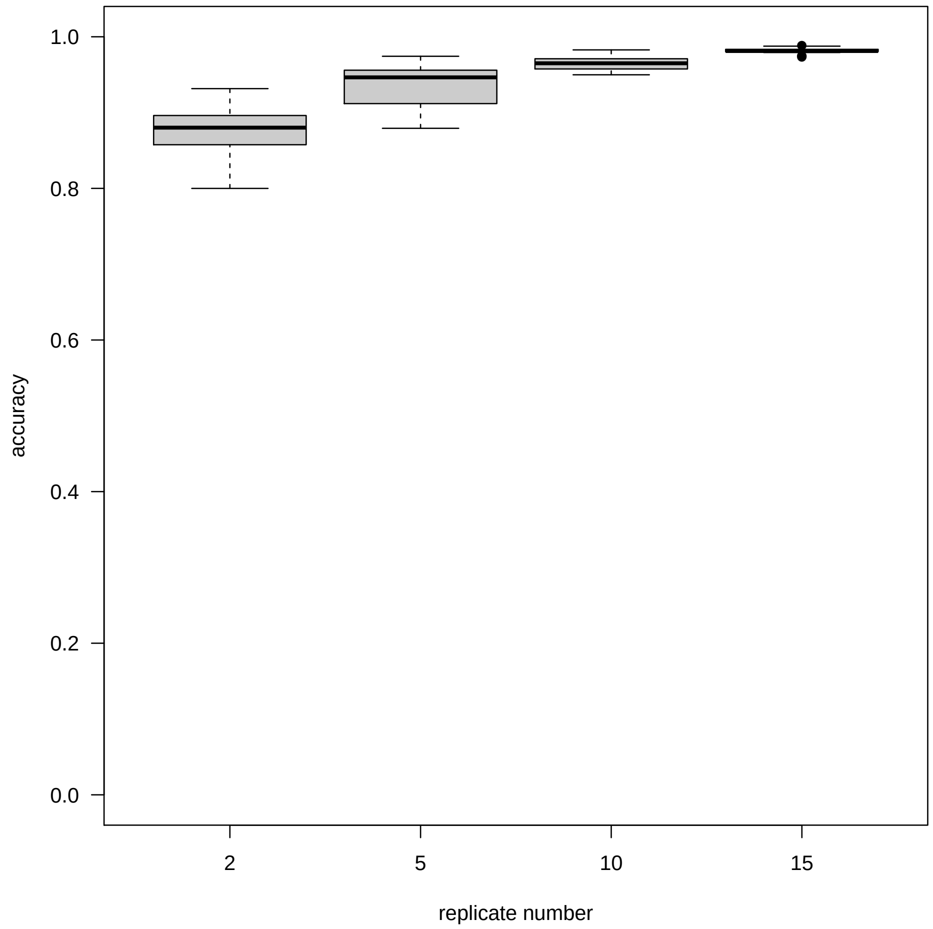
1.0
0.8
0.6
0.4
0.2
0.0
accuracy
2
5
10
15
replicate number
